# Bacillamidins A–G from a Marine-Derived *Bacillus pumilus*

**DOI:** 10.3390/md16090326

**Published:** 2018-09-11

**Authors:** Si-Yu Zhou, Yi-Jie Hu, Fan-Cheng Meng, Shen-Yue Qu, Rui Wang, Raymond J. Andersen, Zhi-Hua Liao, Min Chen

**Affiliations:** 1 Key Laboratory of Luminescent and Real-Time Analytical Chemistry (Ministry of Education), College of Pharmaceutical Sciences, Southwest University, Chongqing 400715, China; vividysz@sina.com (S.-Y.Z.); ejeahoo@live.com (Y.-J.H.); Jianchi_lixiang@163.com (F.-C.M.); 15882349287@163.com (S.-Y.Q.); liuchishou@sina.com (R.W.); 2 Department of Chemistry, University of British Columbia, Vancouver, BC V6T1Z1, Canada; raymond.andersen@ubc.ca; 3 School of Life Sciences, Southwest University, Chongqing 400715, China; zhliao@swu.edu.cn

**Keywords:** *Bacillus pumilus*, long-chain amides, cytotoxic activity, antimicrobial activity

## Abstract

Seven long-chain amides, including five previously undescribed bacillamidins A–E (**1**–**5**) and two previously reported synthetic analogs, bacillamidins F (**6**) and G (**7**), were isolated from extracts of the marine-derived *Bacillus pumilus* strain RJA1515. The structures of the new compounds were established by extensive analysis of 1D and 2D nuclear magnetic resonance (NMR) data as well as high resolution mass spectrometry (HRMS), and the absolute configurations of the stereogenic carbons of **1**–**4** were established by comparison of the calculated and the experimental electronic circular dichroism (ECD) spectra. The cytotoxic and antimicrobial activities of **1**–**7** were evaluated.

## 1. Introduction

Marine microorganisms have become a promising source of structurally diverse and bioactive compounds [1,2]. Marine *Bacillus* species, which are ubiquitous in the marine ecosystem [3], can produce versatile secondary metabolites that exhibit a wide range of biological activities, such as antimicrobial, anticancer, and antialgal activities [4,5,6,7]. The emergence of microbial resistance to currently available antibiotics makes the treatment of infectious diseases more challenging, and the development of new antimicrobial agents is an urgent need [8].

**Figure 1:**
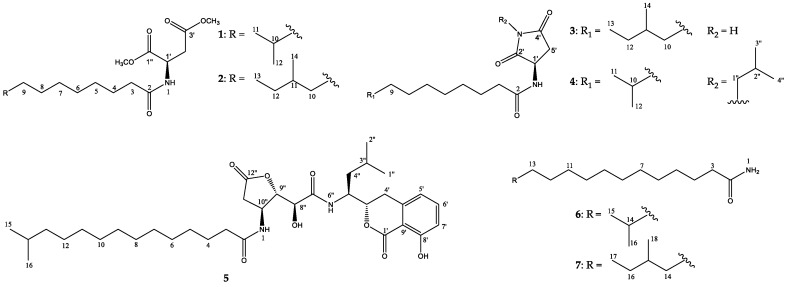
Structures of **1**–**7**.

In a previous study by our group, the ethyl acetate (EtOAc) extract of the culture broth of the strain RJA1515, identified as *Bacillus pumilus*, showed potent antimicrobial activity against Gram-negative bacteria through the inhibition of the enzyme citrate synthase type II [9]. Our ongoing chemical investigation on the same strain led to the isolation of seven long-chain amides including five new compounds, bacillamidins A–E (**1**–**5**), and two previously reported synthetic analogs, bacillamidins F (**6**) and G (**7**) (Figure 1). This paper describes the isolation, the structural elucidation, and the *in vitro* cytotoxicity and antibacterial activity assays of the isolated compounds.

## 2. Results and Discussion

The strain *B. pumilus* RJA1515 was cultured in solid agar for 14 days, then the bacterial cultures and the agar were extracted with EtOAc to afford a crude ethyl acetate extract. The extract was fractionated and purified by repeated column chromatography to give bacillamidins A–G (**1**–**7**).

Compound **1** was obtained as a white powder. The molecular formula was determined as C_17_H_31_NO_5_ by high resolution electrospray ionization mass spectrometry (HRESIMS) (*m*/*z* 352.2096 [M + Na]^+^, calcd. 352.2100), which indicated three degrees of unsaturation. The ^1^H NMR spectrum (Table 1, Figure S1) displayed an ABX spin system at *δ*_H_ 4.60 (1H, ddd, *J* = 9.6, 7.6, 6.0 Hz), 2.79 (1H, dd, *J* = 16.4, 9.6 Hz), 2.67 (1H, dd, *J* = 16.4, 6.0 Hz), a proton doublet at *δ*_H_ 8.31 (1H, d, *J* = 7.2 Hz), two methyl doublets at *δ*_H_ 0.84 (6H, d, *J* = 6.4 Hz) and two methoxyl groups at *δ*_H_ 3.61 (3H, s) and 3.60 (3H, s), as well as numerous methylene protons from *δ*_H_ 1.13 to 2.08. The ^13^C NMR and heteronuclear single quantum correlation (HSQC) data of **1** (Figures S2 and S3) indicated the presence of two methoxyl carbons (*δ*_C_ 51.6, 52.0), three ester or amide carbonyls (*δ*_C_ 170.4, 171.2, 172.2), two methine sp^3^ (*δ*_C_ 27.3, 48.4), eight methylene sp^3^ (*δ*_C_ 25.1, 26.7, 28.4, 28.7, 29.2, 34.9, 35.6, 38.4) and two methyl (*δ*_C_ 22.5) carbons.

**Figure 2:**
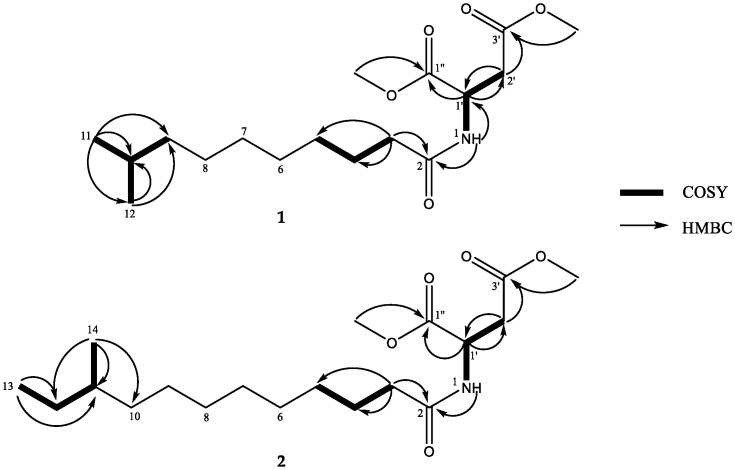
Key ^1^H-^1^H COSY and HMBC correlations for **1** and **2**.

The presence of an alkanone moiety was corroborated by hydrogen-hydrogen correlation spectroscopy (^1^H-^1^H COSY) and heteronuclear multiple bond correlation (HMBC) correlations (Figure 2, Figures S4 and S5). The ^1^H-^1^H COSY spectrum showed correlations from the methyl doublet at *δ*_H_ 0.84 (CH_3_-11 and CH_3_-12) to H-10 (*δ*_H_ 1.48), thus, a *gem*-dimethyl terminus was established (Table 1). An alkyl chain moiety with eleven carbon atoms was established by HMBC correlations from H-3 (*δ*_H_ 2.08, t, *J* = 7.6 Hz) to C-4 (*δ*_C_ 25.1) and C-5 (*δ*_C_ 28.4) and from the terminal *gem*-dimethyl protons (CH_3_-11 and CH_3_-12) to C-9 (*δ*_C_ 38.4), as well as the presence of a broad singlet, integrated for sixteen methylene protons (H_2_-3 to H_2_-10). The HMBC correlation from H-3 to C-2 (*δ*_C_ 172.2) suggested the connectivity of an alkyl chain to the C-2 amide carbonyl carbon. An amide group was confirmed by the HMBC correlation from the proton doublet of H-1 at *δ*_H_ 8.31 to C-2. The HMBC correlations from H-1 to C-1′ (*δ*_C_ 48.4), from H-1′ (*δ*_H_ 4.60) to C-1″ (*δ*_C_ 170.4) and C-2′ (*δ*_C_ 35.6), and from H-2′a (*δ*_H_ 2.79)/H-2′b (*δ*_H_ 2.67) to C-1′ and C-3′ (*δ*_C_ 171.2) suggested the presence of the aspartic acid moiety [10]. In addition, the HMBC correlations from the two methoxyl groups at *δ*_H_ 3.61 and *δ*_H_ 3.60 to C-1″ and C-3′, respectively, indicated the locations of two methyl ester groups.

**Figure 3:**
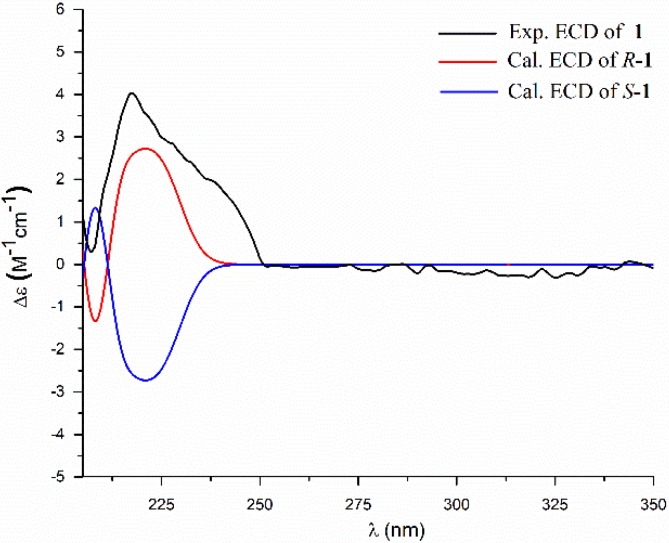
Comparison of the experimental and the calculated ECD spectra of **1**.

The absolute configuration of C-1′ was defined as *R* by comparison of the experimental optical rotation (OR) of **1** ([α]${}_{D}^{\mathrm{25}}$ −16.6) with the calculated OR of *R*-**1** ([α]${}_{D}^{\mathrm{25}}$ −15.1), and by comparison of the calculated and the experimental ECD spectra (Figure 3). Thus, the structure of **1** was determined as (1′*R*)-9-methyldecanoyl dimethylaspartate, named bacillamidin A.

**Figure 4:**
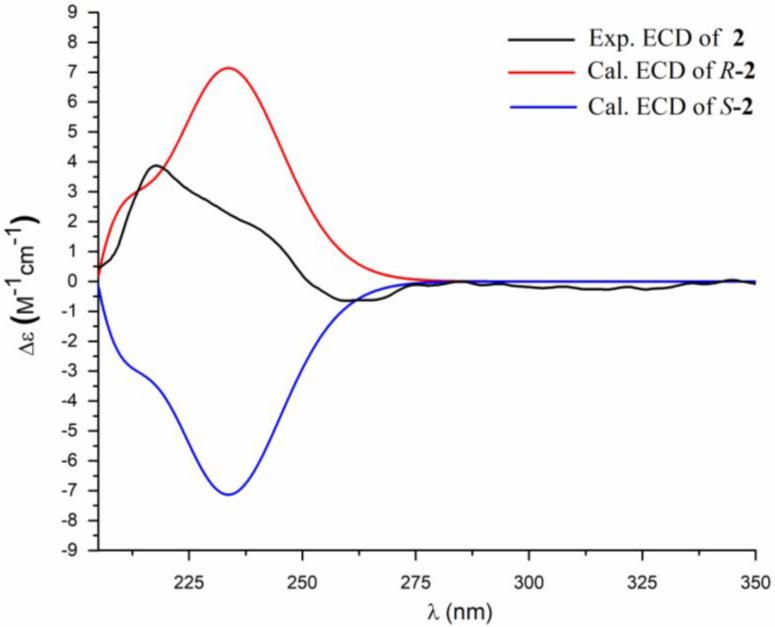
Comparison of the experimental and the calculated ECD spectra of **2**.

Compound **2** was obtained as a white powder. Its molecular formula was established as C_19_H_35_NO_5_ by HRESIMS (*m*/*z* 380.2409 [M + Na]^+^, calcd. 380.2413), implying three degrees of unsaturation. The ^1^H and ^13^C NMR spectra of **2** (Table 1, Figures S9 and S10) resemble those of **1** except for the presence of two additional methylene groups (*δ*_H_ 1.23; *δ*_C_ 29.9, 29.0), one methyl triplet at *δ*_H_ 0.83 (3H, t, *J* = 9.0 Hz), and one methyl doublet *δ*_H_ 0.78 (3H, d, *J* = 9.0 Hz). The ^1^H-^1^H COSY cross-peaks from H-11 (*δ*_H_ 1.23, m) to H-12 (*δ*_H_ 1.10, m) and H-14 (*δ*_H_ 0.78, d, *J* = 9.0 Hz) and from H-12 to H-13 (*δ*_H_ 0.83, t, *J* = 9.0 Hz) and the HMBC correlations (Figure 2, Figures S12 and S13) from H-13 to C-12 (*δ*_C_ 29.0) and C-11 (*δ*_C_ 33.7) and from H-14 to C-10 (*δ*_C_ 36.0), C-11, and C-12 indicated that **2** had a terminal *sec*-butyl group in the alkyl chain. All the aforementioned information indicated the presence of a 10-methyldodecanoyl motif in **2**, instead of the 9-methyldecanoyl moiety in **1**. The absolute configuration of C-1′ was established by comparison of the calculated and the experimental ECD spectra (Figure 4). However, the absolute configuration of C-11 was not determined. Thus, the structure of **2** was determined as (1′*R*)-10-methyldodecanoyl dimethylaspartate, named bacillamidin B.

**Figure 5:**
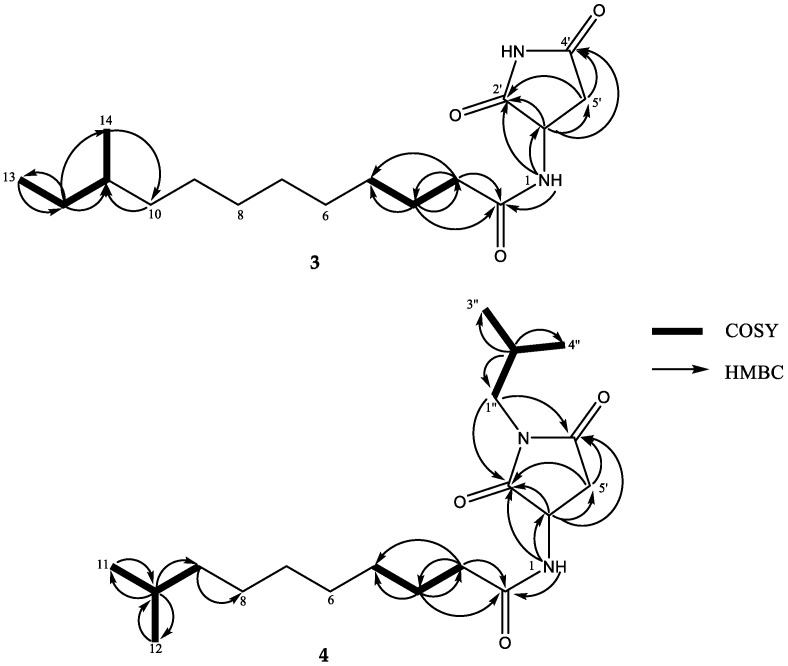
Key ^1^H-^1^H COSY and HMBC correlations for **3** and **4**.

**Figure 6:**
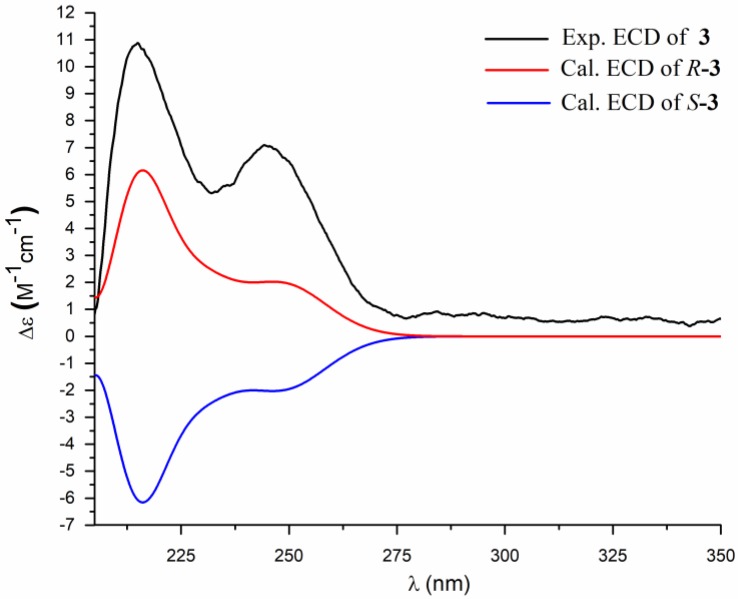
Comparison of the experimental and the calculated ECD spectra of **3**.

Compound **3** was obtained as a white powder. Its molecular formula was determined as C_17_H_30_N_2_O_3_ by HRESIMS (*m*/*z* 333.2145 [M + Na]^+^, calcd. 333.2154), indicating four degrees of unsaturation. The ^1^H NMR spectrum (Table 2, Figure S17) displayed an ABX spin system at *δ*_H_ 4.35 (1H, ddd, *J* = 9.6, 7.6, 6.0 Hz), 2.84 (1H, dd, *J* = 16.4, 9.6 Hz), 2.53 (1H, dd, *J* = 16.4, 6.0 Hz), a proton doublet at *δ*_H_ 8.42 (1H, d, *J* = 7.6 Hz), one methyl triplet at *δ*_H_ 0.83 (3H, t, *J* = 9.0 Hz), one methyl doublet *δ*_H_ 0.78 (3H, d, *J* = 9.0 Hz), and a singlet of one proton (*δ*_H_ 11.19, s), as well as numerous methylene protons from *δ*_H_ 1.13 to 1.23. The ^13^C NMR and HSQC data of **3** (Figures S18 and S19) indicated the presence of three ester or amide carbonyls (*δ*_C_ 172.5, 176.4, 177.7), as well as two methine sp^3^ (*δ*_C_ 33.7, 49.5), ten methylene sp^3^ (*δ*_C_ 25.0, 26.5, 28.5, 28.8, 29.0, 29.0, 29.3, 34.9, 36.0, 36.2), and two methyl carbons (*δ*_C_ 11.2, 19.1). The HMBC correlations from H-1′ (*δ*_H_ 4.35) to C-2′ (*δ*_C_ 177.7), C-4′ (*δ*_C_ 176.4), and C-5′ (*δ*_C_ 36.0) and from H-5′a (*δ*_H_ 2.84)/H-5′b (*δ*_H_ 2.53) to C-4′ and C-2′, in addition to a NH singlet (*δ*_H_ 11.19), suggested the presence of a succimide substructure [11]. The HMBC correlations from NH-1 (*δ*_H_ 8.42, d, 7.6 Hz) to C-1′ (*δ*_C_ 49.5) and C-2′ indicated the linkage between the amide and succimide portion. The ^1^H-^1^H COSY and HMBC spectra (Figure 5, Figures S20 and S21) of **3** indicated the same alkyl side chain as a terminal *sec*-butyl group in **2**. The absolute configuration of C-1′ was established by comparison of the calculated and the experimental ECD spectra (Figure 6). The absolute configuration of C-11 was not determined at a current state. Thus, the structure of **3** was determined as (3′*R*)-*N*-(2, 5-dioxopyrrolidin-3-yl)-10-methyldodecamide, named bacillamidin C.

**Figure 7:**
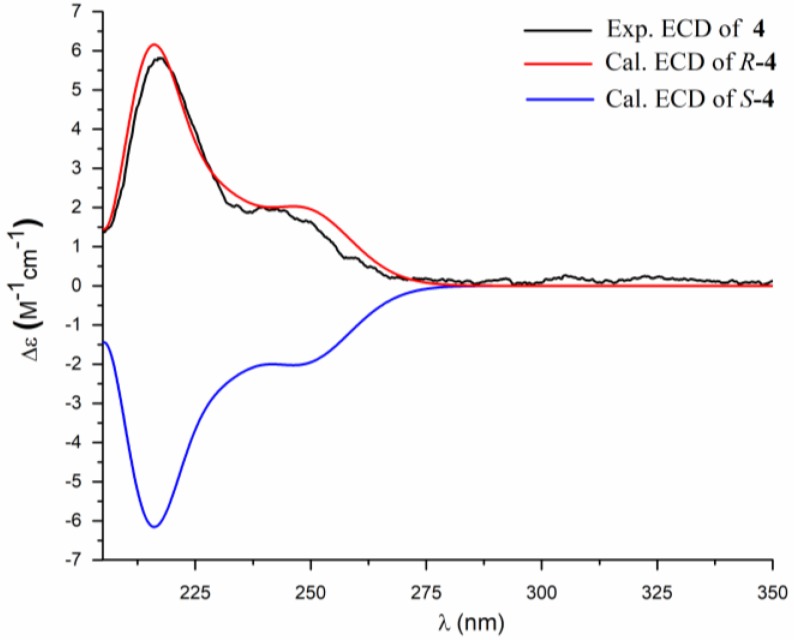
Comparison of the experimental and the calculated ECD spectra of **4**.

Compound **4** was obtained as a white powder. The molecular formula was determined as C_19_H_34_N_2_O_3_ according to its HRESIMS data (*m*/*z* 361.2461 [M + Na]^+^, calcd. 361.2467). The ^1^H and ^13^C NMR, ^1^H-^1^H COSY and HMBC spectra (Table 2, Figures S25, S26, S28 and S29) showed that the alkyl side chain of **4** possessed a *gem*-dimethyl terminus, similar to that of **1**. The rest ^1^H and ^13^C NMR data of **4** was very similar to those of **3** except for the absence of the NH singlet at *δ*_H_ 11.19 and the presence of a *sec*-butyl moiety (two methyl doublets at *δ*_H_ 0.84, *J* = 6.6 Hz/*δ*_C_ 20.0, a multiplet at *δ*_H_ 1.90, /*δ*_C_ 26.7, and a doublet at *δ*_H_ 3.19, *J* = 7.2 Hz/*δ*_C_ 45.4). Therefore, the structure of **4** was established as shown in Figure 3. The absolute configuration of C-1′ was established by comparison of the calculated and the experimental ECD spectra (Figure 7). Therefore, the structure of **4** was determined as (3′*R*)-*N*-(1-isobutyl-2, 5-dioxopyrrolidin-3-yl)-9-methyldecanamide, named bacillamidin D.

**Figure 8:**
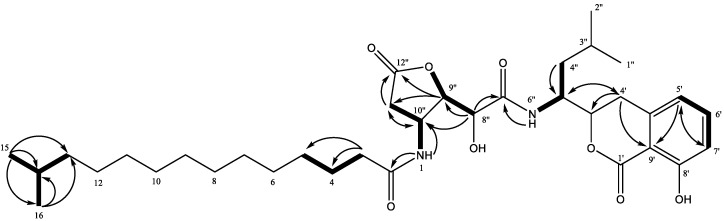
Key ^1^H-^1^H COSY and HMBC correlations for **5**.

Compound **5** was obtained as a white powder and its molecular formula was established as C_35_H_54_N_2_O_5_ by HRESIMS (*m*/*z* 653.3775 [M + Na]^+^, calcd. 653.3778), implying ten degrees of unsaturation. The UV spectrum of **5** showed absorption maxima at 248 and 300 nm which are characteristic of the amicoumacins [12,13]. The ^1^H NMR spectrum (Table 3, Figure S33) exhibited the signals of the protons of the 1,2,3-trisubstituted benzene ring at *δ*_H_ 7.48 (1H, dd, *J* = 8.4, 7.6 Hz), 6.84 (1H, d, *J* = 8.4 Hz), and 6.82 (1H, d, *J* = 7.6 Hz), three sp^3^ oxymethines at *δ*_H_ 4.69 (1H, ddd, *J* = 10.4, 8.0, 2.8 Hz, H-3′), 4.61 (1H, dd, *J* = 2.4, 2.0 Hz, H-9″), and 4.27 (1H, dd, *J* = 6.0, 2.4 Hz, H-8″), two nitrogen-bearing sp^3^ methines at *δ*_H_ 4.32 (1H, m, H-10″) and 4.19 (1H, dddd, 3.6, 7.6, 10.4, 13.6 Hz, H-5″), a broad peak at *δ*_H_ 1.19 (12H, H-5~H-10), and three doublets of four methyl groups at *δ*_H_ 0.88 (H-2″), 0.83 (H-15, H-16), and 0.80 (H-1″). The ^13^C NMR spectrum (Figure S34) exhibited four ester or amide carbonyls (*δ*_C_ 168.9, 169.8, 171.9, 175.6), three quaternary sp^2^ (*δ*_C_ 108.3, 140.4, 160.8), three methine sp^2^ (*δ*_C_ 115.2, 118.5, 136.2), three oxymethine sp^3^ (*δ*_C_ 71.6, 80.8, 85.7), and two nitrogen-bearing methine sp^3^ (*δ*_C_ 46.1, 48.3) carbons. These NMR data were similar to those of amicoumacin C [14]. The gross structure of **5** was therefore established based on the HSQC and HMBC correlations (Figure 8), indicating that an amicoumacin unit was linked to an alkanone side chain with a *gem*-dimethyl terminus.

The negative sign of the Cotton effect for the n-π* transition of the carbonyl group at 260 nm in the ECD spectrum (Figure S42) was in agreement with the 3′*S* configuration [13]. Moreover, the *J*_H-3′/H-5″_ (10.4 Hz) value was indicative of the trans configuration between H-3′ and H-5″ [13]. In addition, the ECD spectrum of **5** showed a positive Cotton effect at 220 and 242 nm and a negative Cotton effect at 260 and 314 nm, which were in good agreement with those for amicoumacin B and amicoumacin C [15]. Thus, the structure of **5** was determined as (3′*S*,5″*S*,8″*S*,9″*S*,10″*S*)-8′,8″-dihydroxy-9″-oxotetrahydrofuran-10″-yl-13-methyltetradecanamide, named bacillamidin E.

**Figure 9:**
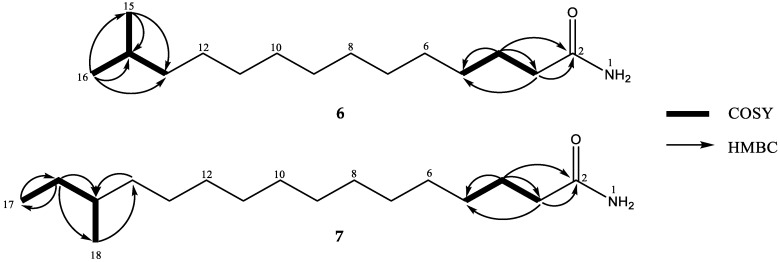
Key ^1^H-^1^H COSY and HMBC correlations for **6** and **7**.

Compounds **6** and **7** were obtained as white powders. Their molecular formulas were determined as C_15_H_31_NO and C_17_H_35_NO according to sodium-adduct ion peaks at *m*/*z* 264.2301 for **6** and 292.2616 for **7**. The structures of **6** and **7** were further established by analyses of their ^1^H, ^13^C, HSQC and HMBC NMR spectral data (Table S1, Figure 9, Figures S43–S46 and S51–S54) as 13-methyltetradecanamide (bacillamidin F) [16], and 14-methylhexadecanamide (bacillamidin G) [17], respectively. The absolute configuration of C-15 of **7** was not determined at current state. Although **6** and **7** have been already synthesized [16,17], this is the first report of their isolation from a natural source.

The cytotoxic activity against human hepatoma cell line (HepG2), human lung cancer cell line (A549), human breast cancer cell line (MDA-MB-231), and human gastric cancer cell line (SGC7901) and the antibacterial activity against *Pseudomonas aeruginosa* PA-01, *Acinetobacter baumannii* ATCC19606 and *Escherichia coli* BW25113 of **1**–**7** were investigated (Table 4). None of the compounds showed significant cytotoxicity in all the cell lines tested. Compounds **1**–**4** showed antibacterial activity against *P. aeruginosa* PA-01 and *A. baumannii* ATCC19606 with minimum inhibitory concentration (MIC) values ranging from 58 to 64 μg/mL.

## 3. Materials and Methods

### 3.1. General Experimental Procedures

The melting points were measured on a SGWX-4A microscope (Shanghai Physical-Optics Instruments Co., Shanghai, China). UV spectra were recorded with a Shimadzu UV-260 spectrophotometer (Shimadzu Corp., Kyoto, Japan) in MeOH; *λ*_max_ (log *ε*) in nm. IR spectra were recorded with KBr pellets on an Avatar 360 E.S.P spectrophotometer (Thermo Nicolet Co., Boston, MA, USA). NMR spectra were recorded on a Bruker DRX500 (400 MHz for ^1^H, 100 MHz for ^13^C) spectrometer (Bruker, Bremen, Germany) in DMSO-*d_6_*; *δ* in ppm rel. with Me_4_Si as internal references, and *J* in Hz. HR-ESI-MS was measured with a Xevo G2-S Q-Tof MS (Waters MS Technologies, Manchester, UK) in positive-ion mode; in *m*/*z* (rel. %). ECD measurements were performed using a JASCO J-1500 spectropolarimeter (Jasco Co., Ltd., Tokyo, Japan). Column chromatography (CC) was performed with silica gel (200–300 mesh, Qingdao Marine Chemical Factory, Qingdao, China), Sephadex LH-20 gel (GE Healthcare Bio-Sciences AB, Uppsala, Sweden), and RP silica gel (ODS-A-HG, YMC, Kyoto, Japan). TLC was performed on silica gel plates GF_254_ (Yan-tai Institute of Chemical Technology, Yantai, China). The spots on TLC were visualized by UV light (254 nm) and sprayed with 10% H_2_SO_4_, followed by heating. Semi-preparative high performance liquid chromatography (HPLC) was carried out on a Shimadzu LC-6AD chromatograph (Shimadzu Corp., Kyoto, Japan) with an ODS column (*RP-18*, 250 × 10 mm, YMC, 5 μm) with a flow rate of 2.0 mL/min.

### 3.2. Strain and Cultivation

*Bacillus pumilus* strain RJA1515 was isolated from marine sediments collected in Bamfield, British Columbia at a depth of 84 m by Raymond J. Andersen’s group, in January 2010. The strain was cultured on solid agar at room temperature in 160 pans measuring 50 cm × 30 cm, equivalent to 6.8 L volume of the marine medium 1 (MM1) [18]. After 14 days, the bacterial cultures and the agar were extracted twice with EtOAc (2 × 80 L). Then the extracts were re-dissolved in 2.5 L of EtOAc and back extracted three times with 600 mL of water after being combined and dried in vacuo. The organic fraction was then dried in vacuo to give an extract (32.0 g).

### 3.3. Extraction and Isolation

The EtOAc extract (32.0 g) was subjected to an open silica gel column (900.0 g) and eluted with a step gradient of petroleum ether (PE) and EtOAc to yield fourteen fractions (Fr. A–N). The PE/EtOAc (7:3) fraction Fr. H (250.1 mg) was purified on a Sephadex LH-20 column and eluted with CHCl_3_/MeOH (1:3) to give fractions H1 and H2. Fraction H1 (141.3 mg) was fractionated by RP-18 CC (MeOH-H_2_O, from 20:80 to 100:0) to afford fractions H1/1–H1/5. Fraction H1/4 (43.8 mg) was further separated on semi-preparative HPLC with MeOH/H_2_O (0–35 min: 87:13) as eluent to yield **1** (2.2 mg, *t_R_* 15.1 min) and **2** (2.6 mg, *t_R_* 9.0 min). Fraction M (PE-EtOAc, 1:1, 410.8 mg) was chromatographed on RP silica gel CC (25.0 g) with a step gradient of MeOH-H_2_O (20:80–100:0) to obtain fractions M1-5. Fraction M4 was purified by preparative HPLC with MeOH/H_2_O (0–60 min: 78:22) to give **3** (2.1 mg, *t_R_* 42.1 min) and **4** (5.1 mg, *t_R_* 34.9 min). Fraction N (PE-EtOAc, 3:7, 458.9 mg) was chromatographed on RP silica gel CC (25.0 g) with a step gradient of MeOH-H_2_O (20:80–100:0) to give fractions N1-5. Fraction N5 was further purified by semi-preparative HPLC with MeOH/H_2_O (74:26) to obtain **5** (2.8 mg, *t_R_* 25.1 min). Fraction J (PE-EtOAc, 6:4, 699.3 mg) was chromatographed on RP silica gel CC (25.0 g) with a step gradient of MeOH-H_2_O (20:80–100:0) to give five fractions. Fraction J5 (46.4 mg) was further purified by preparative HPLC with MeOH/H_2_O (0–60 min: 87:13) to yield **6** (9.9 mg, *t_R_* 18.3 min) and **7** (3.6 mg, *t_R_* 21.3 min).

Bacillamidin A (**1**): amorphous, white powder; m.p.: 86.8–88.2 °C; [α]${}_{D}^{\mathrm{25}}$ = −16.6 (*c* 0.20, MeOH); ECD (c = 1.08 mM, MeOH) *λ*_max_ (Δ*ε*): 218 (+4.12); UV (MeOH) *λ*_max_ (log *ε*) 225 (2.85) nm; IR (KBr) *ν*_max_ 3314, 2918, 2851, 1749, 1732, 1649, 1564, 1545, 1435, 1302, 1242, 1163 cm^−1^; ^1^H and ^13^C NMR data, Table 1; HRESIMS *m*/*z* 352.2096 [M + Na]^+^ (calcd. for C17H31NO5Na, 352.2100).

Bacillamidin B (**2**): amorphous, white powder; m.p.: 85.7–87.1 °C; [α]${}_{D}^{\mathrm{25}}$ = −16.0 (*c* 0.20, MeOH); ECD (c = 1.02 mM, MeOH) *λ*_max_ (Δ*ε*): 215 (+5.90); UV (MeOH) *λ*_max_ (log *ε*) 225 (2.47) nm; IR (KBr) *ν*_max_ 3314, 2961, 2918, 2851, 1751, 1732, 1647, 1560, 1543, 1435, 1302, 1242, 1211, 1163 cm^−1^; ^1^H and ^13^C NMR data, Table 1; HRESIMS *m*/*z* 380.2409 [M + Na]^+^ (calcd. for C_19_H_35_NO_5_Na, 380.2313).

Bacillamidin C (**3**): amorphous, white powder; m.p.: 87.8–89.2 °C; [α]${}_{D}^{\mathrm{25}}$ = −26.1 (*c* 0.20, MeOH); ECD (c = 2.24 mM, MeOH) *λ*_max_ (Δ*ε*): 215 (+10.88), 245 (+7.06); UV (MeOH) *λ*_max_ (log *ε*) 226 (2.62) nm; IR (KBr) *ν*_max_ 3238, 2961, 2922, 2851, 1722, 1703, 1655, 1524, 1416, 1373, 1196, 1171 cm^−1^; ^1^H and ^13^C NMR data, Table 2; HRESIMS *m*/*z* 333.2145 [M + Na]^+^ (calcd. for C_17_H_30_N_2_O_3_Na, 333.2154).

Bacillamidin D (**4**): amorphous, white powder; m.p.: 91.4–92.6 °C; [α]${}_{D}^{\mathrm{25}}$ = −24.3 (*c* 0.20, MeOH); ECD (c = 2.00 mM, MeOH) *λ*_max_ (Δ*ε*): 218 (+5.60), +245 (+1.90); UV (MeOH) *λ*_max_ (log *ε*) 226 (2.49) nm; IR (KBr) *ν*_max_ 3238, 2961, 2922, 2851, 1722, 1659, 1520, 1196, 1171 cm^−1^; ^1^H and ^13^C NMR data, Table 2; HRESIMS *m*/*z* 361.2461 [M + Na]^+^ (calcd. for C_19_H_34_N_2_O_3_Na, 361.2467).

Bacillamidin E (**5**): amorphous, white powder; m.p.: 92.3–93.9 °C; [α]${}_{D}^{\mathrm{25}}$ = −42.0 (*c* 0.20, MeOH); ECD (c = 0.59 mM, MeOH) *λ*_max_ (Δ*ε*): 220 (+1.49), 226 (−0.47), 242 (+4.48), 260 (−3.92), 314 (−0.74), +341 (0.15); UV (MeOH) *λ*_max_ (log ε) 248 (3.47), 300 (2.95) nm; IR (KBr) *ν*_max_ 3452, 2922, 2853, 1686, 1649, 1566, 1555, 1468, 1412 cm^−1^; ^1^H and ^13^C NMR data, Table 3; HRESIMS *m*/*z* 653.3775 [M + Na]^+^ (calcd. for C_35_H_54_N_2_O_8_Na, 653.3778).

### 3.4. ECD Calculation Methods

In general, conformational analyses were carried out via random searching in the Sybyl-X 2.0 using the MMFF94S force field with an energy cutoff of 2.5 kcal/mol. The results showed five lowest energy conformers for **1**–**3**, and six for **4**. Subsequently, the conformers were re-optimized using density functional theory (DFT) at the b3lyp/6-31+g(d) level in gas phase by the GAUSSIAN 09 program. The energies, oscillator strengths, and rotational strengths (velocity) of the first 60 electronic excitations were calculated using the time-dependent DFT methodology at the b3lyp/6-311++g(d,p) level in vacuo. The ECD spectra were simulated by the overlapping Gaussian function (half the bandwidth at 1/e peak height, σ = 0.30 for **1** and **2**, σ = 0.16 for **3**, σ = 0.25 for **4**).

To get the final spectra, the simulated spectra of the conformers were averaged according to the Boltzmann distribution theory and their relative Gibbs free energy (Δ*G*). By comparing the experiment spectrum with the calculated ECD model molecules, the absolute configuration of the only chiral center of all the four compounds was determined to be *R*.

### 3.5. Bioassay

Cytotoxicity assays were evaluated on the human tumor cell lines HepG2 (human hepatocellular carcinoma cell line), A549 (human lung carcinoma cell line), MDA231 (human breast carcinoma cells), and SGC7901 (human gastric carcinoma cells) by MTT method according to our previous study [19].

Antimicrobial activity was measured by the liquid broth microdilution method [20] against *Pseudomonas aeruginosa* PA-01, *Acinetobacter baumannii* ATCC19606, and *Escherichia coli* BW25113, with ofloxacin as the positive control.

## 4. Conclusions

Microorganisms from marine sediments are promising sources of bioactive natural products. Chemical investigation of the marine-derived *Bacillus pumilus* RJA1515 led to the isolation and identification of five new long-chain amides (**1**–**5**) and two previously reported long-chain alkylamides (**6**, **7**). The absolute configuration of **1** was determined by comparison of the calculated and the experimental OR and ECD spectra, while those of **2–5** were determined by comparison of their calculated and the experimental ECD spectra. Compounds **1**–**4** showed antibacterial activity against *P. aeruginosa* PA-01 and *A. baumannii* ATCC19606 with MIC values ranging from 58 to 64 μg/mL.

## Figures and Tables

**Table 1:** ^1^H and ^13^C NMR data (400 and 100 MHz, in DMSO-*d_6_*) of **1** and **2**.

Position	1		2	
	*δ*_H_ (*J*, Hz)	*δ*_C_, Type	*δ*_H_ (*J*, Hz)	*δ*_C_, Type
1-NH	8.31, d (7.6)		8.42, d (7.6)	
2		172.2, CO		172.2, CO
3	2.08, t (7.6)	34.9, CH_2_	2.08, t (7.6)	34.9, CH_2_
4	1.48, m	25.1, CH_2_	1.47, m	25.1, CH_2_
5	1.23, m	28.4, CH_2_	1.23, m	28.4, CH_2_
6	1.23, m	28.7, CH_2_	1.23, m	28.7, CH_2_
7	1.23, m	29.2, CH_2_	1.23, m	28.9, CH_2_
8	1.23, m	26.7, CH_2_	1.23, m	29.3, CH_2_
9	1.13, m	38.4, CH_2_	1.23, m	26.4, CH_2_
10	1.48, m	27.4, CH	1.09, m; 1.27, m	36.0, CH_2_
11	0.84, d (6.4)	22.5, CH_3_	1.30, m	33.7, CH
12	0.84, d (6.4)	22.5, CH_3_	1.10, m	29.0, CH_2_
13			0.83, t (9.0)	11.2, CH_3_
14			0.78, d (9.0)	19.1, CH_3_
1′	4.60, ddd (9.6, 7.6, 6.0)	48.4, CH	4.60, ddd (9.6, 7.6, 6.0)	48.4, CH
2′	2.79, dd (16.4, 9.6)	35.6, CH_2_	2.79, dd (16.4, 9.6)	35.6, CH_2_
	2.67, dd (16.4, 6.0)		2.67, dd (16.4, 6.0)	
3′		171.2, CO		171.3, CO
3′-OCH_3_	3.61, s	51.6, OCH_3_	3.61, s	51.6, OCH_3_
1″		170.4, CO		170.4, CO
1″-OCH_3_	3.60, s	52.0, OCH_3_	3.60, s	52.1, OCH_3_

**Table 2:** ^1^H and ^13^C-NMR data (400 and 100 MHz, in DMSO-*d_6_*) of **3** and **4**.

Position	3		4	
	*δ*_H_ (*J*, Hz)	*δ*_C_, Type	*δ*_H_ (*J*, Hz)	*δ*_C_, Type
1-NH	8.42, d (7.6)		8.51, d (7.6)	
2		172.5, CO		172.6, CO
3	2.08, t (7.6)	34.9, CH_2_	2.08, t (7.6)	34.8, CH_2_
4	1.23, m	25.0, CH_2_	1.48, m	25.0, CH_2_
5	1.23, m	28.5, CH_2_	1.23, m	28.4, CH_2_
6	1.23, m	28.8, CH_2_	1.23, m	28.8, CH_2_
7	1.23, m	29.0, CH_2_	1.23, m	29.2, CH_2_
8	1.23, m	29.3, CH_2_	1.23, m	26.7, CH_2_
9	1.23, m	26.5, CH_2_	1.13, m	38.4, CH_2_
10	1.09, m; 1.27, m	36.2, CH_2_	1.48, m	27.4, CH
11	1.30, m	33.7, CH	0.83, d (6.6)	22.5, CH_3_
12	1.10, m	29.0, CH_2_	0.83, d (6.6)	22.5, CH_3_
13	0.83, t (9.0)	11.2, CH_3_		
14	0.78, d (9.0)	19.1, CH_3_		
1′	4.35, ddd (9.6, 7.6, 6.0)	49.5, CH	4.35, ddd (9.6, 7.6, 6.0)	48.4, CH
2′		177.7, CO		176.5, CO
3′-NH	11.19, s			
4′		176.4, CO		175.2, CO
5′	2.84, dd (16.4, 9.6)	36.0, CH_2_	2.91, dd (16.4, 9.6)	34.8, CH_2_
	2.53, dd (16.4, 6.0)		2.53, dd (16.4, 6.0)	
1″			3.19, d (7.2)	45.4, CH_2_
2″			1.90, m	26.7, CH
3″			0.84, d (6.6)	20.0, CH_3_
4″			0.84, d (6.6)	20.0, CH_3_

**Table 3:** ^1^H and ^13^C-NMR data (400 and 100 MHz, in DMSO-*d_6_*) of **5**.

Position	5				
	*δ*_H_ (*J*, Hz)	*δ*_C_, Type	Position	*δ*_H_ (*J*, Hz)	*δ*_C_, Type
1-NH	8.34, d (6.8)		1″	0.80, d (6.4)	21.3, CH_3_
2		171.9, CO	2″	0.88, d (6.4)	23.3, CH_3_
3	1.90, t (7.6)	35.0, CH_2_	3″	1.56, m	24.0, CH
4	1.48, m	24.9, CH_2_	4″	1.32, m	38.4, CH_2_
5-10	1.25, m	28.5–29.2		1.68, m	
11	1.25, m	29.3, CH_2_	5″	4.19, dddd (3.6, 7.6, 10.4, 13.6)	48.3, CH
12	1.25, m	26.7, CH_2_	6″-NH	7.94, d (9.6)	
13	1.13, m	38.4, CH_2_	7″		169.8, CO
14	1.49, m	27.4, CH	8″	4.27, dd (6.0, 2.4)	71.6, CH
15	0.83, d, (6.6)	22.5, CH_3_	8″-OH	6.18, d (6.0)	
16	0.83, d, (6.6)	22.5, CH_3_	9″	4.61, dd (2.4, 2.0)	85.7, CH
1′		168.9, CO	10″	4.32, m	46.1, CH
3′	4.69, ddd (10.4, 8.0, 2.8)	80.8, CH	11″	2.16, dd (18.0, 2.2)	36.0, CH_2_
4′	2.87, m	29.0, CH_2_		2.85, m	
5′	6.82, d (7.6)	118.5, CH	12″		175.6, CO
6′	7.48, dd (8.4, 7.6)	136.2, CH			
7′	6.84, d (8.4)	115.2, CH			
8′		160.8, C			
8″-OH	10.79, s				
9′		108.3, C			
10′		140.4, C			

**Table 4:** Antimicrobial activity of **1**–**7** (MIC, μg/mL).

Compounds	*P. aeruginosa PA-01*	*A. baumannii* ATCC19606	*E. coli* BW25113
**1**	64	58	>128
**2**	64	64	>128
**3**	64	64	>128
**4**	64	58	>128
**5**	>128	>128	>128
**6**	>128	>128	>128
**7**	>128	>128	>128
ofloxacin	8	16	1

## Supplementary Materials

The following are available online at http://www.mdpi.com/1660-3397/16/9/326/s1. This section includes 1D, 2D NMR spectra and other spectroscopic data for compounds **1**–**7**.
