# Supplementary material for: Bacillamidins A–G from a Marine-Derived Bacillus pumilus
Source: Mar Drugs. 2018 Sep 11;16(9):326. doi: 10.3390/md16090326 (PMC6164710; doi:10.3390/md16090326)
Supplement: Supplementary file 1 [file marinedrugs-16-00326-s001.pdf]

Supporting Information

Bacillamidins A-G from a Marine-Derived *Bacillus pumilus*

Si-Yu Zhou[1], Yi-Jie Hu[1], Fan-Cheng Meng, Shen-Yue Qu[1], Rui Wang[1], Raymond J. Andersen[2], Zhi-Hua Liao[3] and Min Chen[1,*]

[1] College of Pharmaceutical Sciences, Key Laboratory of Luminescent and Real-Time Analytical Chemistry (Ministry of Education), Southwest University, Chongqing 400715, P.R. China; E-Mails: vividysz@sina.com (S.-Y.Z.), ejeahoo@live.com (Y.-J.H.); (F.-C.M.); (S.-Y.Q.); (R.W.); mminchen@swu.edu.cn (M.C.)

[2] Departments of Chemistry, University of British Columbia, Vancouver, British Columbia, Canada V6T1Z1; raymond.andersen@ubc.ca (R.J.A)

[3] School of Life Sciences, Southwest University, Chongqing 400715, P.R. China; zhliao@swu.edu.cn (Z.-H.L.)

* Correspondence: mminchen@swu.edu.cn; Tel.: +86-023-6825-1225

Contents

OR Calculation Details

ECD Calculation Details

Figure S1. ^{1}H-NMR spectrum of compound **1** (400 MHz, DMSO-d_6)

Figure S2. ^{13}C-NMR spectrum of compound **1** (100 MHz, DMSO-d_6)

Figure S3. HSQC spectrum of compound **1** (400 MHz, DMSO-d_6)

Figure S4. HMBC spectrum of compound **1** (400 MHz, DMSO-d_6)

Figure S5. COSY spectrum of compound **1** (400 MHz, DMSO-d_6)

Figure S6. HR-ESI-MS spectrum of compound **1**

Figure S7. IR spectrum of compound **1**

Figure S8. UV spectrum of compound **1**

Figure S9. ^{1}H-NMR spectrum of compound **2** (400 MHz, DMSO-d_6)

Figure S10. ^{13}C-NMR spectrum of compound **2** (100 MHz, DMSO-d_6)

Figure S11. HSQC spectrum of compound **2** (400 MHz, DMSO-d_6)

Figure S12. HMBC spectrum of compound **2** (400 MHz, DMSO-d_6)

Figure S13. COSY spectrum of compound **2** (400 MHz, DMSO-d_6)

Figure S14. HR-ESI-MS spectrum of compound **2**

Figure S15. IR spectrum of compound **2**

Figure S16. UV spectrum of compound **2**

Figure S17. ^{1}H-NMR spectrum of compound **3** (400 MHz, DMSO-d_6)

Figure S18. ^{13}C-NMR spectrum of compound **3** (100 MHz, DMSO-d_6)

Figure S19. HSQC spectrum of compound **3** (400 MHz, DMSO-d_6)

Figure S20. HMBC spectrum of compound **3** (400 MHz, DMSO-d_6)

Figure S21. COSY spectrum of compound **3** (400 MHz, DMSO-d_6)

Figure S22. HR-ESI-MS spectrum of compound **3**

Figure S23. IR spectrum of compound **3**

Figure S24. UV spectrum of compound **3**

Figure S25. ^{1}H-NMR spectrum of compound **4** (400 MHz, DMSO-d_6)

Figure S26. ^{13}C-NMR spectrum of compound **4** (100 MHz, DMSO-d_6)

Figure S27. HSQC spectrum of compound **4** (400 MHz, DMSO-d_6)

Figure S28. HMBC spectrum of compound **4** (400 MHz, DMSO-d_6)

Figure S29. COSY spectrum of compound **4** (400 MHz, DMSO-d_6)

Figure S30. HR-ESI-MS spectrum of compound **4**

Figure S31. IR spectrum of compound **4**

Figure S32. UV spectrum of compound **4**

Figure S33. ^{1}H-NMR spectrum of compound **5** (400 MHz, DMSO-d_6)

Figure S34. ^{13}C-NMR spectrum of compound **5** (100 MHz, DMSO-d_6)

Figure S35. HSQC spectrum of compound **5** (400 MHz, DMSO-d_6)

Figure S36. HMBC spectrum of compound **5** (400 MHz, DMSO-d_6)

Figure S37. COSY spectrum of compound **5** (400 MHz, DMSO-d_6)

Figure S38. NOESY spectrum of compound **5** (400 MHz, DMSO-d_6)

Figure S39. HR-ESI-MS spectrum of compound **5**

Figure S40. IR spectrum of compound **5**

Figure S41. UV spectrum of compound **5**

Figure S42. ECD spectrum of compound **5**

Figure S43. ^{1}H-NMR spectrum of compound **6** (400 MHz, DMSO-d_6)

Figure S44. ^{13}C-NMR spectrum of compound **6** (100 MHz, DMSO-d_6)

Figure S45. HSQC spectrum of compound **6** (400 MHz, DMSO-d_6)

Figure S46. HMBC spectrum of compound **6** (400 MHz, DMSO-d_6)

Figure S47. COSY spectrum of compound **6** (400 MHz, DMSO-d_6)

Figure S48. HR-ESI-MS spectrum of compound **6**

Figure S49. IR spectrum of compound **6**

Figure S50. UV spectrum of compound **6**

Figure S51. ^{1}H-NMR spectrum of compound **7** (400 MHz, DMSO-d_6)

Figure S52. ^{13}C-NMR spectrum of compound **7** (100 MHz, DMSO-d_6)

Figure S53. HSQC spectrum of compound **7** (400 MHz, DMSO-d_6)

Figure S54. HMBC spectrum of compound **7** (400 MHz, DMSO-d_6)

Figure S55. COSY spectrum of compound **7** (400 MHz, DMSO-d_6)

Figure S56. HR-ESI-MS spectrum of compound **7**

Figure S57. IR spectrum of compound **7**

Figure S58. UV spectrum of compound **7**

Table S1. ^{1}H and ^{13}C-NMR data (400 and 100 MHz, in DMSO-d_6) of **6** and **7**

Spectral Data of 6 and 7

OR Calculation Details

Monte Carlo conformational searches were carried out by means of the Spartan's 10 software using Merck Molecular Force Field (MMFF). The conformers with Boltzmann-population of over 5% were chosen for OR calculations, and then the conformers were initially optimized at B3LYP/6-31+g (d, p) level in MeOH using the CPCM polarizable conductor calculation model. The theoretical calculation of OR was conducted in MeOH using Time-dependent Density functional theory (TD-DFT) at the B3LYP/6-31+g (d, p) level for all conformers of compounds *R*-**1**. Cartesian coordinates for the low-energy reoptimized MMFF conformers of *R*-**1** at B3LYP/6-311+G (d, p) level of theory in CH_3OH.

1. The optimized conformers of *R*-**1**

C1	C2
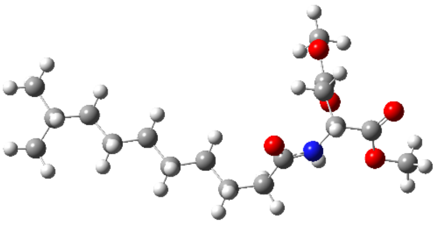	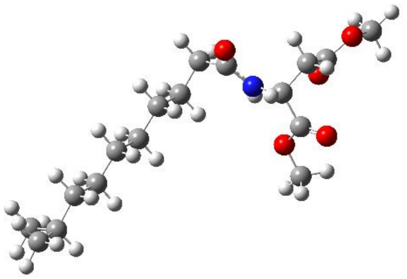
C3	C4
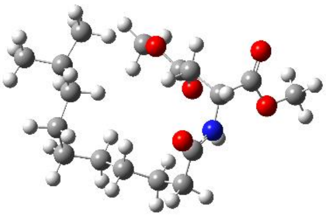	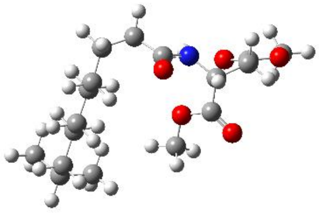
C5	
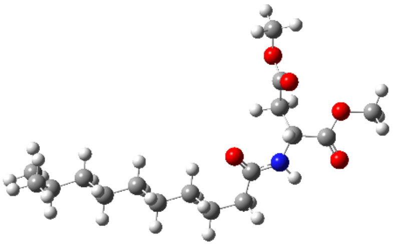	

2. Gibbs free energies[a] and equilibrium populations[b] of low-energy conformers of *R*-**1**:

Conformers	In MeOH	
	Boltzmann population (%)	*OR*
R-**1-**1	60.58	-35.15
R-**1-**2	21.82	31.91
R-**1-**3	1.65	-98.51
R-**1-**4	10.29	-4.4
R-**1-**5	5.66	23.68
average		-15.07

ECD Calculation Details

1. The optimized conformers of *R*-**1**

C1

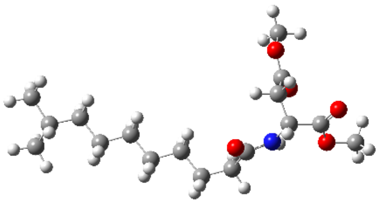

C2

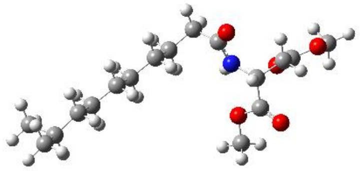

C3

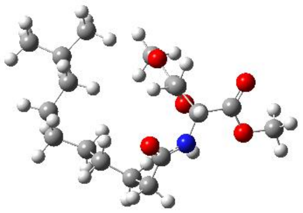

C4

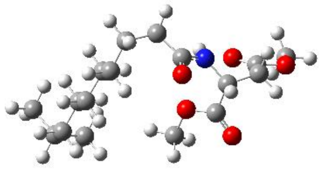

C5

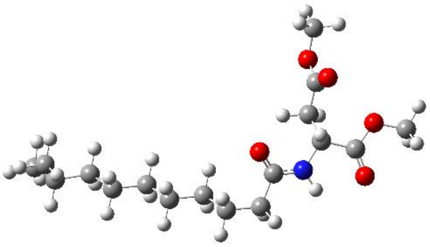

2. B3LYP-calculated relative energies (Kcal/mol) and conformational population (%) for the most stable conformers of *R*-**1**.

Compound	conformer	ΔE (kcal/mol)[a]	Population (%)[b]
	C1	0	49.57
	C2	0.000112	41.74
**1**	C3	0.002962	0.15
	C4	0.002136	2.99
	C5	0.001892	5.55

[a]Relative to conformer C1 with $E_{6\text{-}31+G(d,\ p)}$ = -1083.1236585 Kcal/mol. [b]Calculated using free energy values from Gaussian 03W according to ΔG = -RT In K.

3. The optimized conformers of *R*-**2**

C1	C2
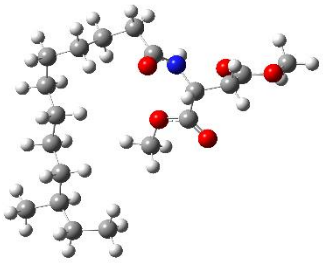	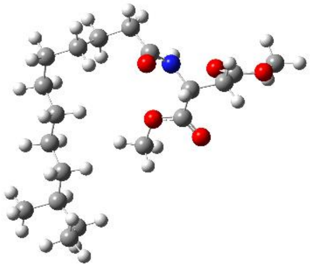
C3	C4
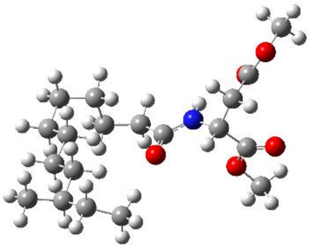	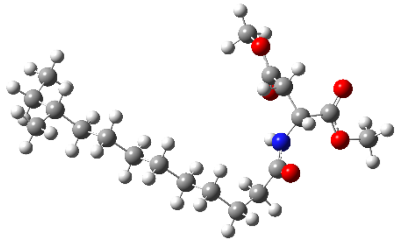
C5	
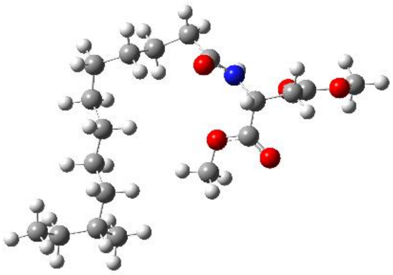	

4. B3LYP-calculated relative energies (Kcal/mol) and conformational population (%) for the most stable conformers of *R*-**2**

Compound	conformer	ΔE (kcal/mol)[a]	Population (%)[b]
**2**	C1	0	65.85
	C2	0.000258	26.79
	C3	0.000268	4.59
	C4	0.001321	1.52

	C5	0.001471	1.25

[a]Relative to conformer C1 with $E_{6\text{-}31+G(d,\ p)}$ = -1021.1352285 Kcal/mol. [b]Calculated using free energy values from Gaussian 03W according to ΔG = -RT In K.

3.5.5. The optimized conformers of *R*-**3**

C1	C2
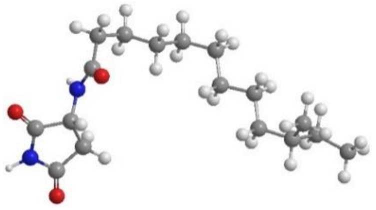	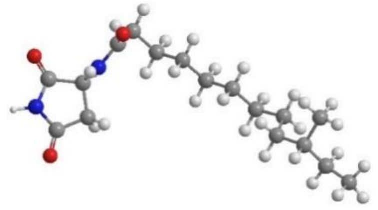
C3	C4
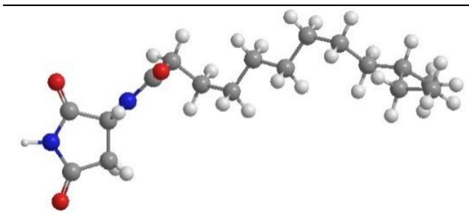	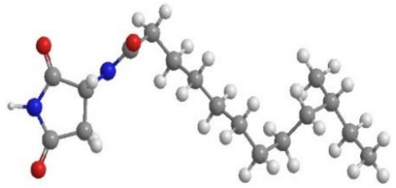
C5	
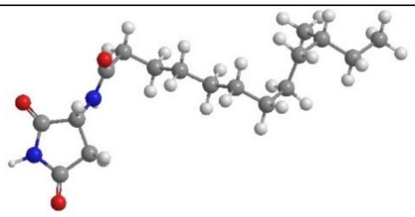	

3.5.6. B3LYP-calculated relative energies (Kcal/mol) and conformational population (%) for the most stable conformers of *R*-**3**

Compound	conformer	ΔE (kcal/mol)[a]	Population (%)[b]
	C1	0	33.66
	C2	0.000352	25.62
**3**	C3	0.001761	25.34
	C4	0.002321	8.30
	C5	0.002981	7.07

[a]Relative to conformer C1 with $E_{6\text{-}31+G(d,\ p)}$ = -1001.2235296 Kcal/mol. [b]Calculated using free energy values from Gaussian 03W according to ΔG = -RT In K.

3.5.7. The optimized conformers of *R*-**4**

C1	C2
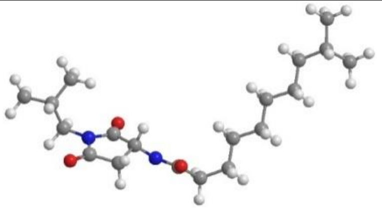	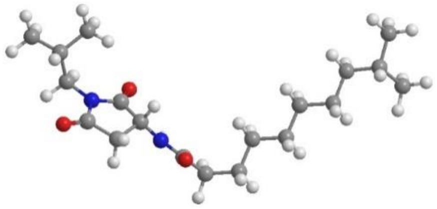
C3	C4
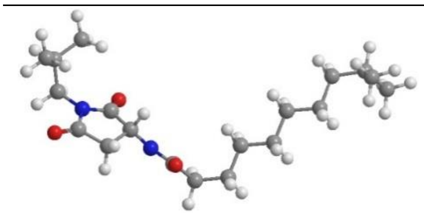	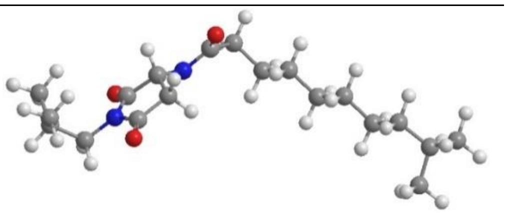

3.5.8. B3LYP-calculated relative energies (Kcal/mol) and conformational population (%) for the most stable conformers of *R*-**4**.

Compound	conformer	ΔE (kcal/mol)	Population (%)
**4**	C1	0	68.92
	C2	0.000372	29.91
	C3	0.003496	1.20
	C4	0.003701	0.97

[a]Relative to conformer C1 with E6-31+G(d) = -1080.07154857 Kcal/mol. [b]Calculated using free energy values from Gaussian 03W according to ΔG = -RT In K.

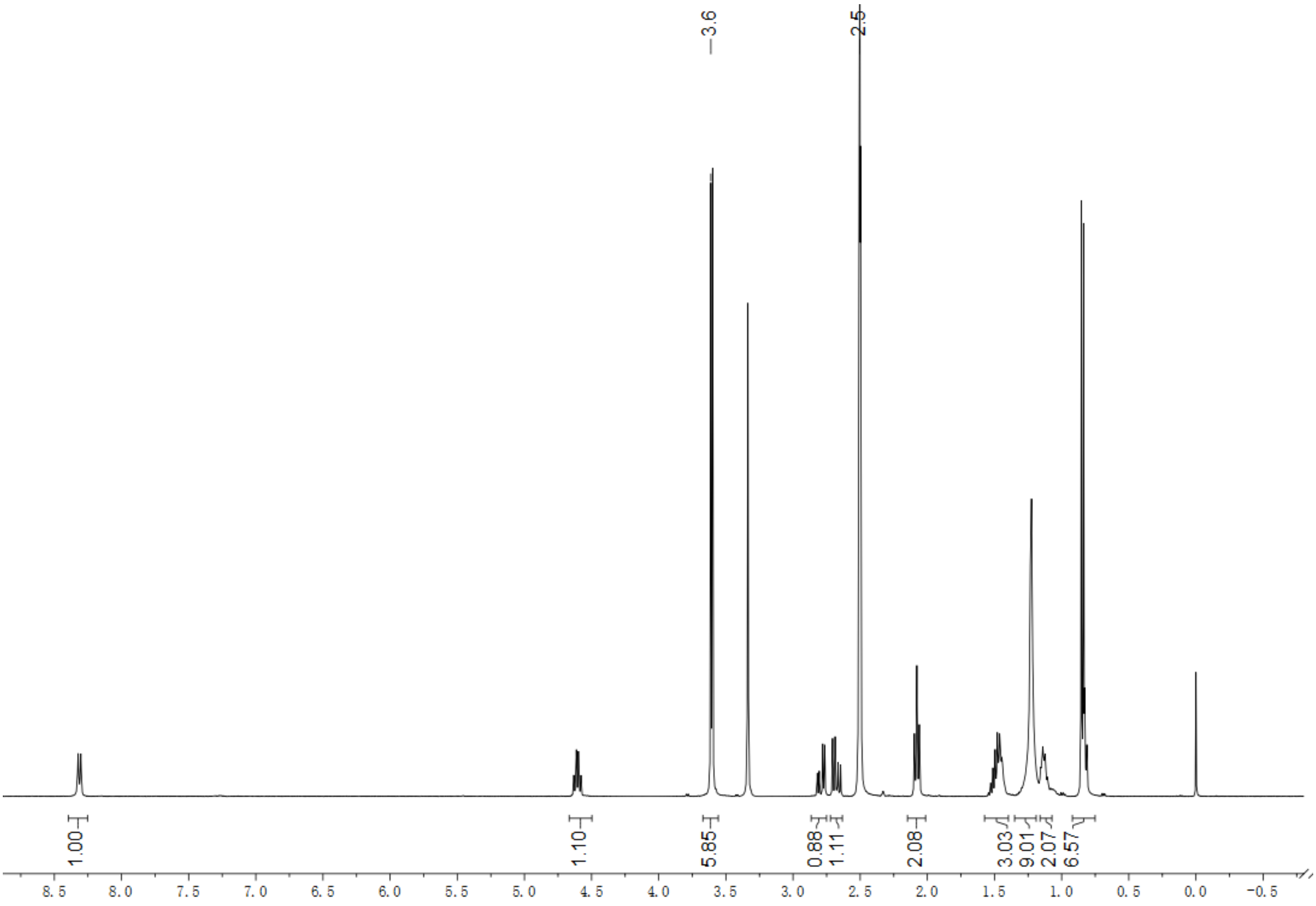

Figure S1. ^{1}H-NMR spectrum of compound **1** (400 MHz, DMSO-d_6)

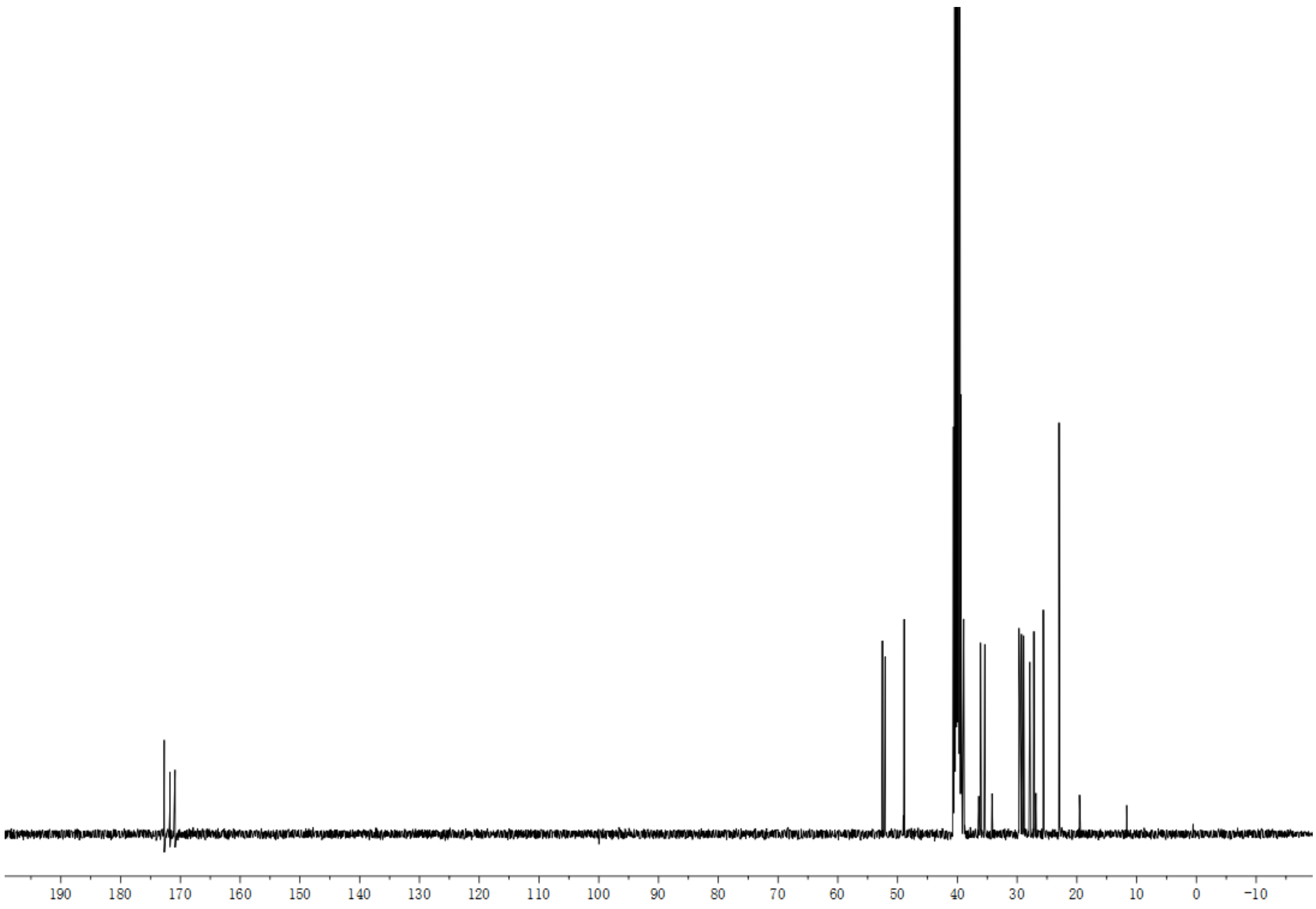

Figure S2. ^{13}C-NMR spectrum of compound **1** (100 MHz, DMSO-d_6)

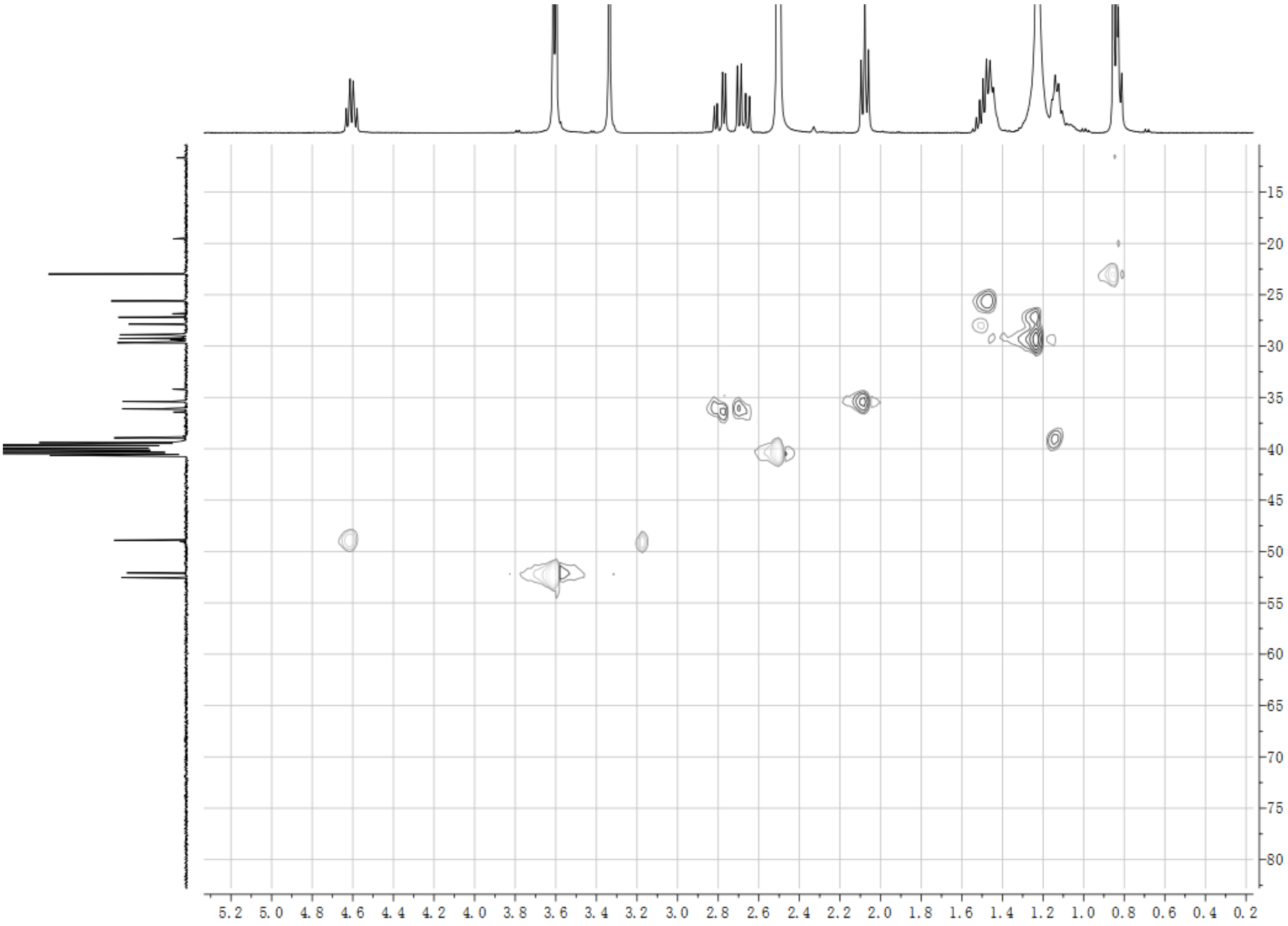

Figure S3. HSQC spectrum of compound **1** (400 MHz, DMSO-d_6)

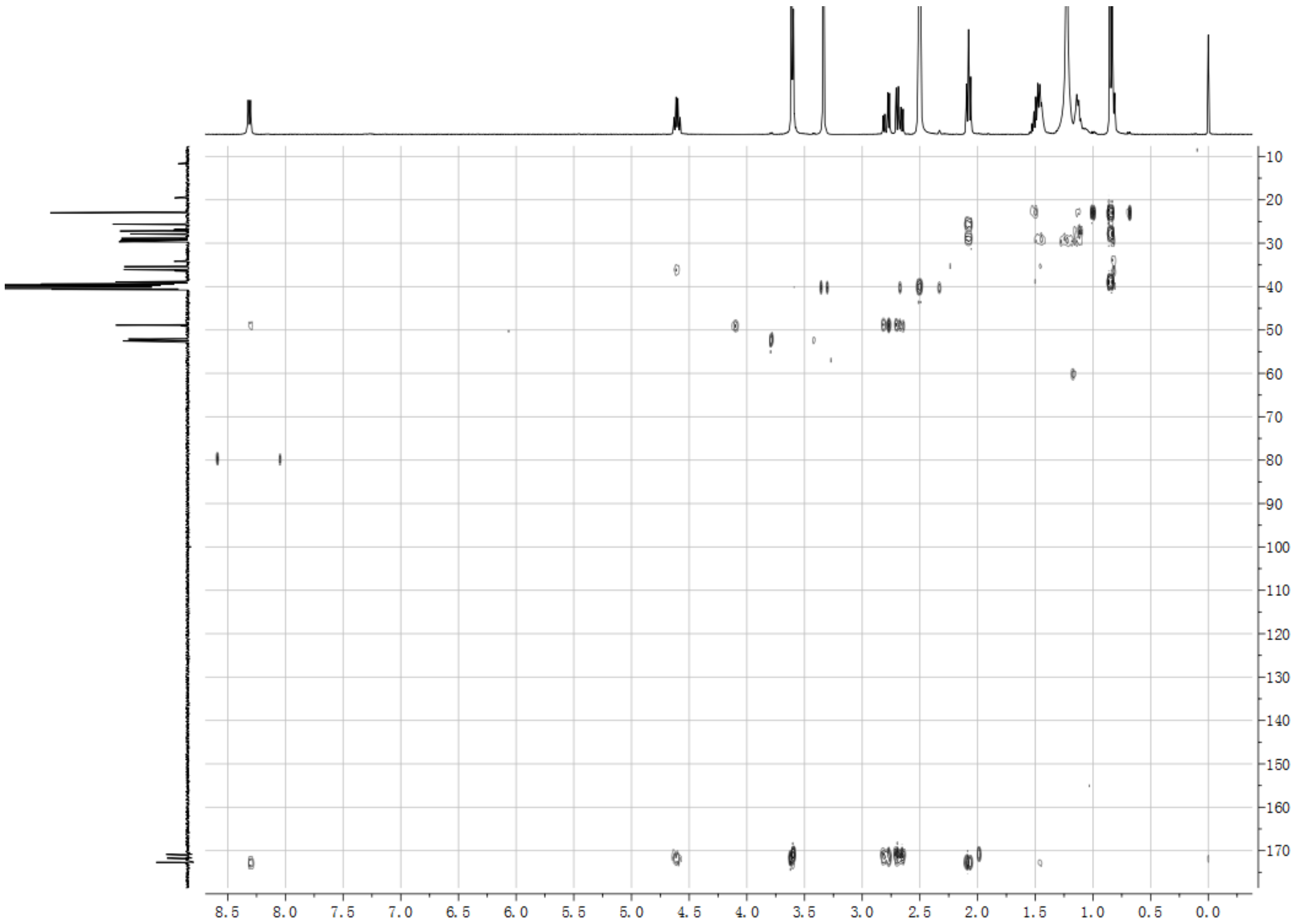

Figure S4. HMBC spectrum of compound **1** (400 MHz, DMSO-d_6)

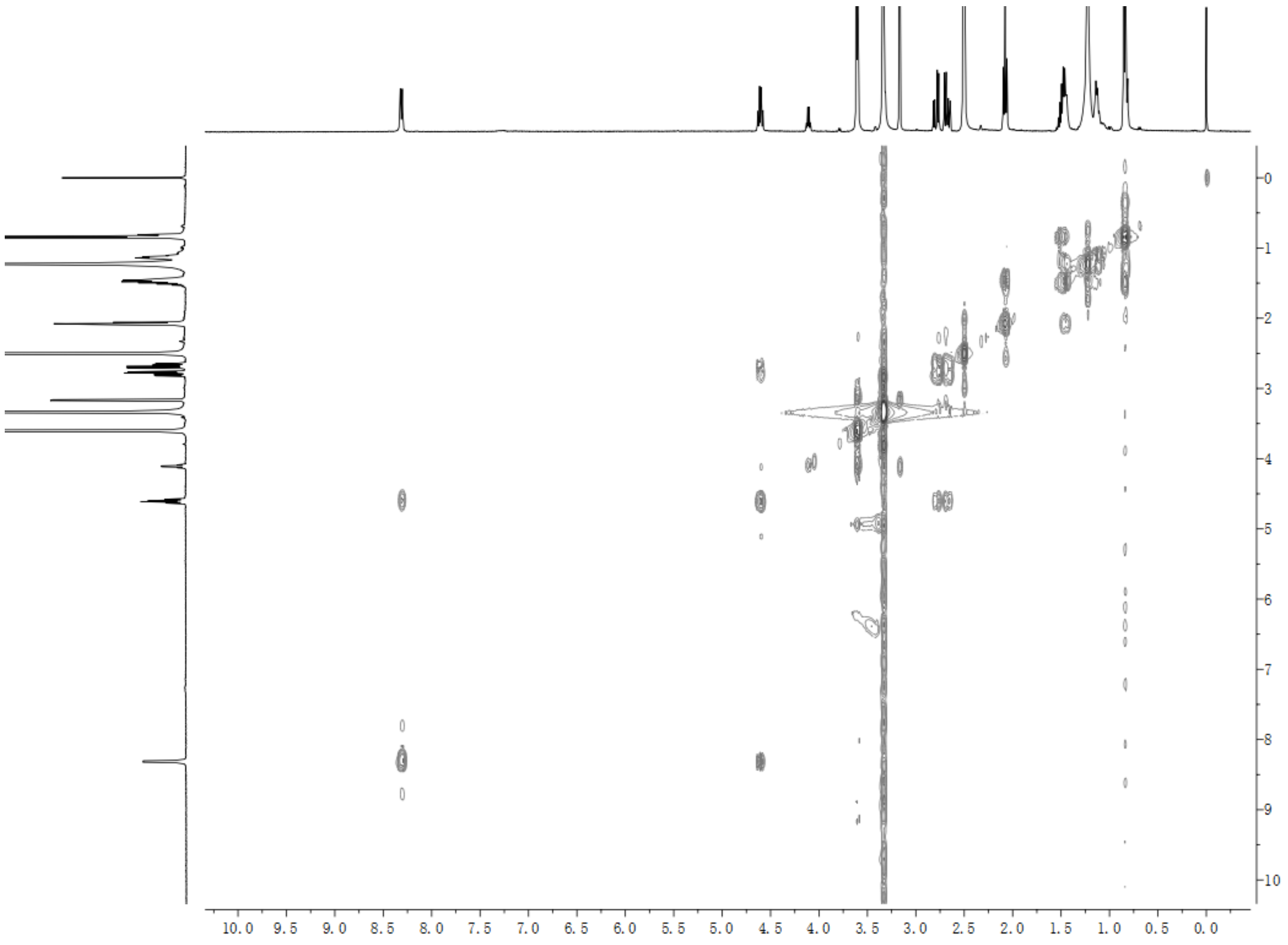

Figure S5. COSY spectrum of compound **1** (400 MHz, DMSO-d_6)

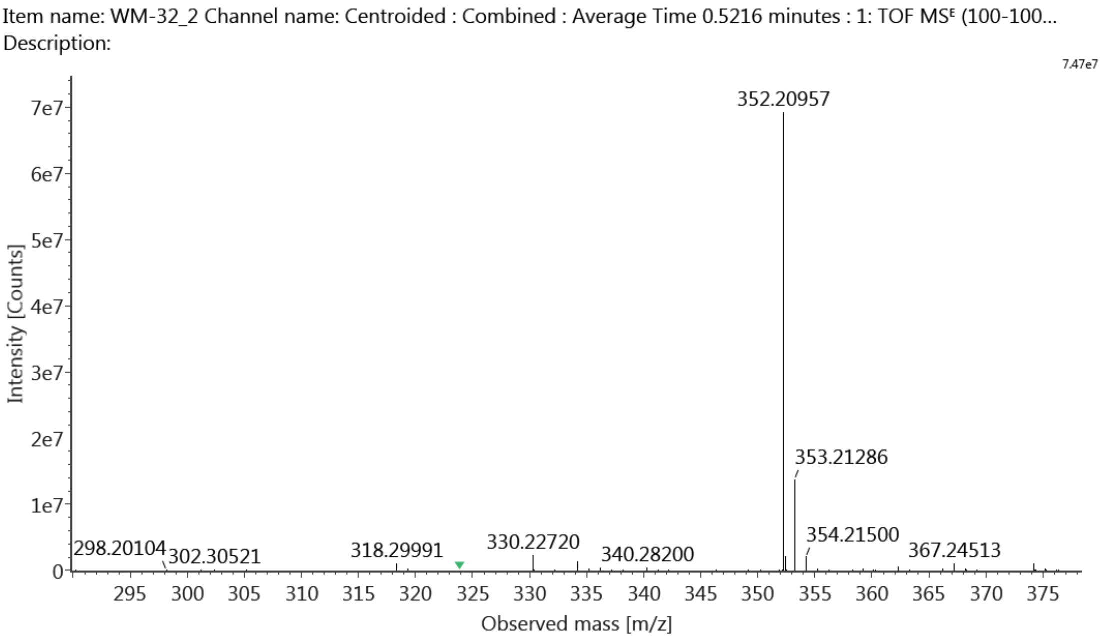

Formula	Calculated Mass	Calculated Mz	Mz	m/z error (mDa)	m/z error (PPM)
$C_{17}H_{31}NO_5$	329.2202	352.2100	352.2096	-0.3	-0.9

Figure S6. HR-ESI-MS spectrum of compound **1**

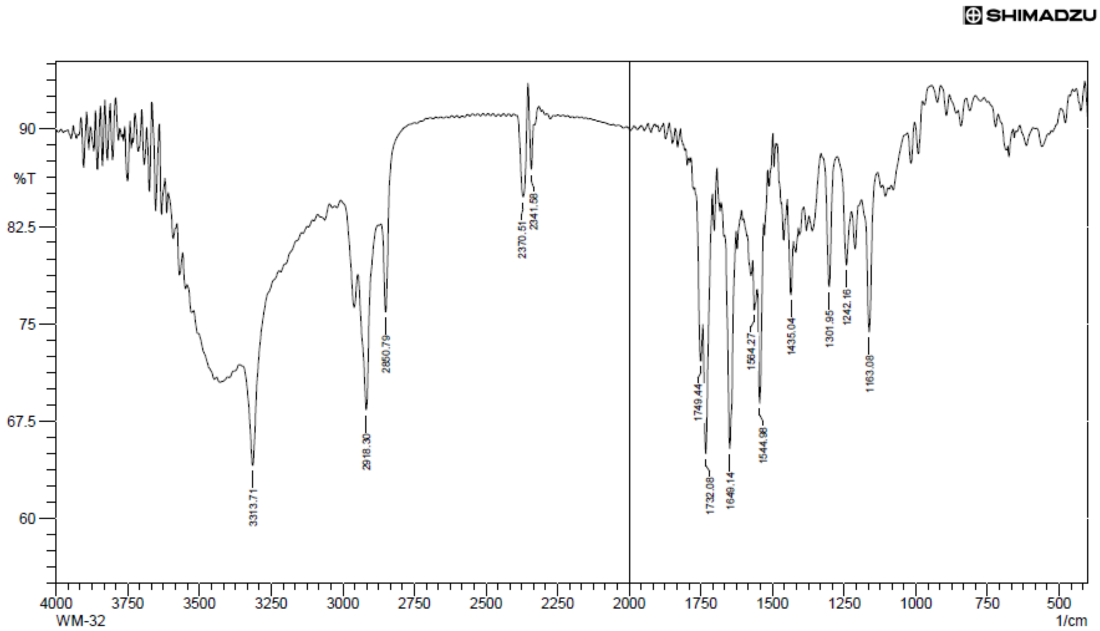

Figure S7. IR spectrum of compound **1**

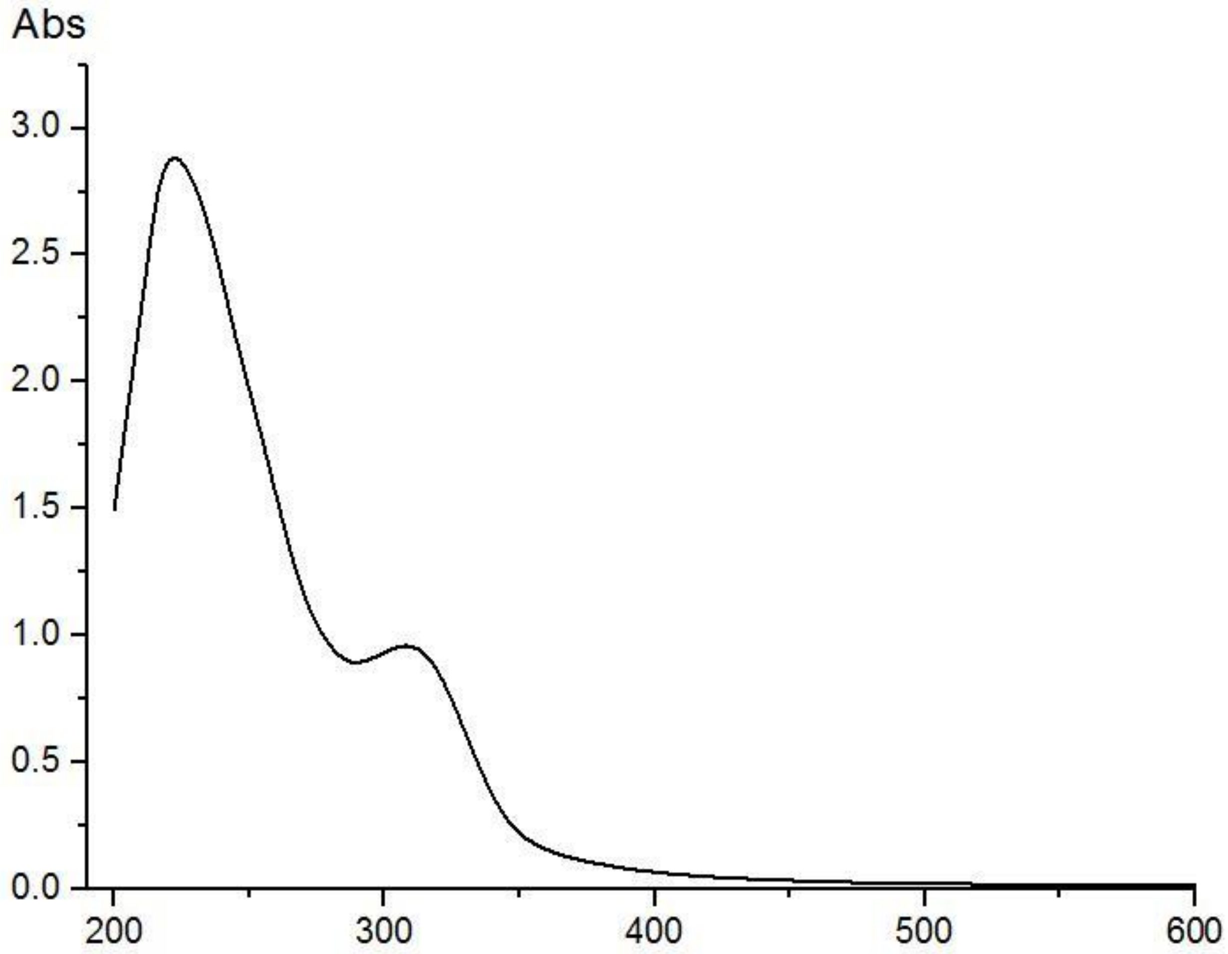

Figure S8. UV spectrum of compound **1**

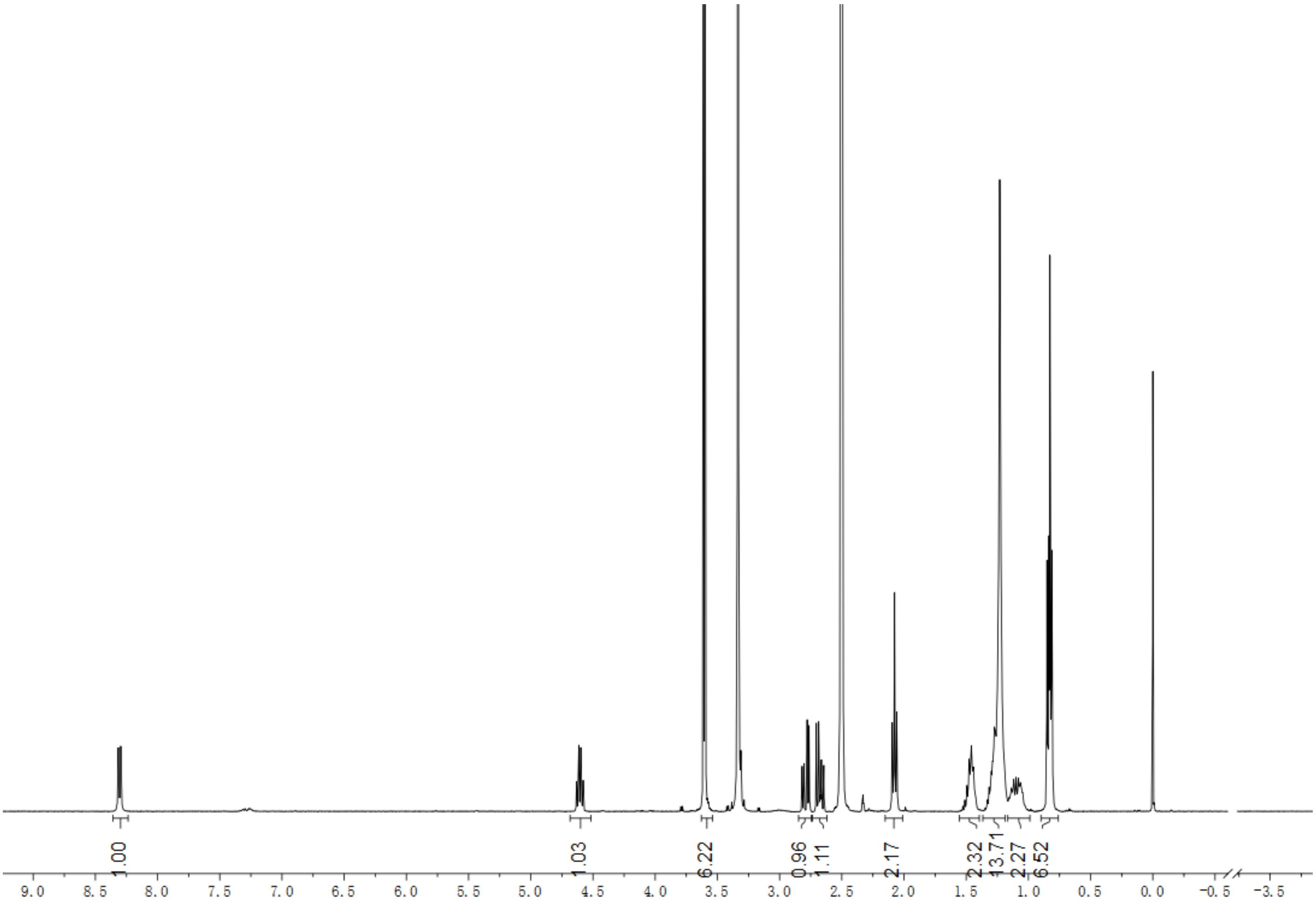

Figure S9. ^{1}H-NMR spectrum of compound **2** (400 MHz, DMSO-d_6)

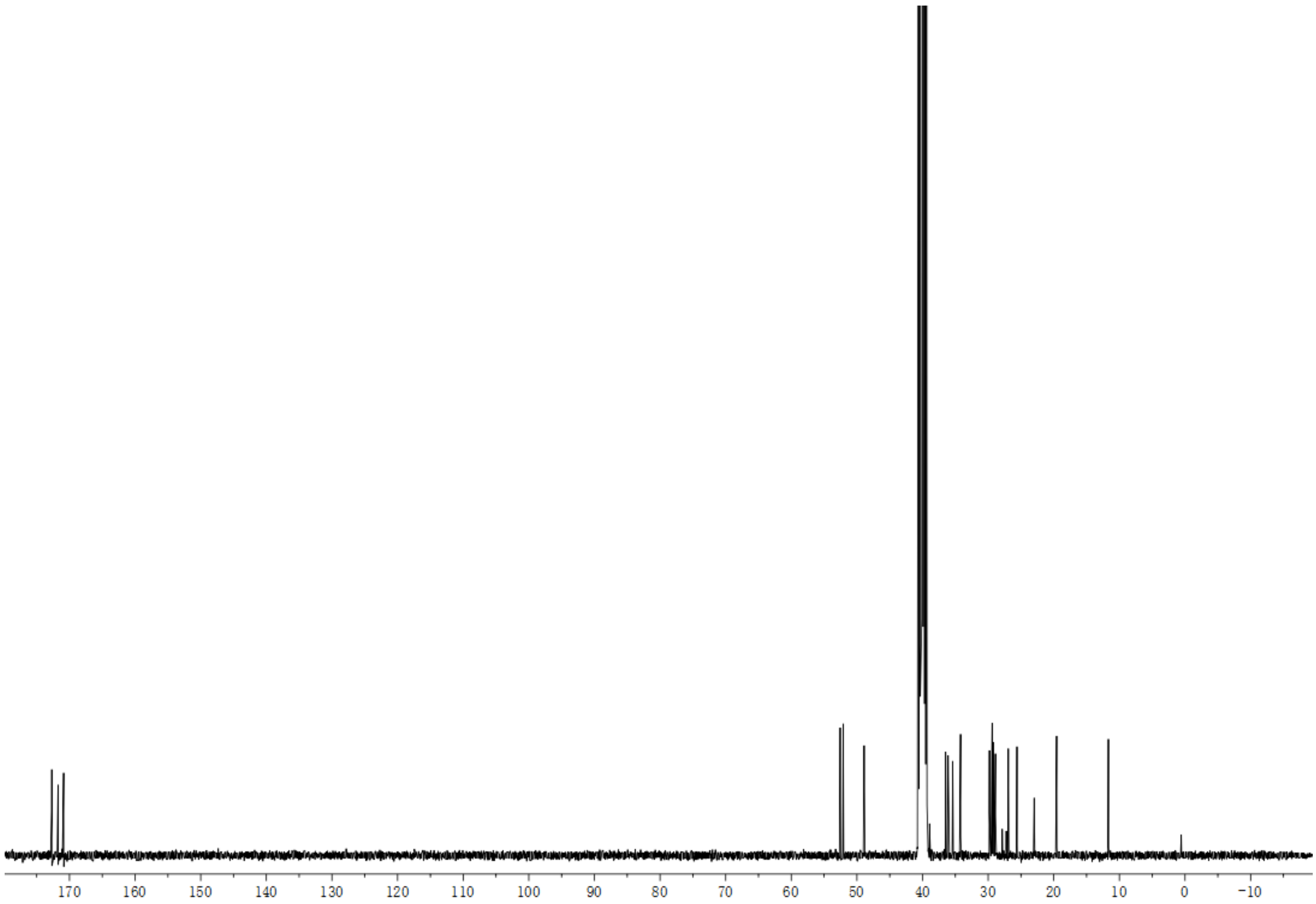

Figure S10. ^{13}C-NMR spectrum of compound **2** (100 MHz, DMSO-d_6)

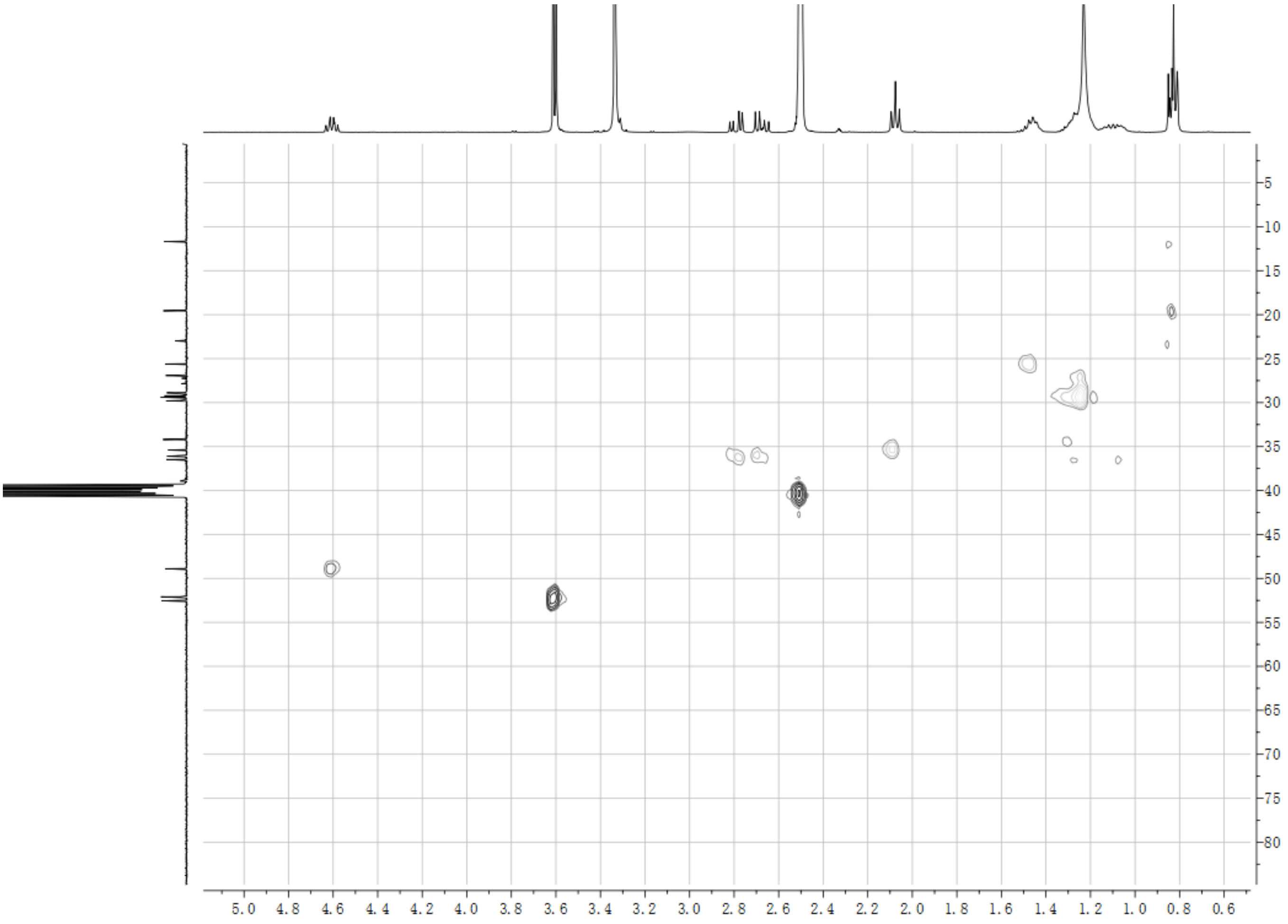

Figure S11. HSQC spectrum of compound **2** (400 MHz, DMSO-d_6)

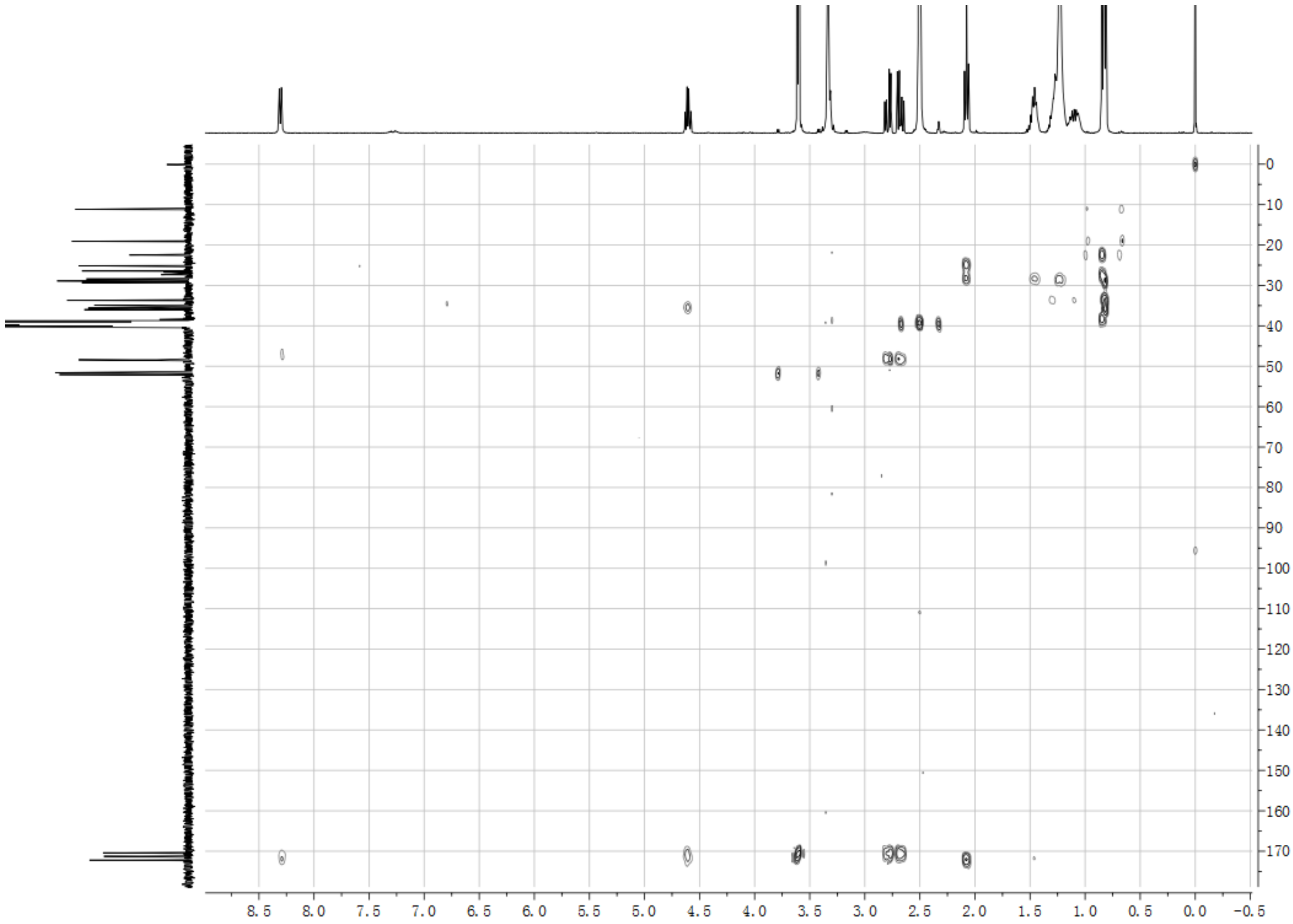

Figure S12. HMBC spectrum of compound **2** (400 MHz, DMSO-d_6)

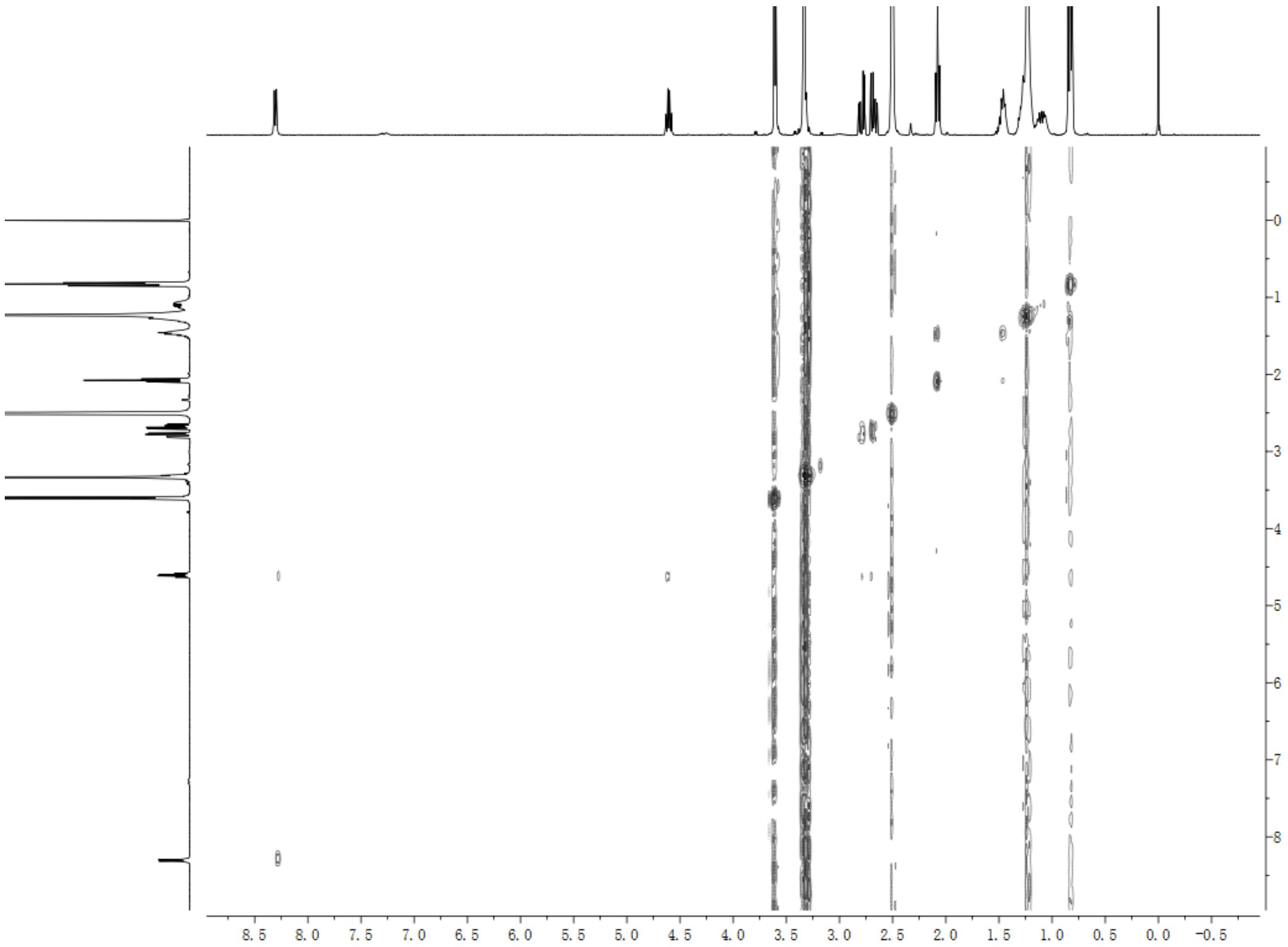

Figure S13. COSY spectrum of compound **2** (400 MHz, DMSO-d_6)

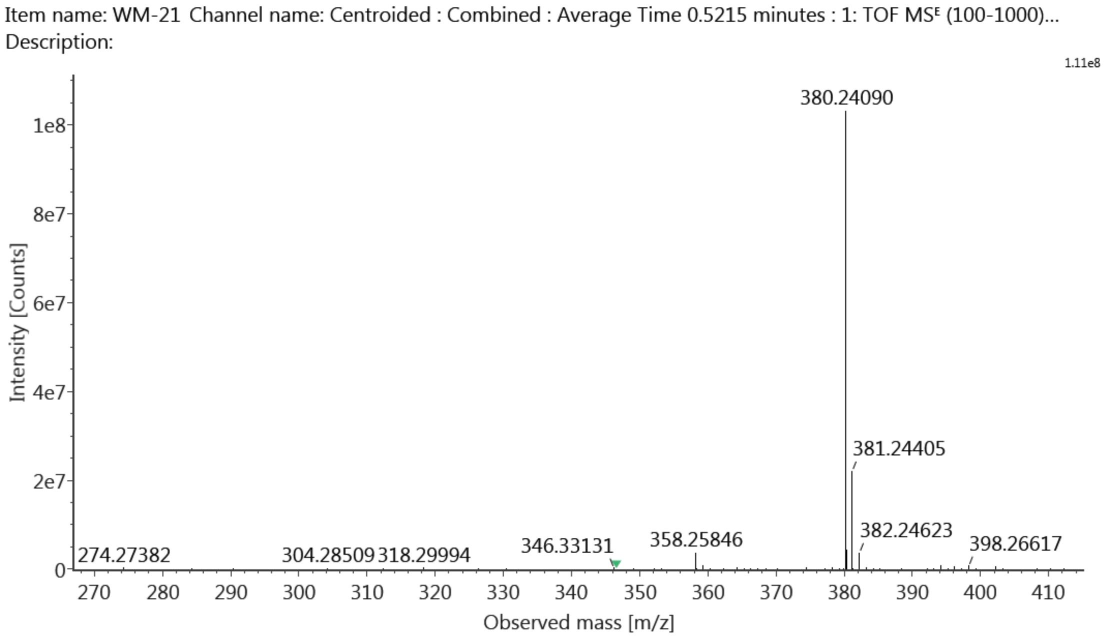

Formula	Calculated Mass	Calculated Mz	Mz	m/z error (mDa)	m/z error (PPM)
$C_{19}H_{35}NO_5$	357.2515	380.2413	380.2409	-0.3	-0.9

Figure S14. HR-ESI-MS spectrum of compound **2**

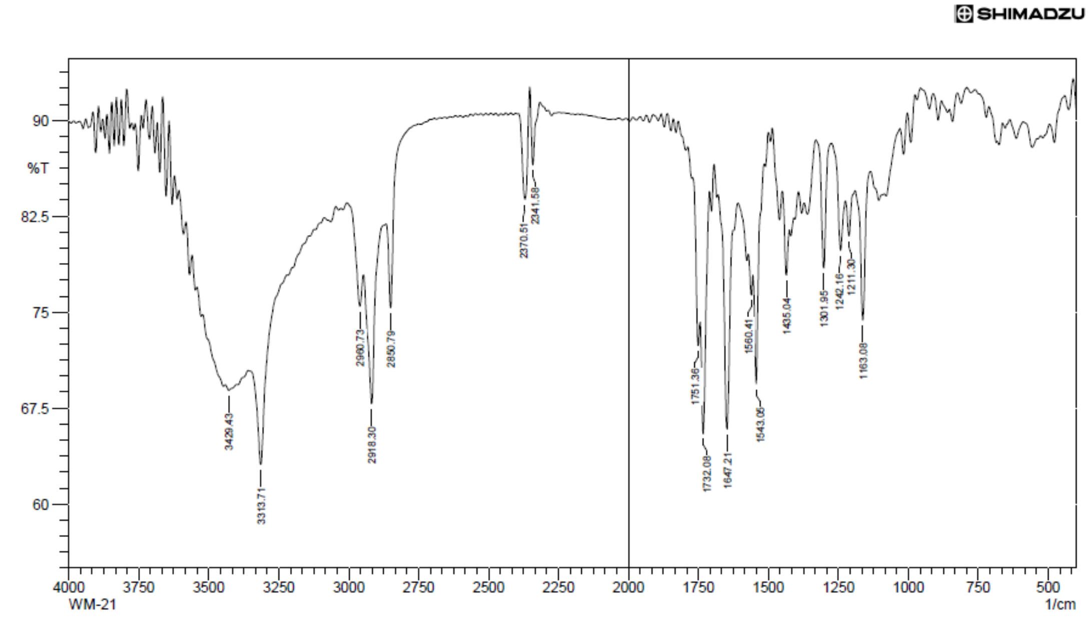

Figure S15. IR spectrum of compound **2**

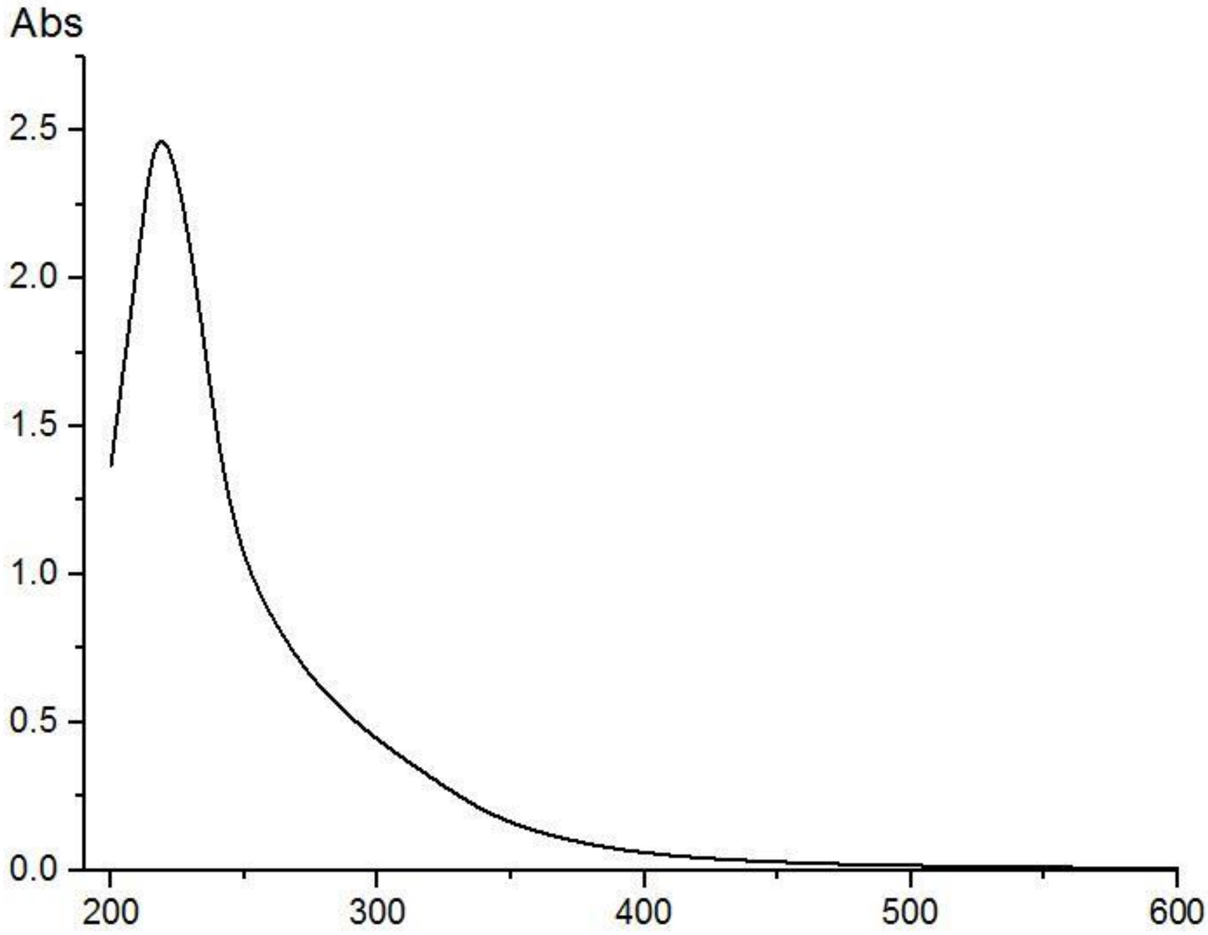

Figure S16. UV spectrum of compound **2**

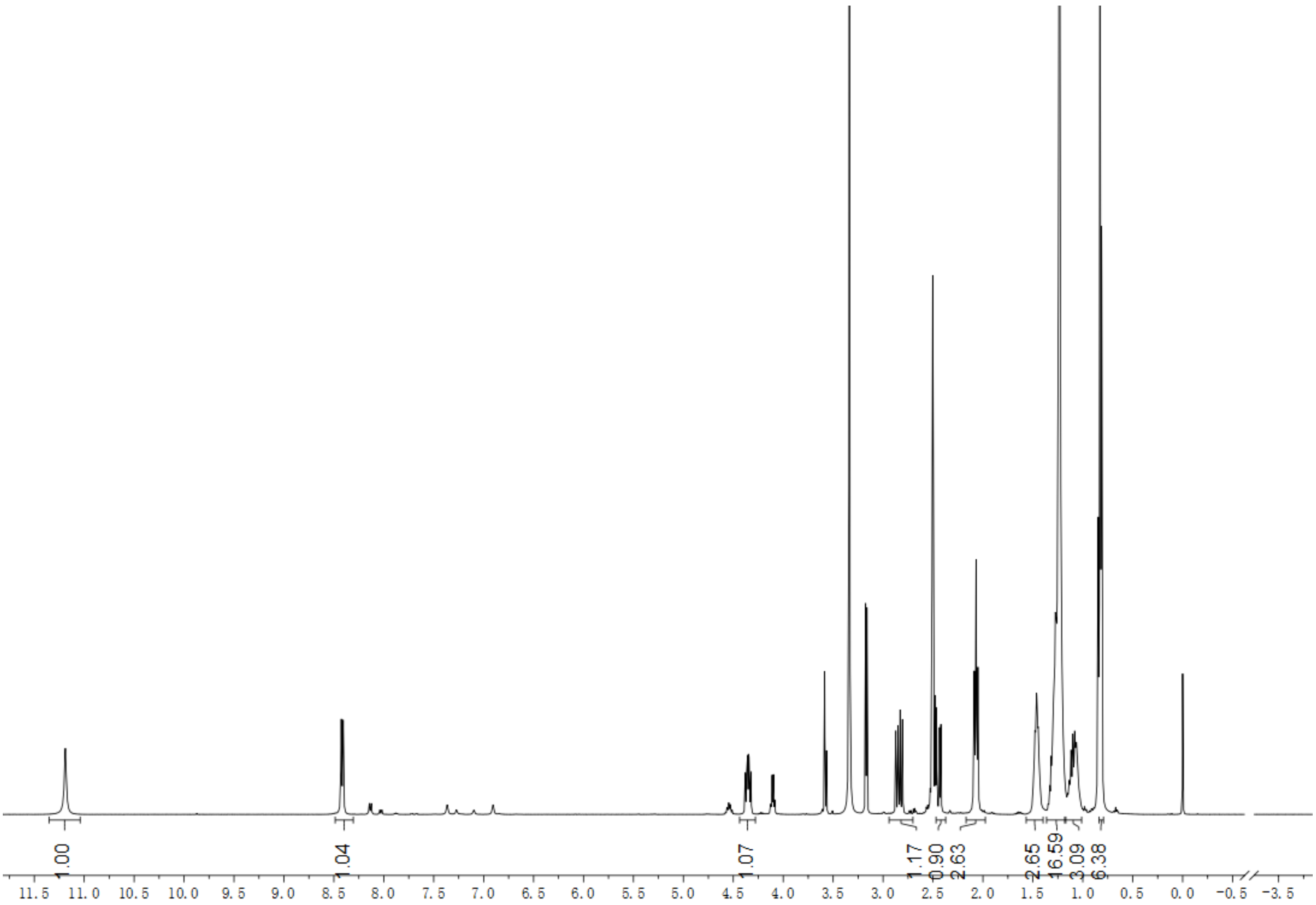

Figure S17. ^{1}H-NMR spectrum of compound **3** (400 MHz, DMSO-d_6)

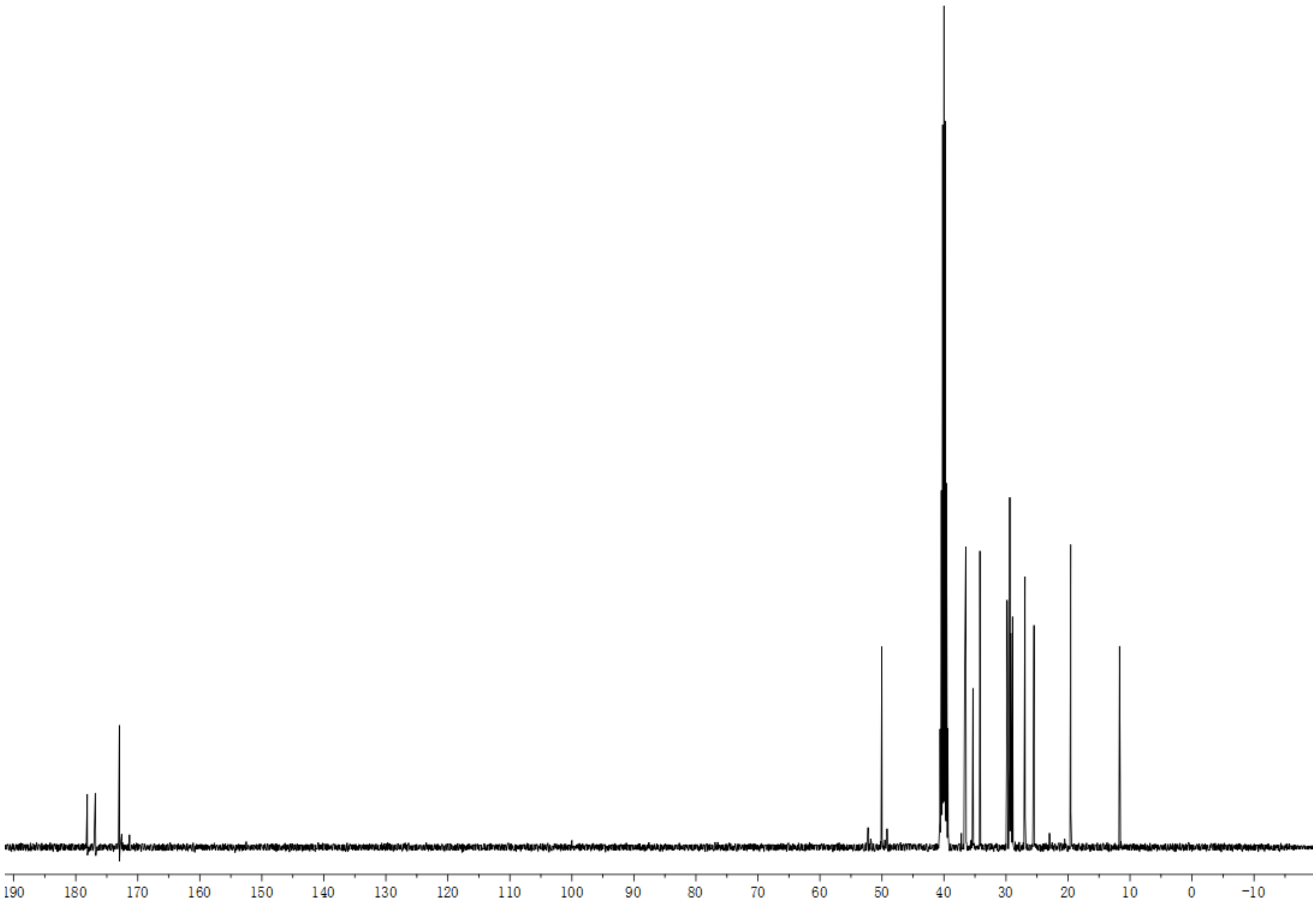

Figure S18. ^{13}C-NMR spectrum of compound **3** (100 MHz, DMSO-d_6)

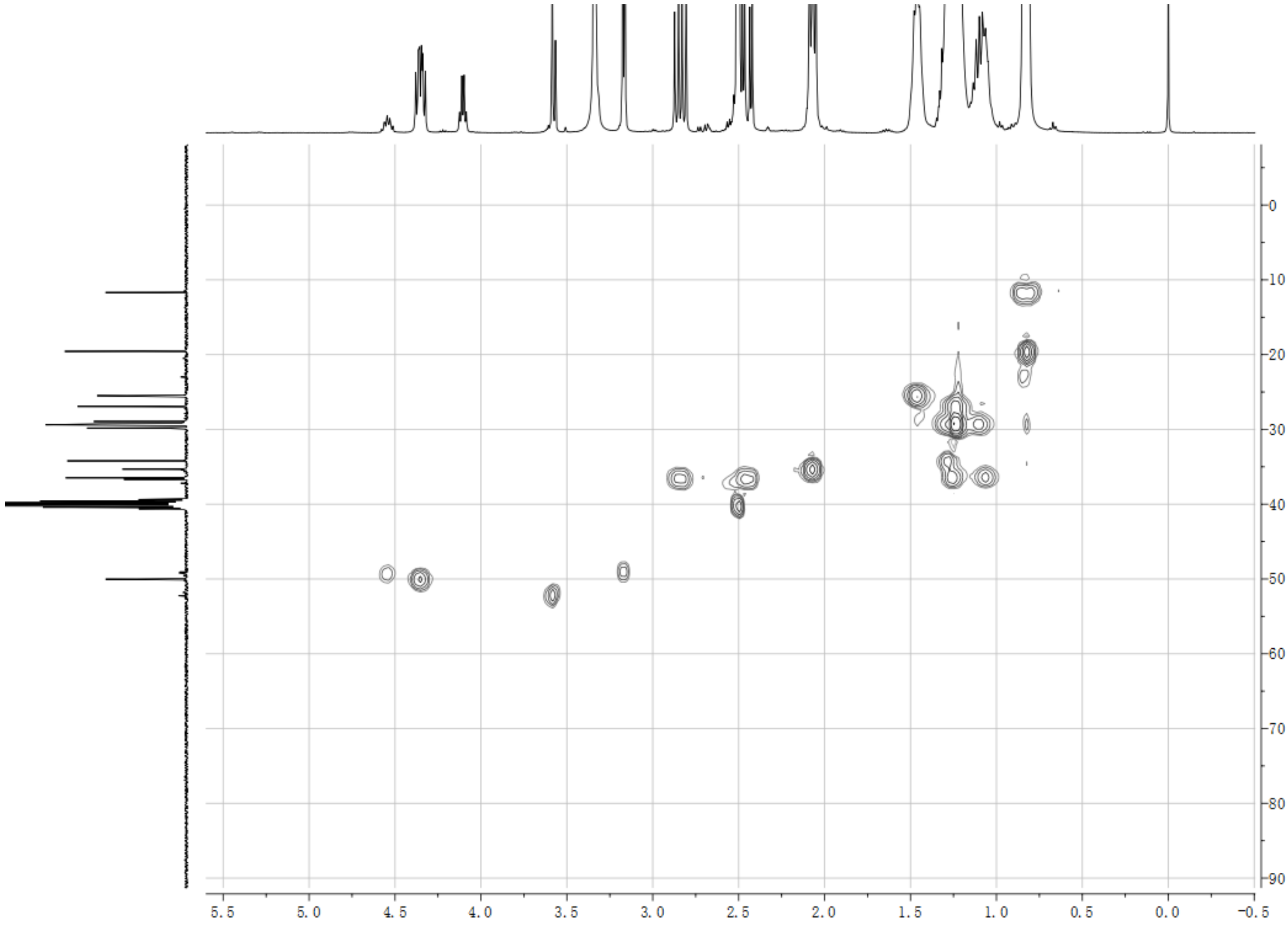

Figure S19. HSQC spectrum of compound **3** (400 MHz, DMSO-d_6)

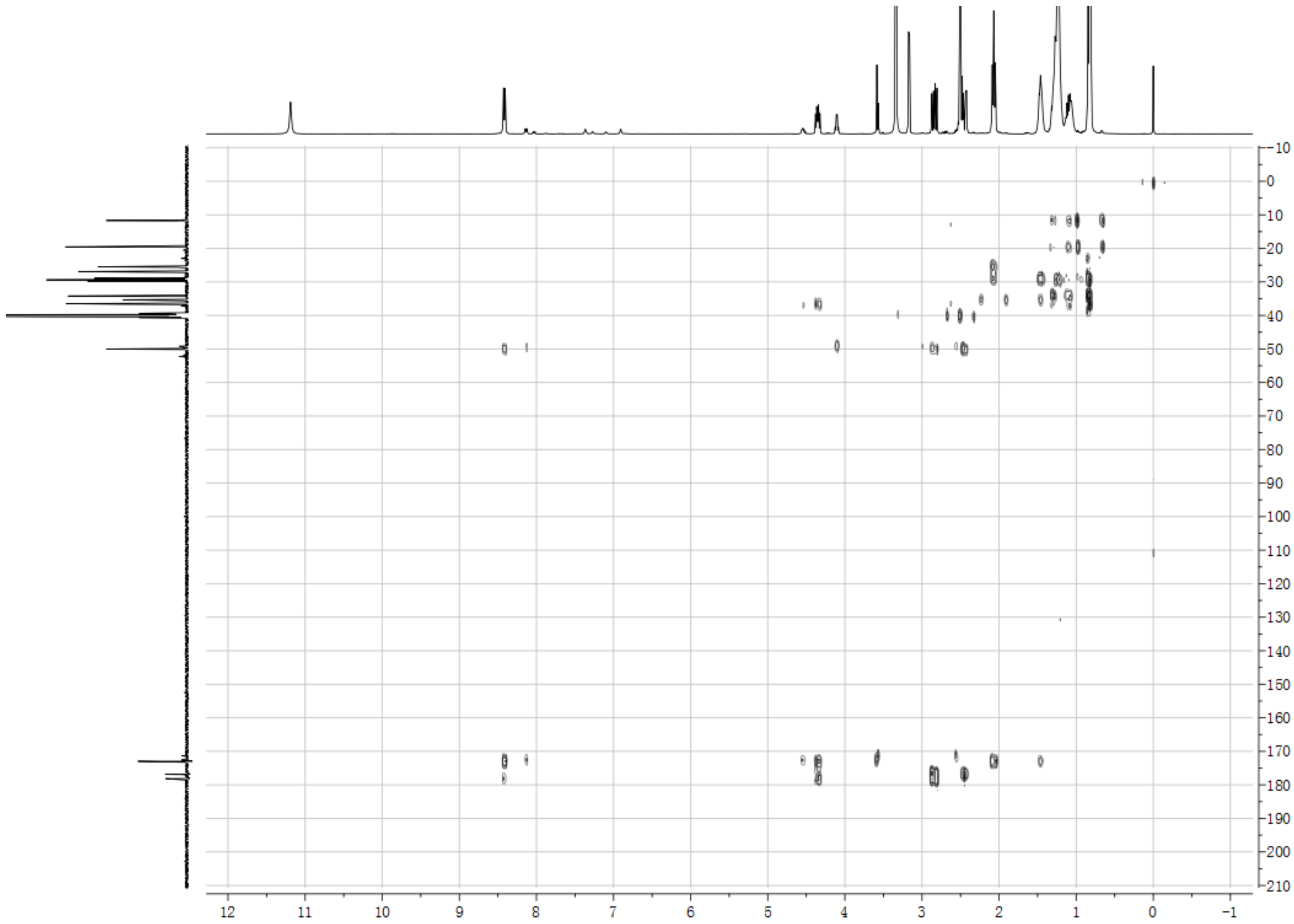

Figure S20. HMBC spectrum of compound **3** (400 MHz, DMSO-d_6)

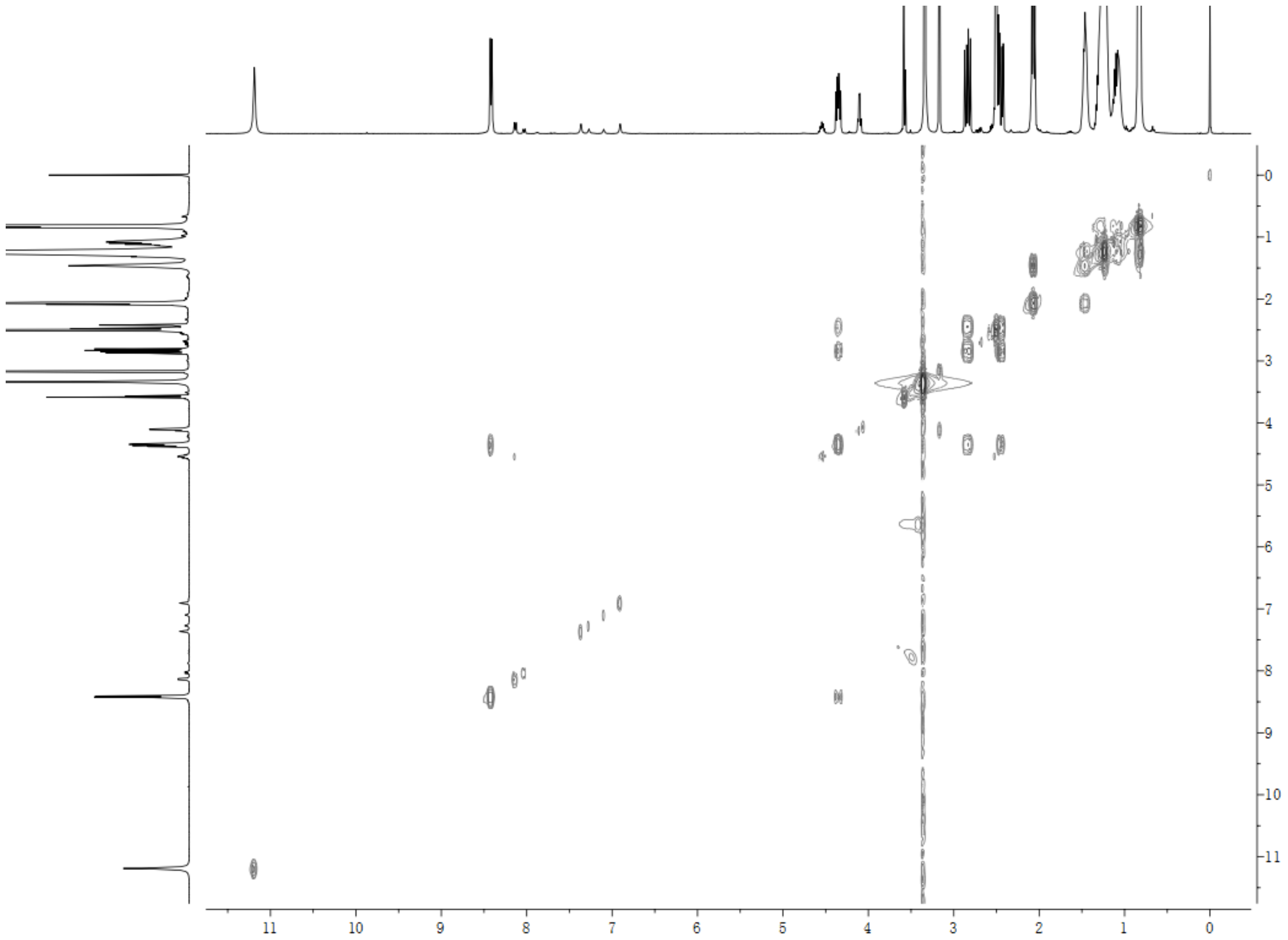

Figure S21. COSY spectrum of compound **3** (400 MHz, DMSO-d_6)

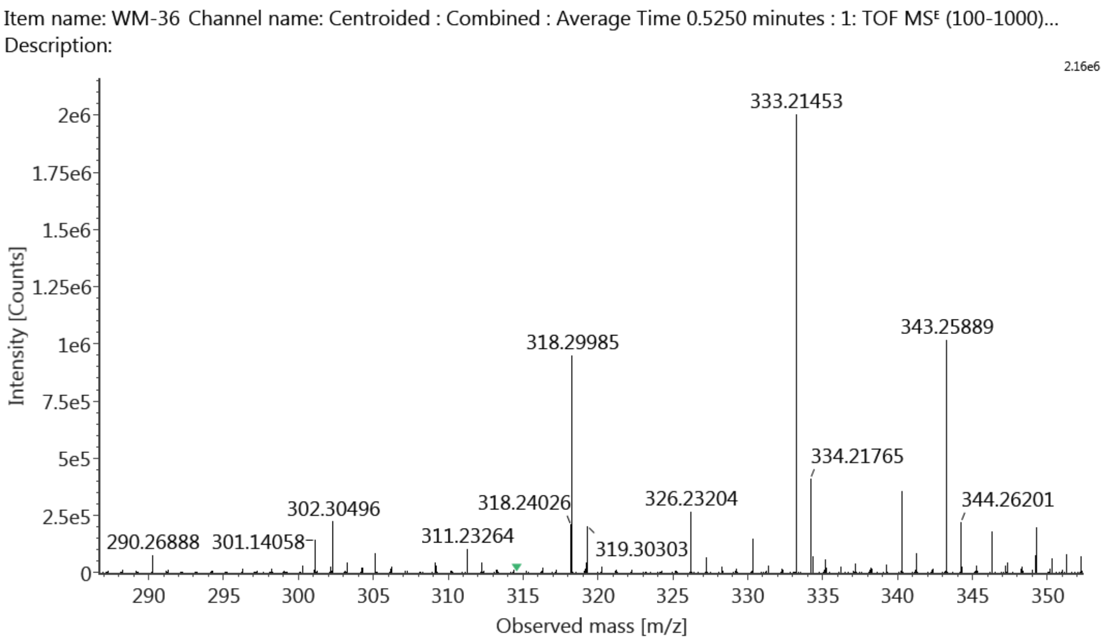

Formula	Calculated Mass	Calculated Mz	Mz	m/z error (mDa)	m/z error (PPM)
$C_{17}H_{30}N_2O_3$	310.2256	333.2154	333.2145	-0.3	-0.9

Figure S22. HR-ESI-MS spectrum of compound **3**

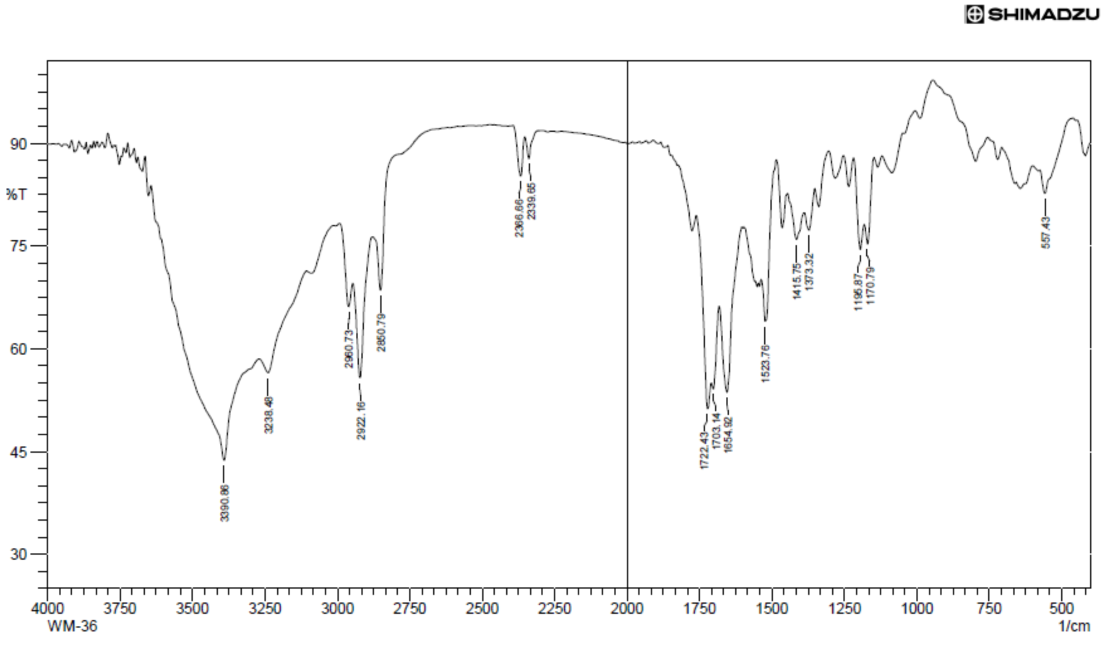

Figure S23. IR spectrum of compound **3**

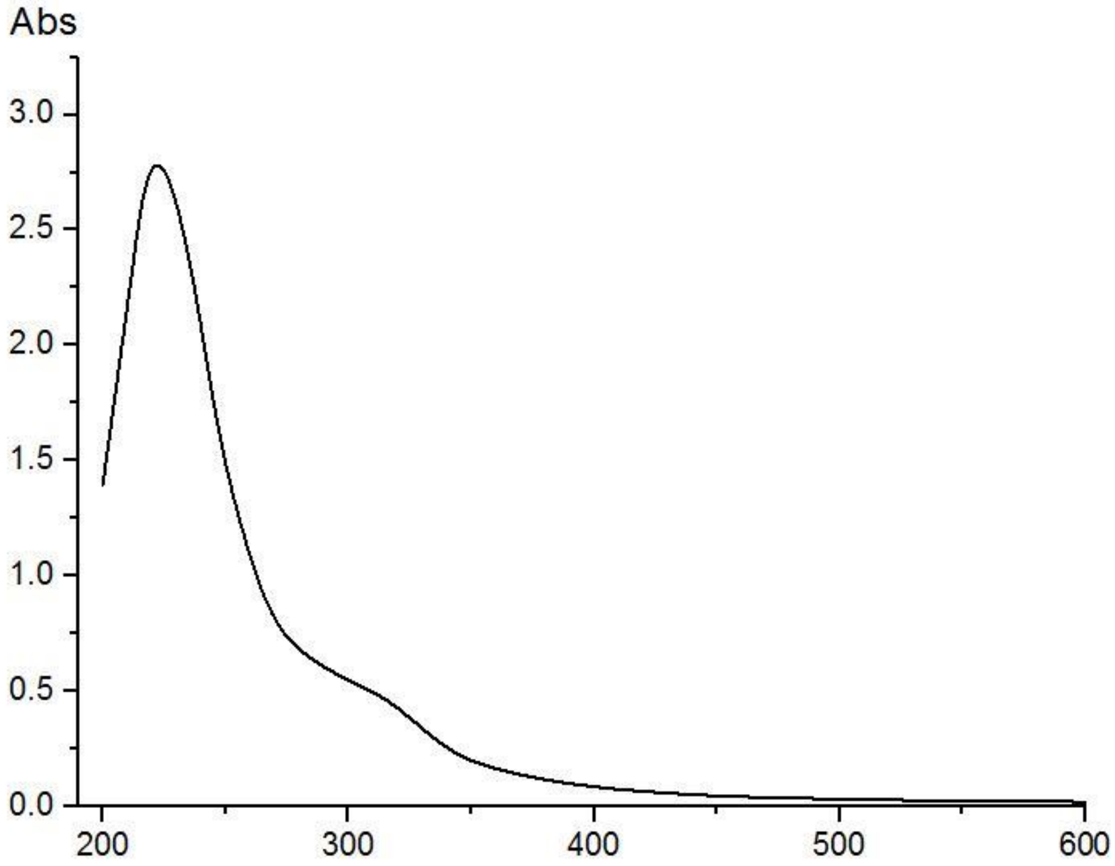

Figure S24. UV spectrum of compound **3**

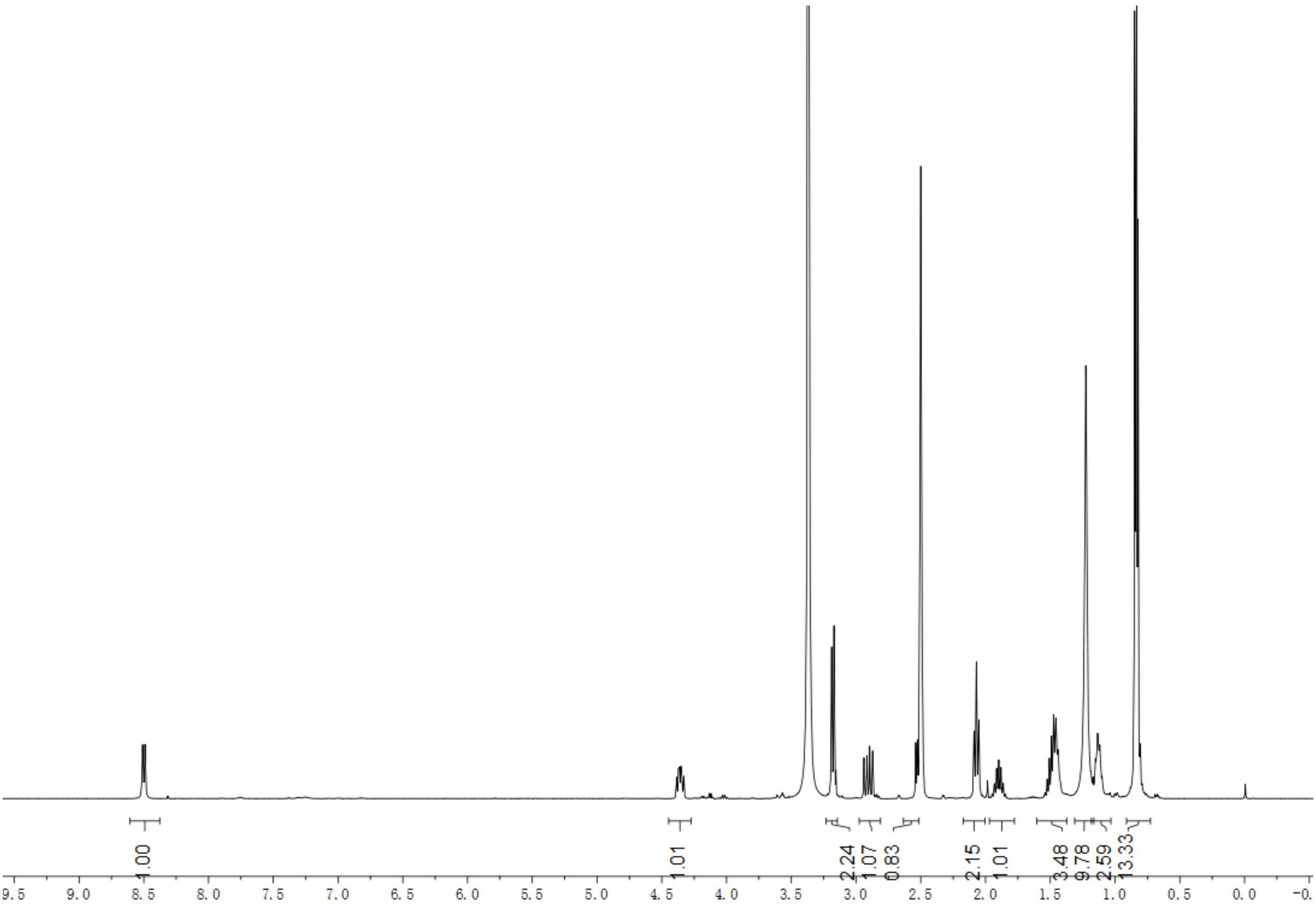

Figure S25. ^{1}H-NMR spectrum of compound **4** (400 MHz, DMSO-*d*$_6$)

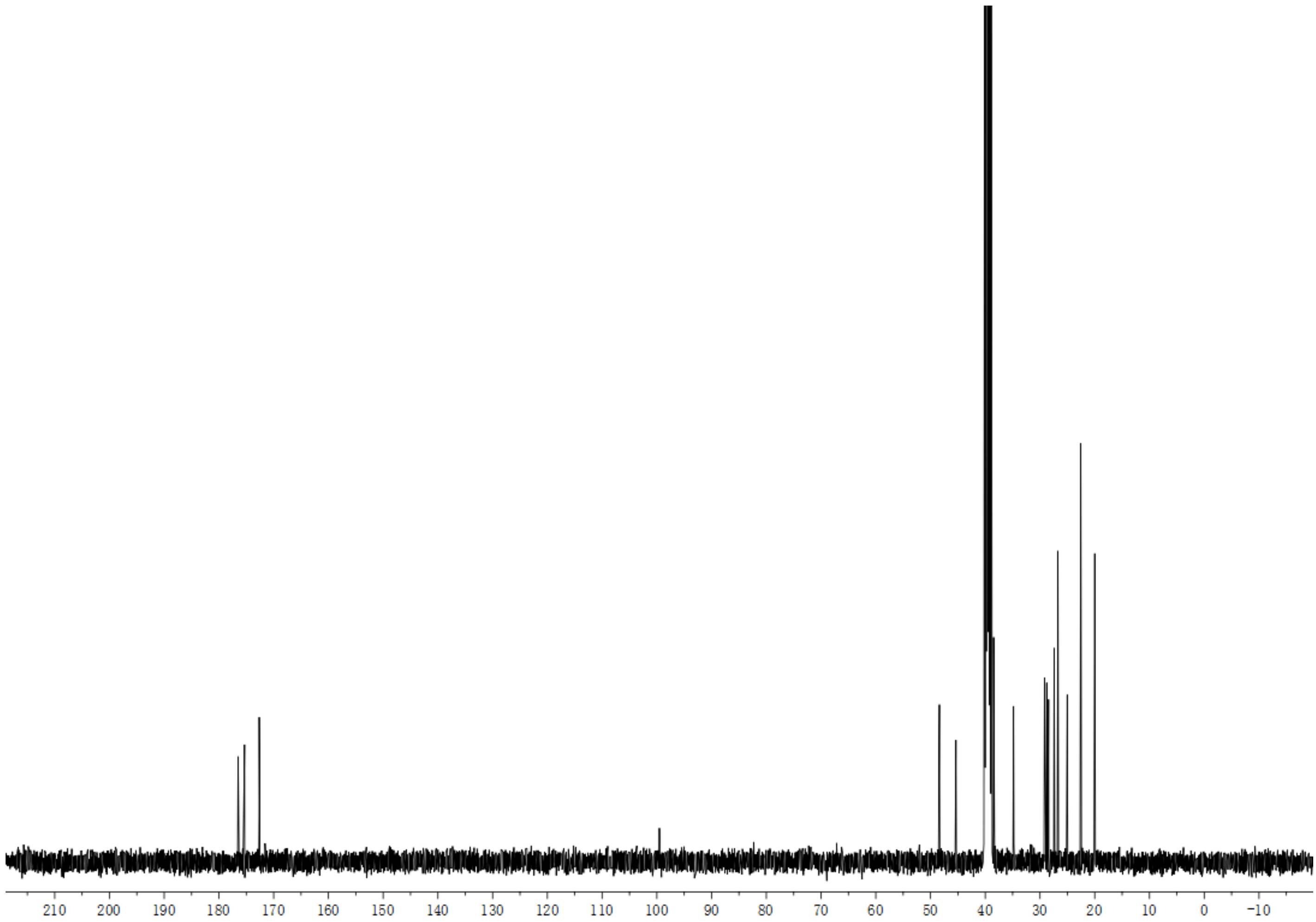

Figure S26. ^{13}C-NMR spectrum of compound **4** (100 MHz, DMSO-d_6)

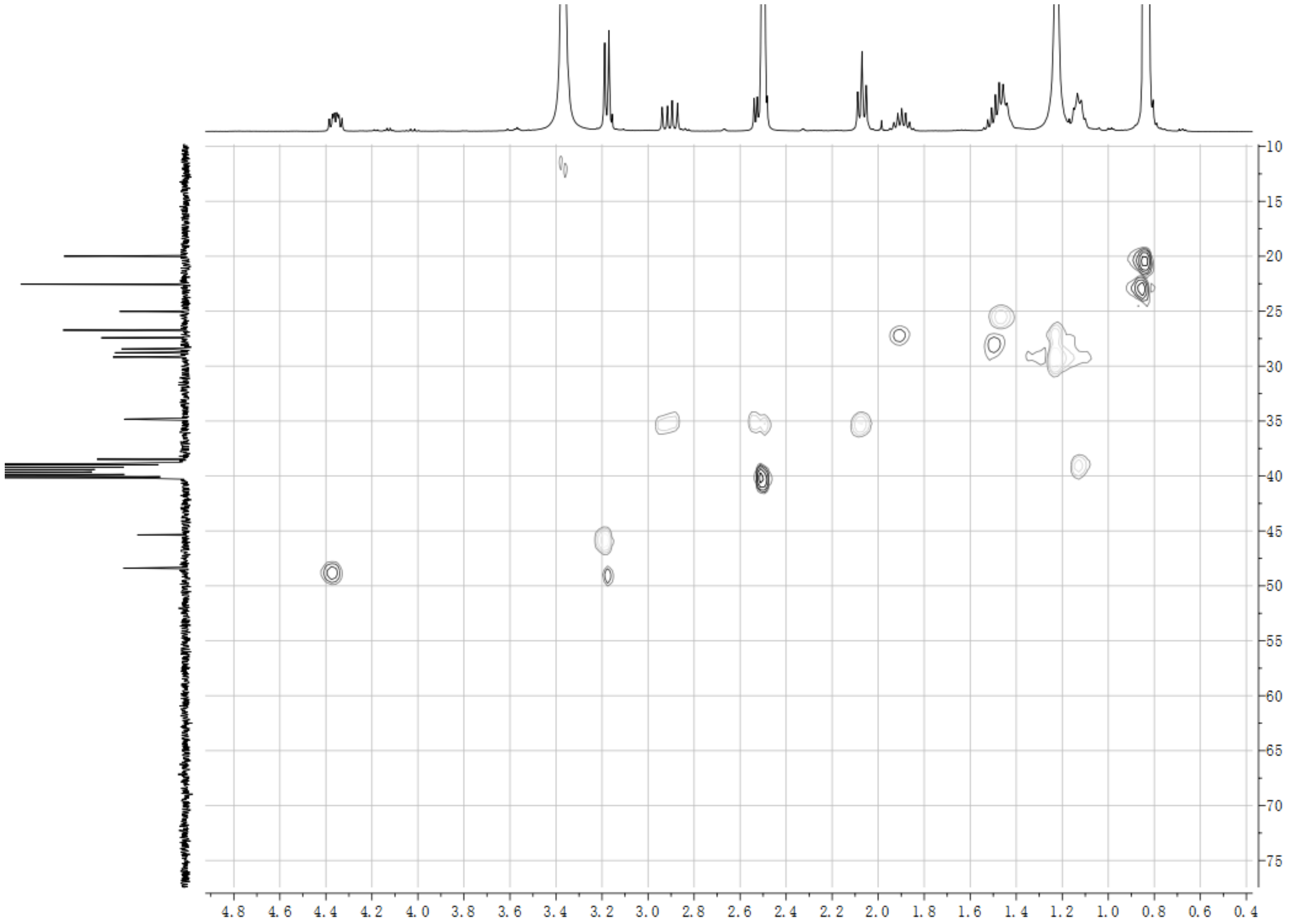

Figure S27.HSQC spectrum of compound **4** (400 MHz, DMSO-d_6)

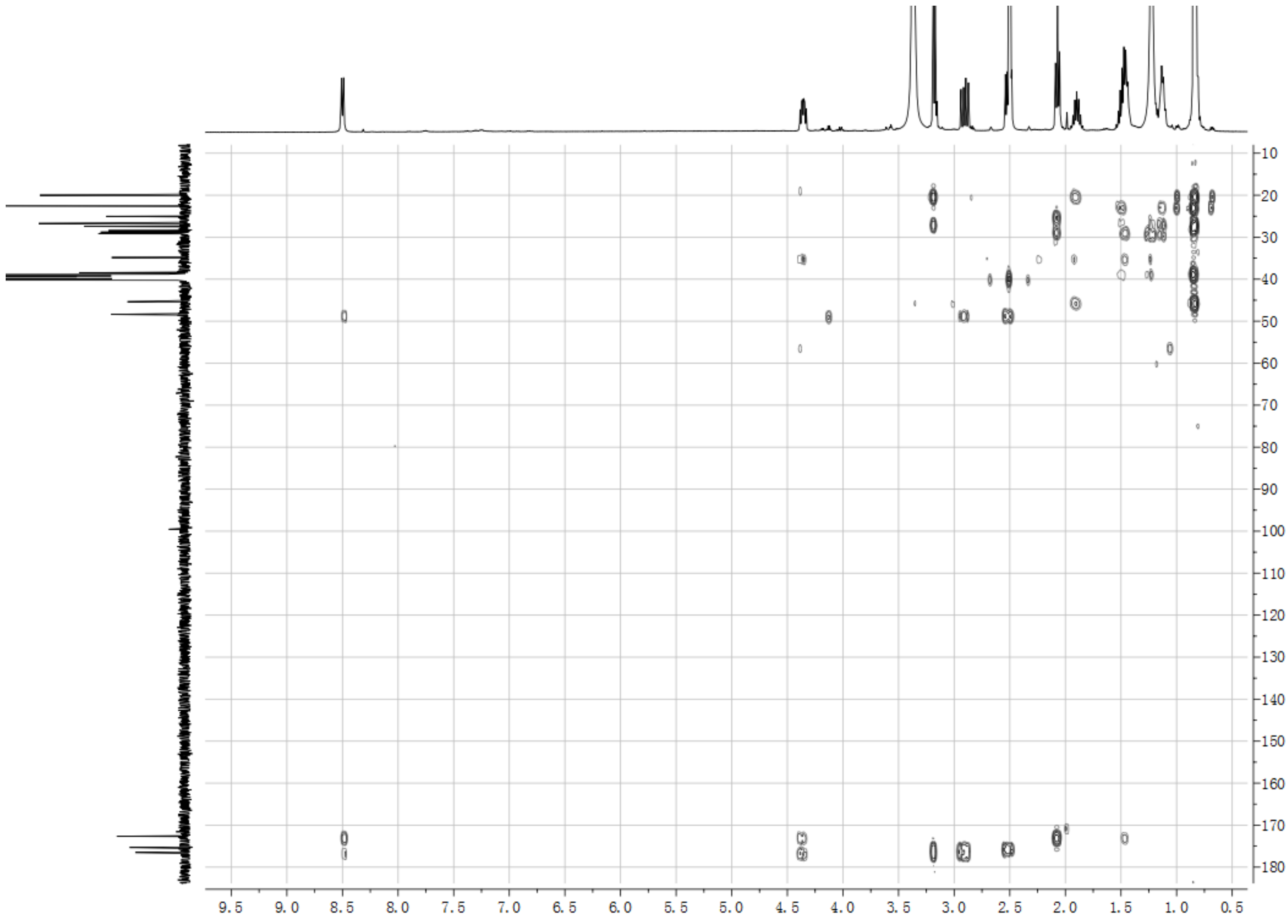

Figure S28. HMBC spectrum of compound **4** (400 MHz, DMSO-d_6)

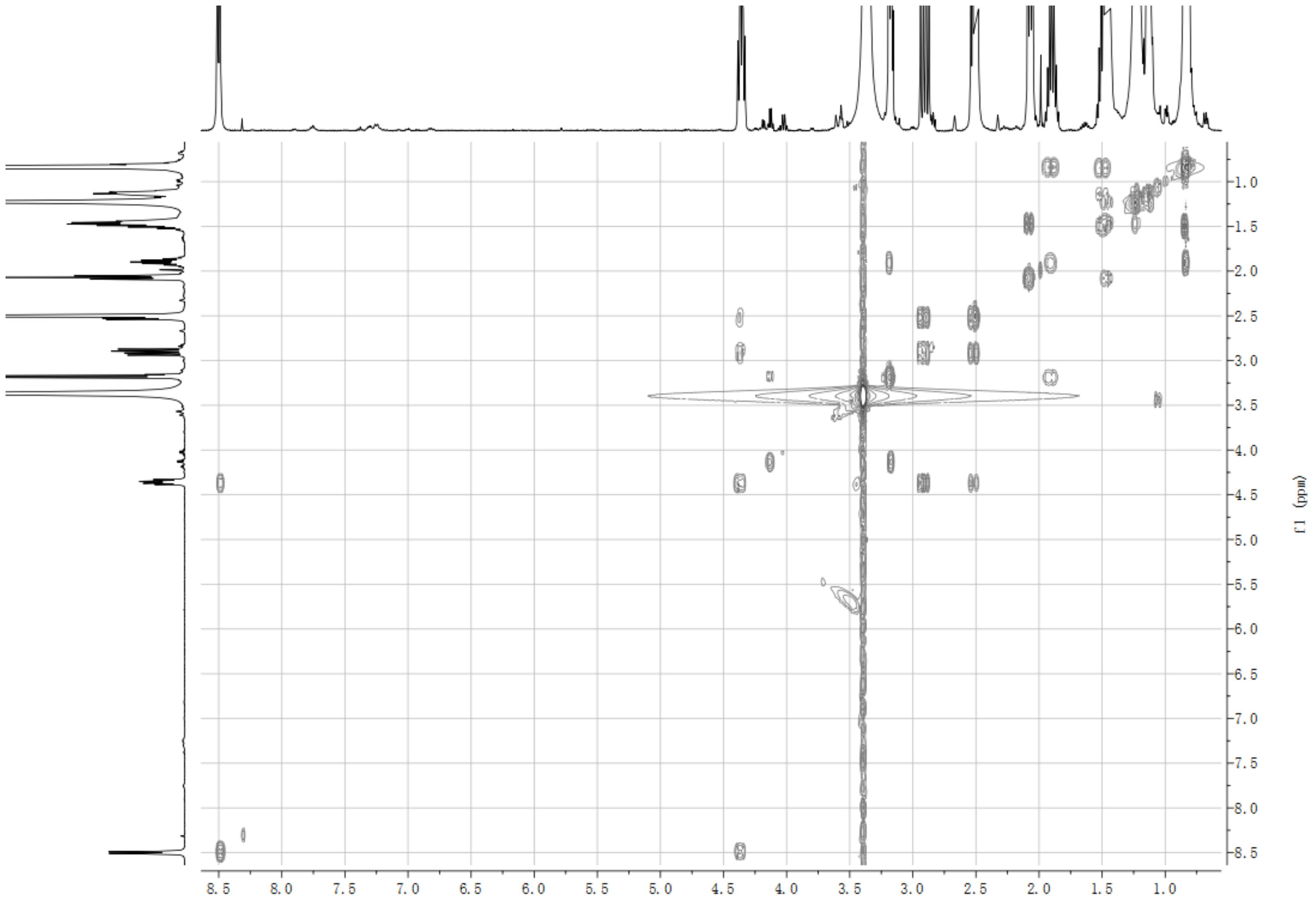

Figure S29. COSY spectrum of compound **4** (400 MHz, DMSO-d_6)

Item name: WM-16 Channel name: Centroided : Combined : Average Time 0.5070 minutes : 1: TOF MS^{E} (100-1000)...
Description:

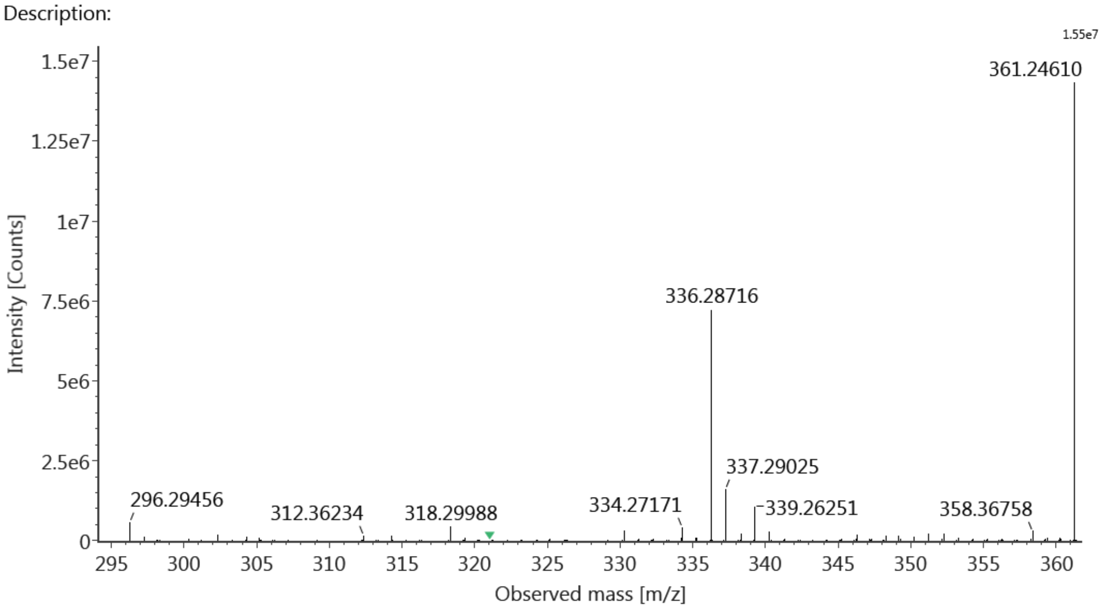

Formula	Calculated Mass	Calculated Mz	Mz	m/z error (mDa)	m/z error (PPM)
$C_{17}H_{34}N_2O_3$	338.2569	361.2467	361.2461	-0.2	-0.7

Figure S30. HR-ESI-MS spectrum of compound **4**

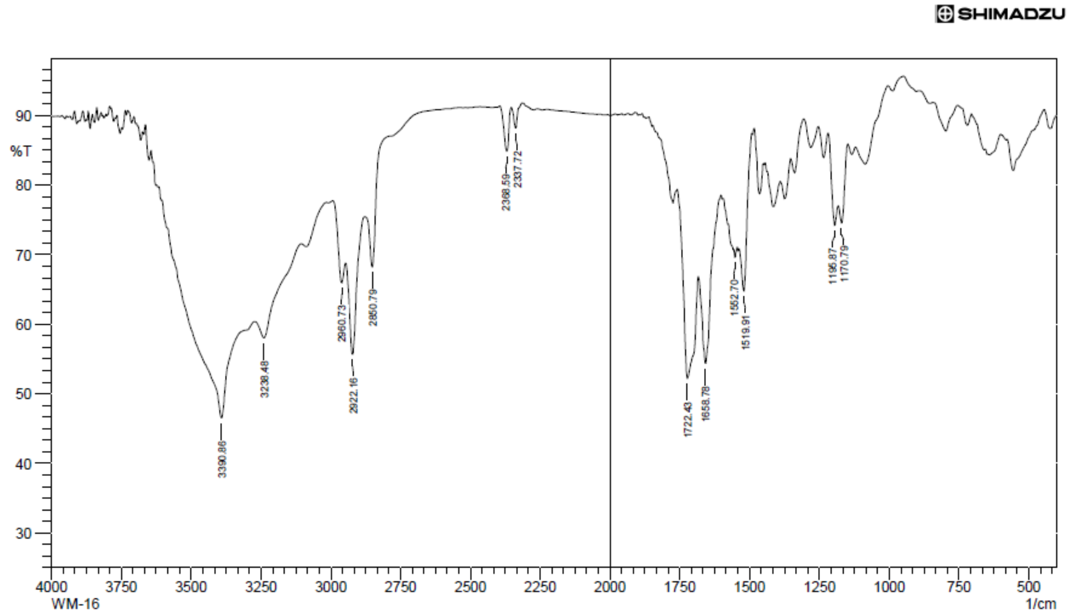

Figure S31. IR spectrum of compound **4**

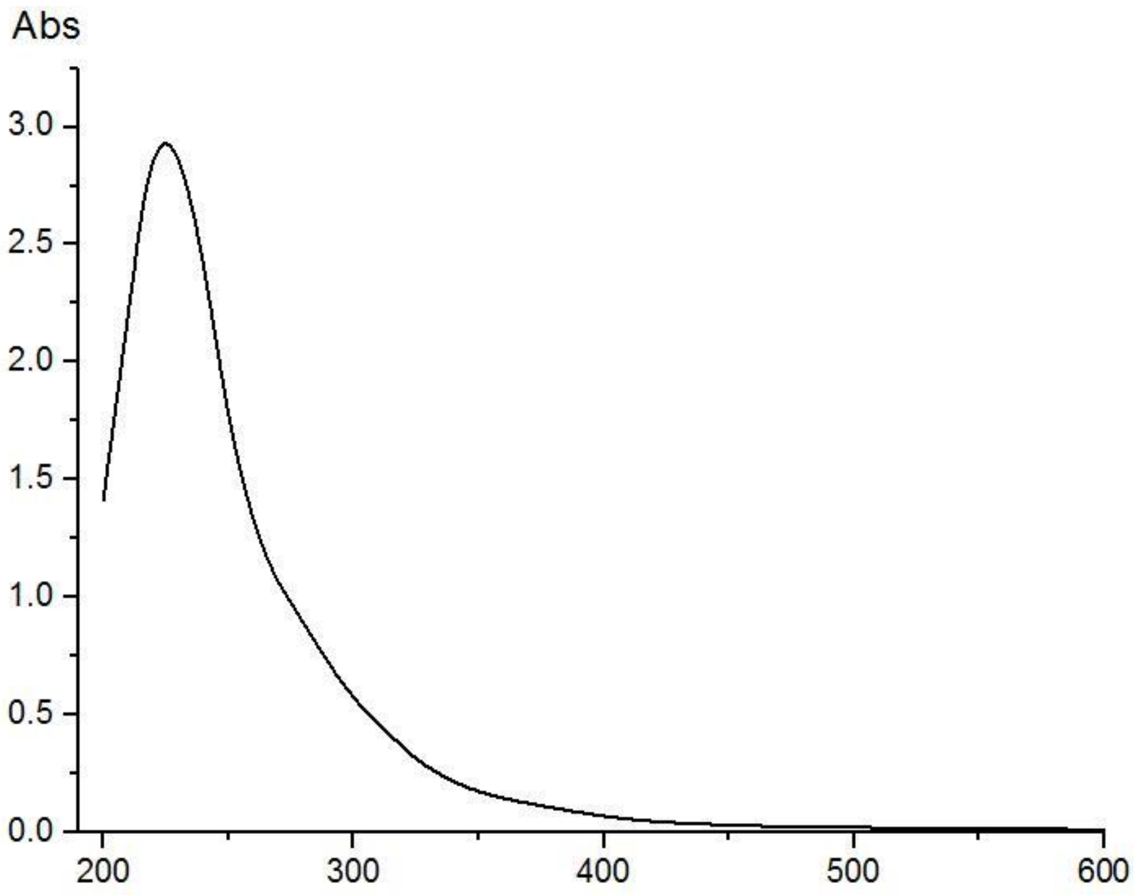

Figure S32. UV spectrum of compound **4**

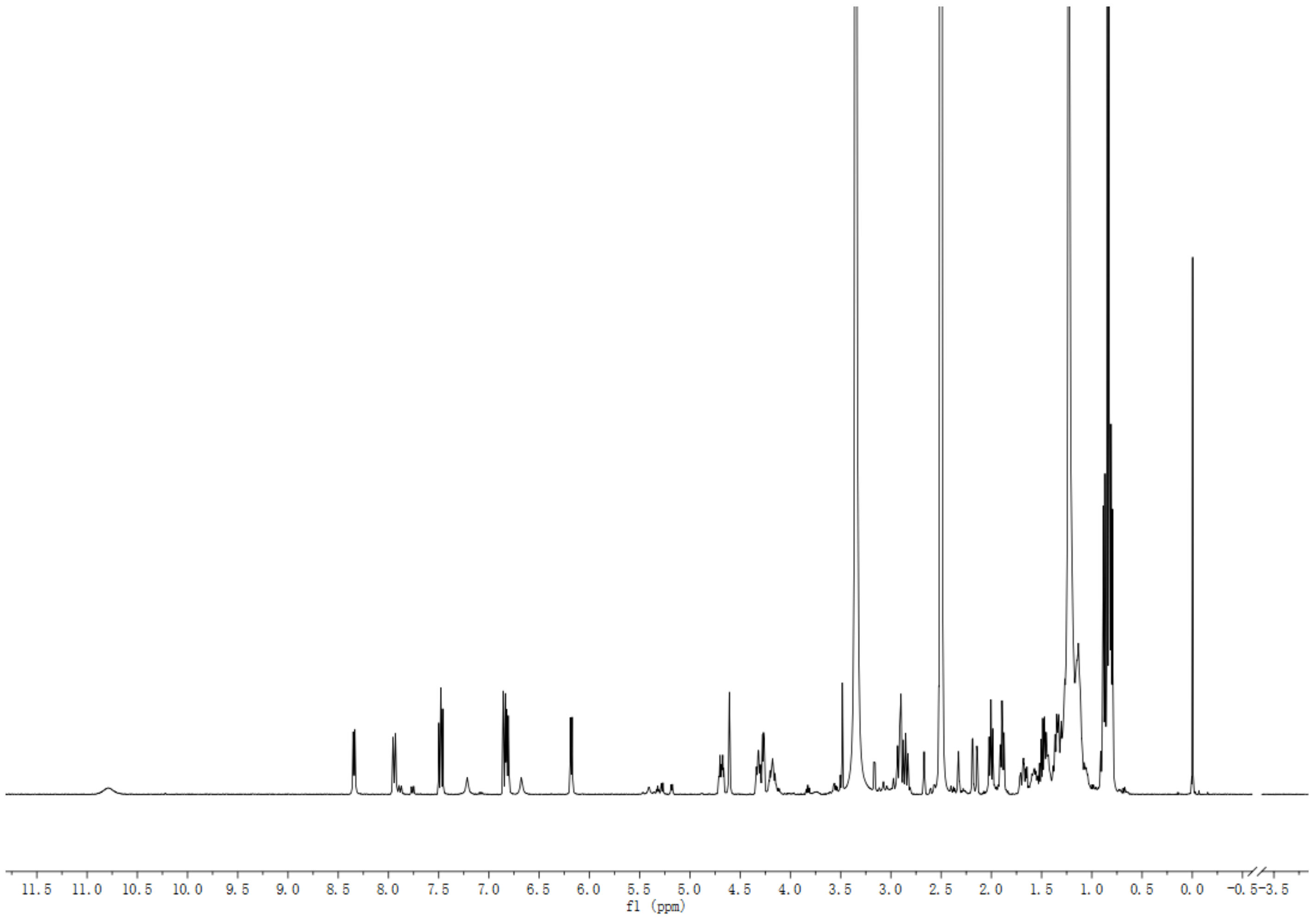

Figure S33. ^{1}H-NMR spectrum of compound **5** (400 MHz, DMSO-*d*6)

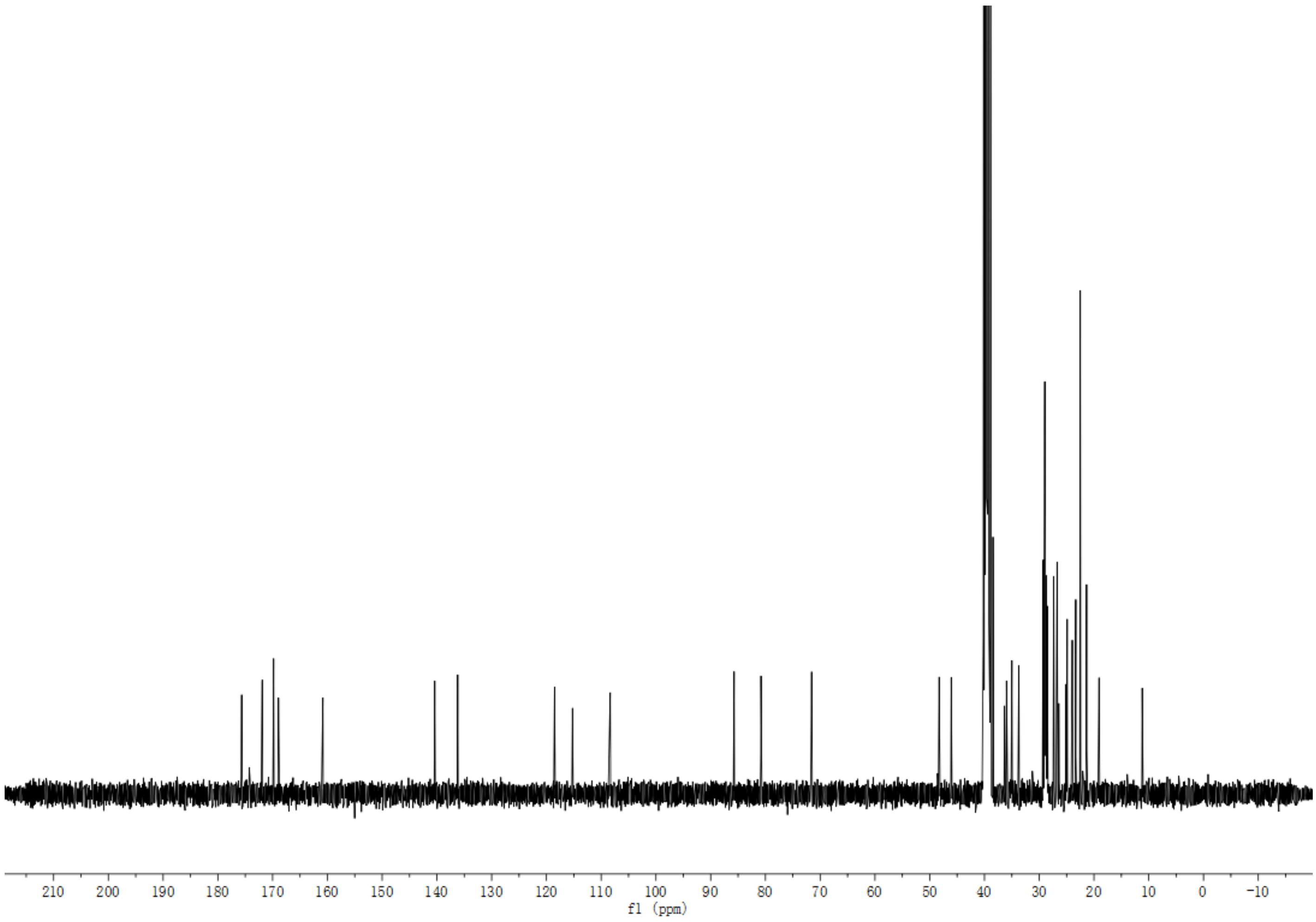

Figure S34. ^{13}C-NMR spectrum of compound **5** (100 MHz, DMSO-d_6)

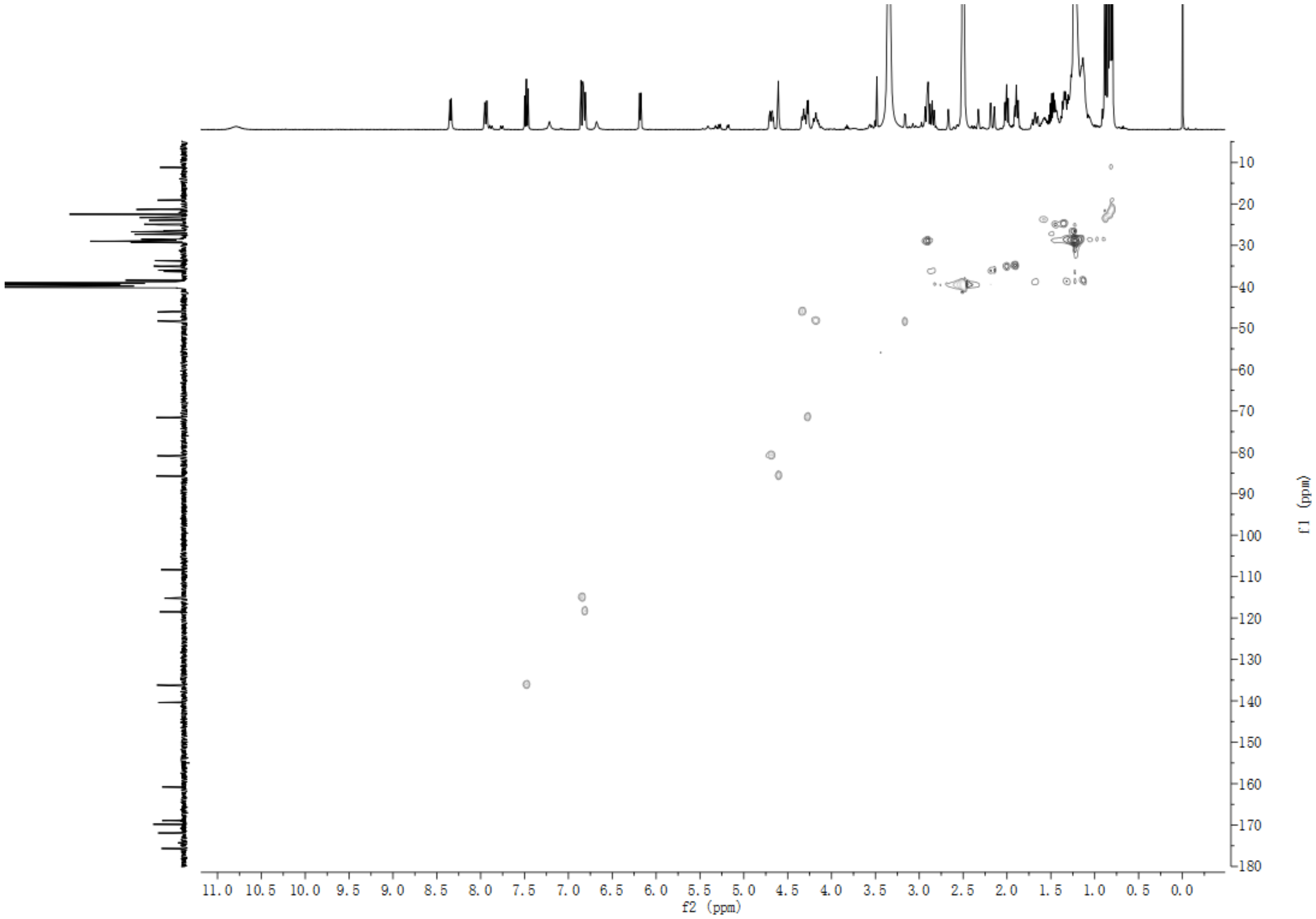

Figure S35. HSQC spectrum of compound **5** (400 MHz, DMSO-d_6)

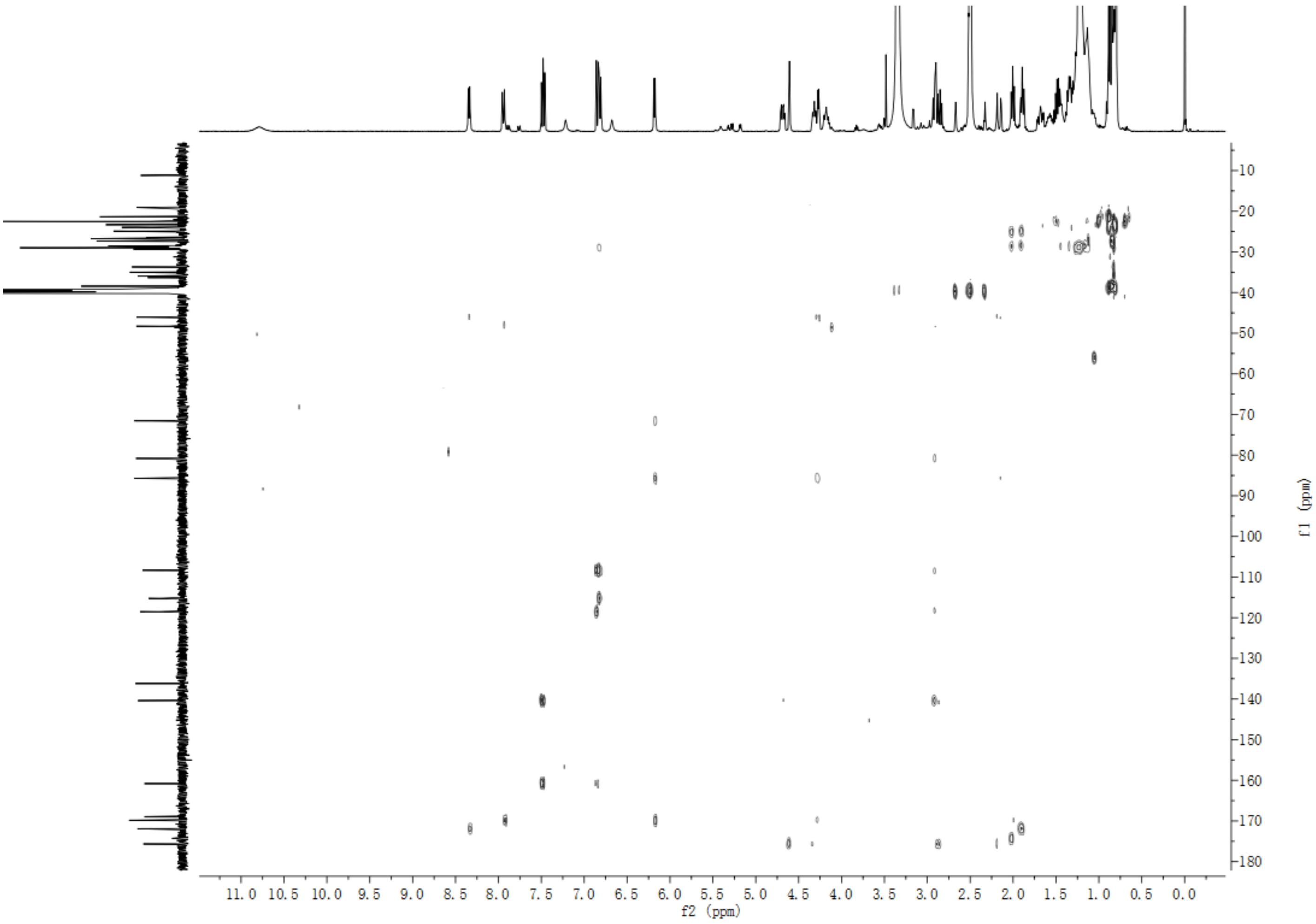

Figure S36. HMBC spectrum of compound **5** (400 MHz, DMSO-d_6)

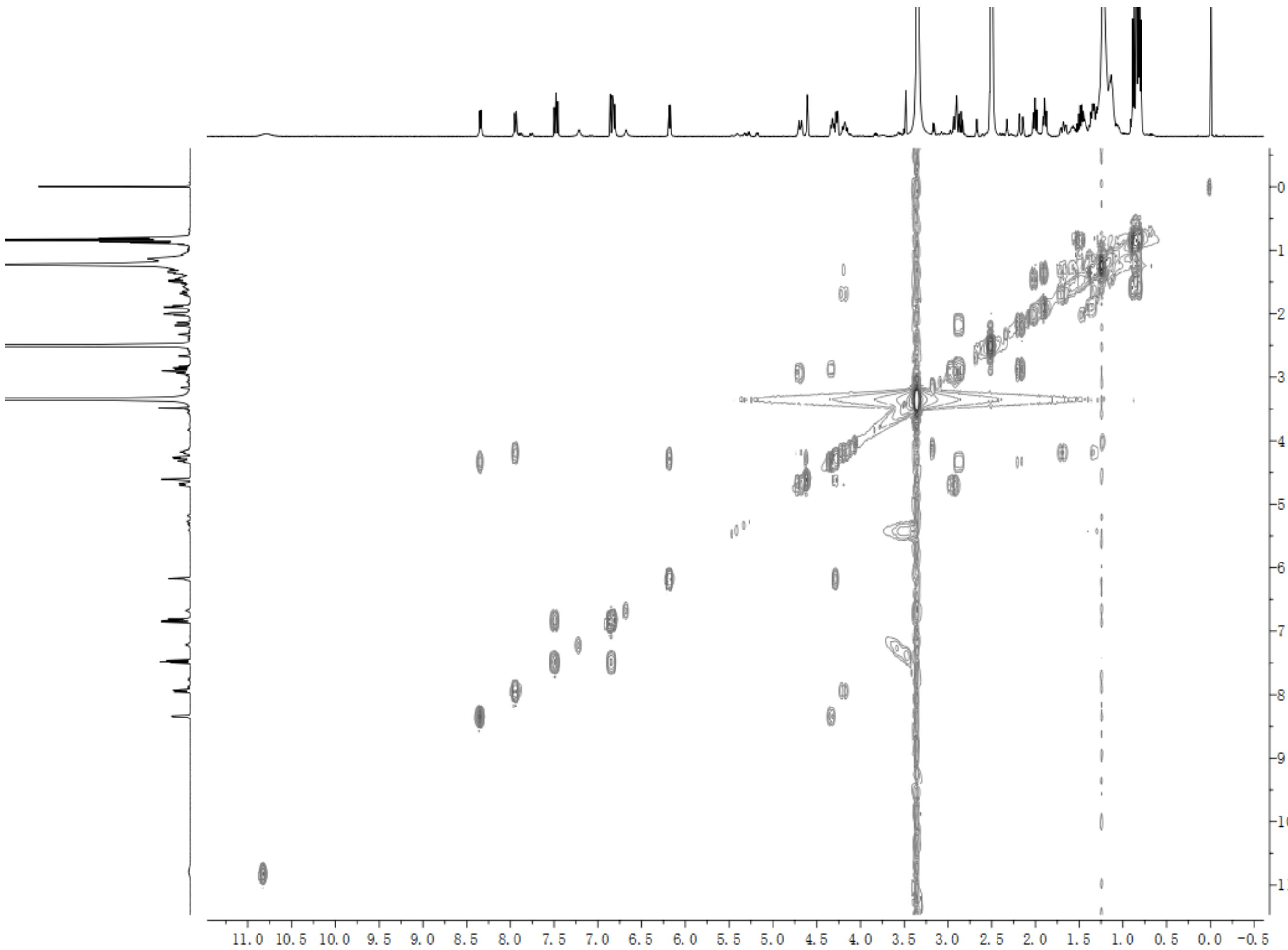

Figure S37. COSY spectrum of compound **5** (400 MHz, DMSO-d_6)

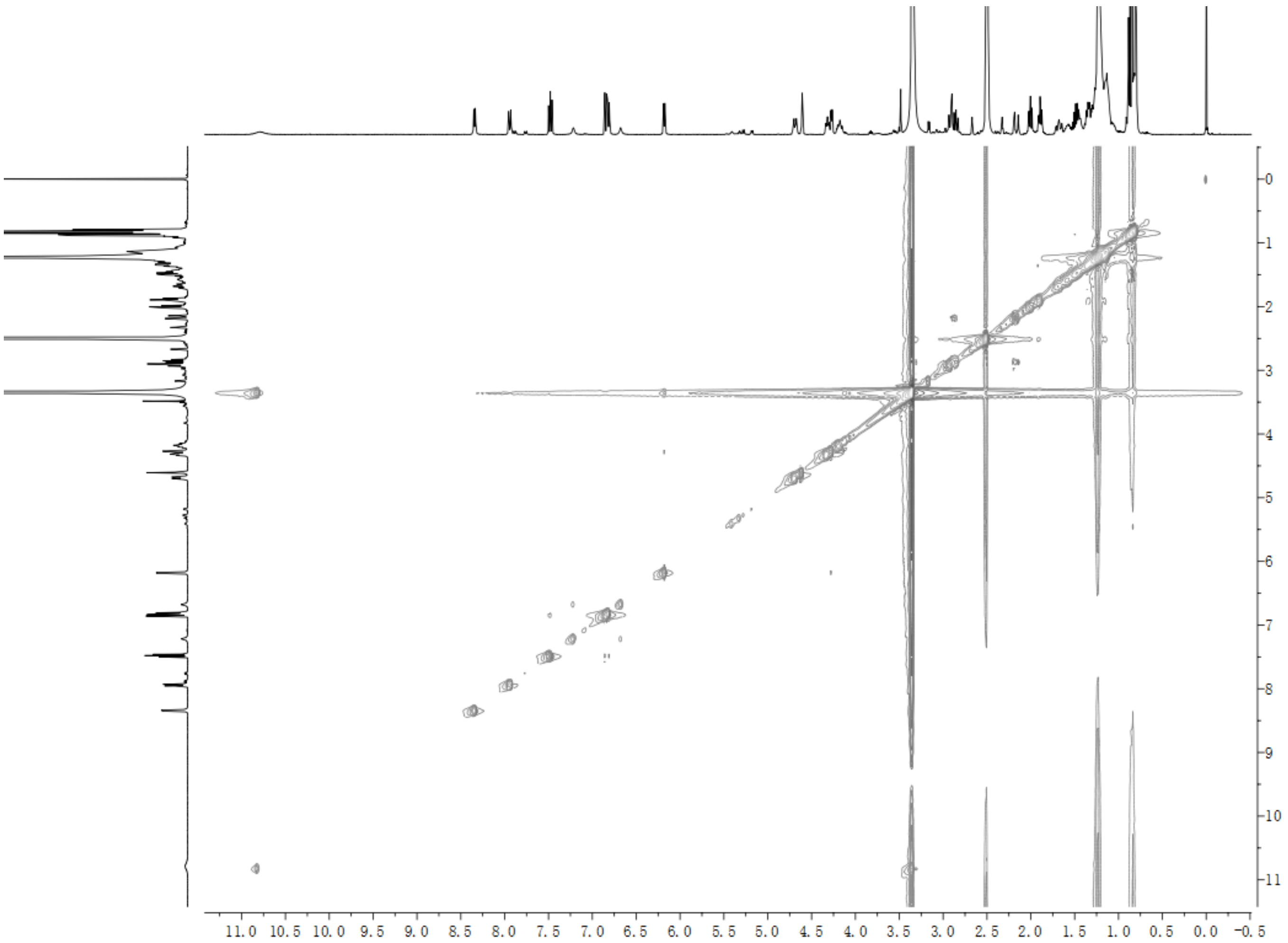

Figure S38. NOESY spectrum of compound **5** (400 MHz, DMSO-d_6)

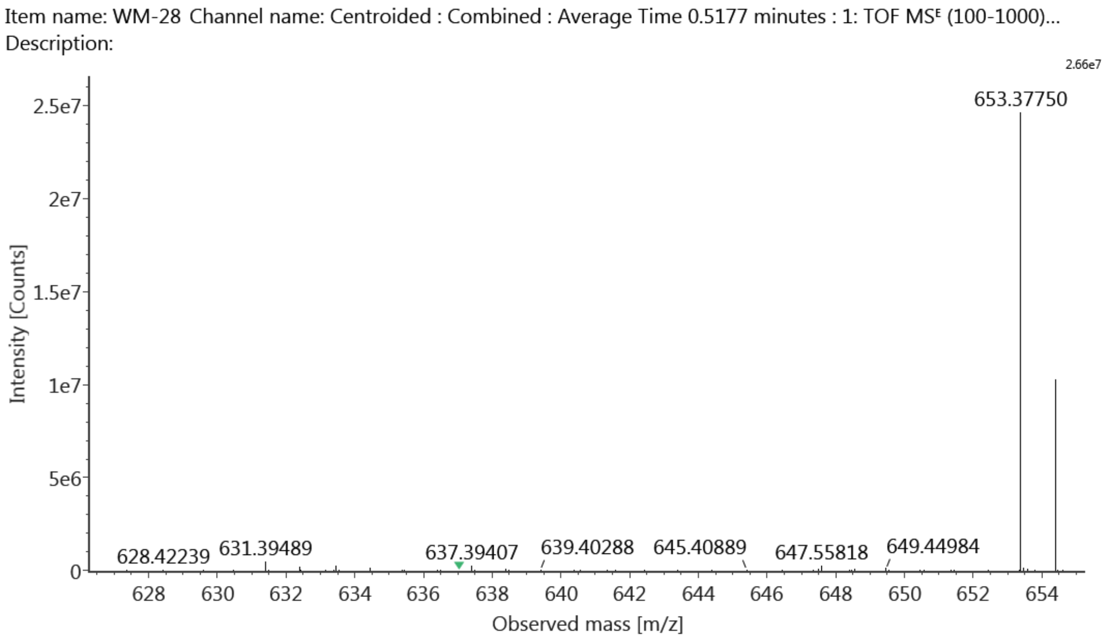

Formula	Calculated Mass	Calculated Mz	Mz	m/z error (mDa)	m/z error (PPM)
$C_{35}H_{54}N_2O_8$	630.3880	653.3778	653.3775	-0.2	-0.9

Figure S39. HR-ESI-MS spectrum of compound **5**

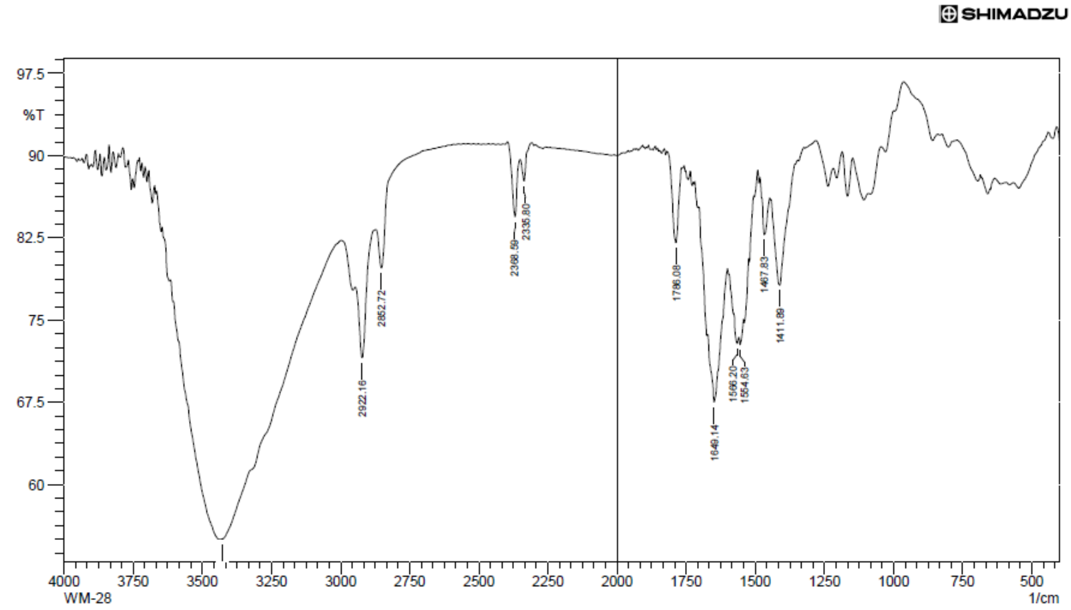

Figure S40. IR spectrum of compound **5**

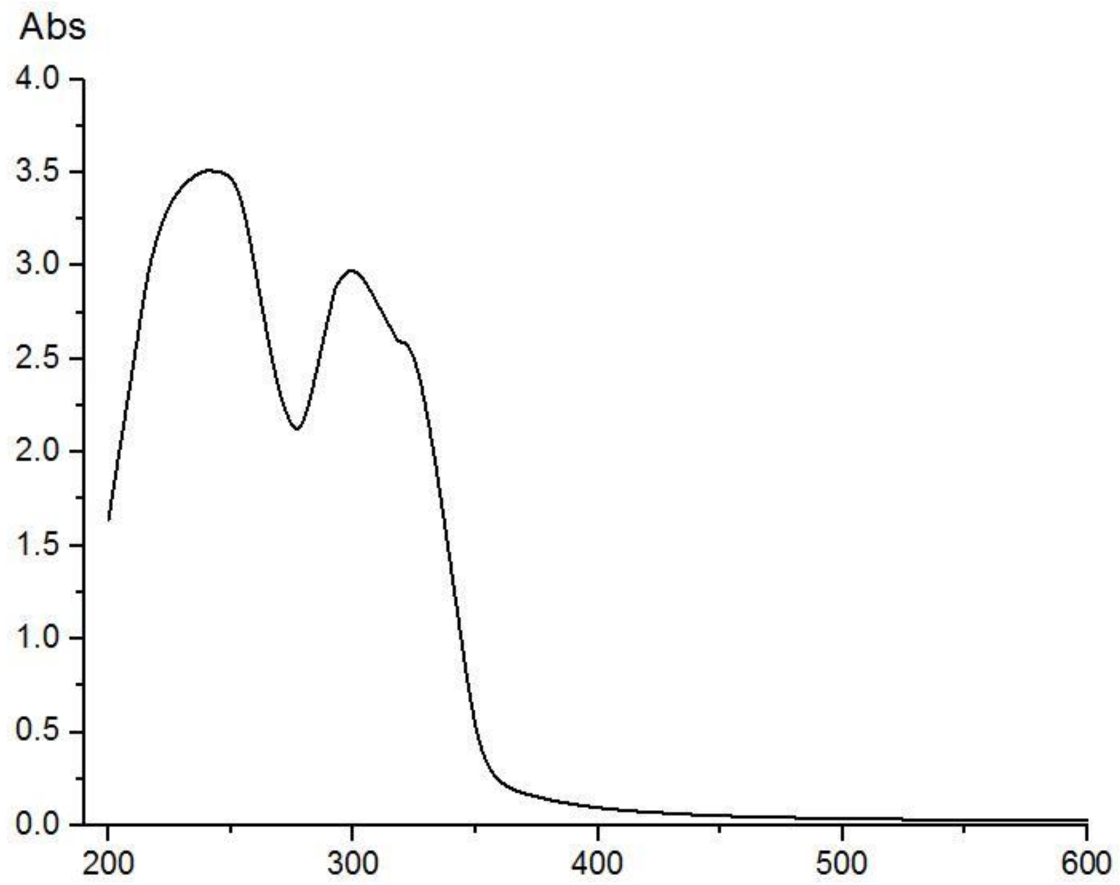

Figure S41. UV spectrum of compound **5**

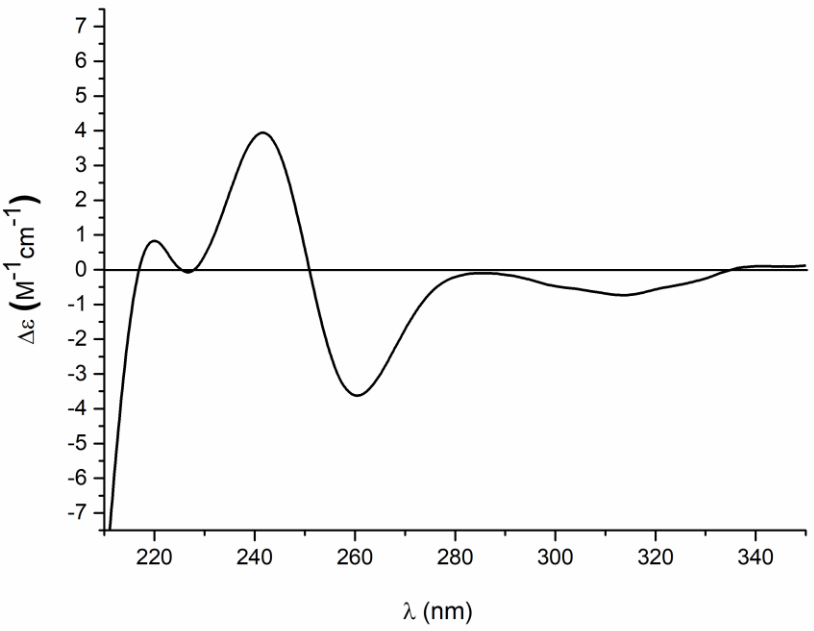

Figure S42. ECD spectrum of compound **5**

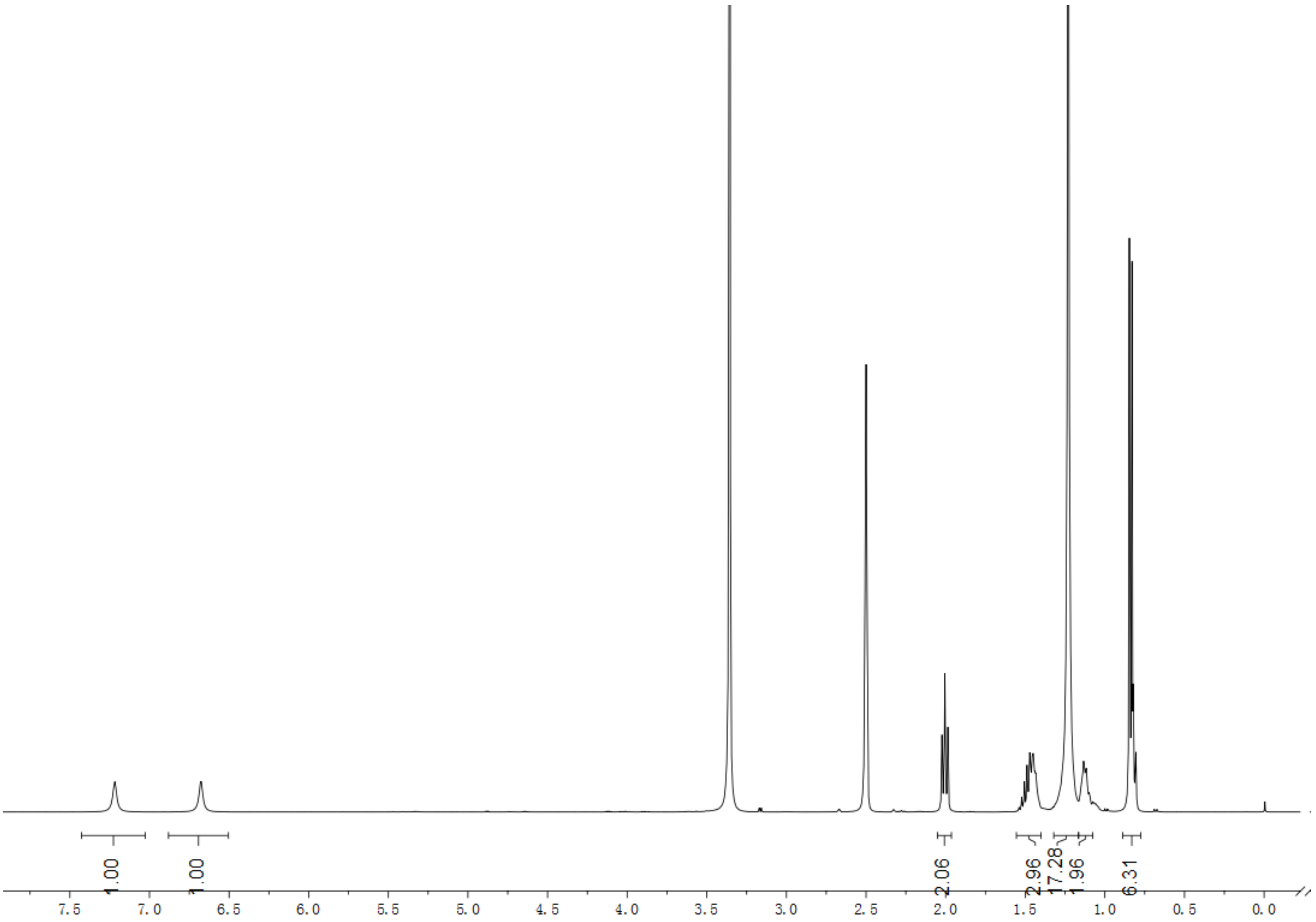

Figure S43. ^{1}H-NMR spectrum of compound **6** (400 MHz, DMSO-d_6)

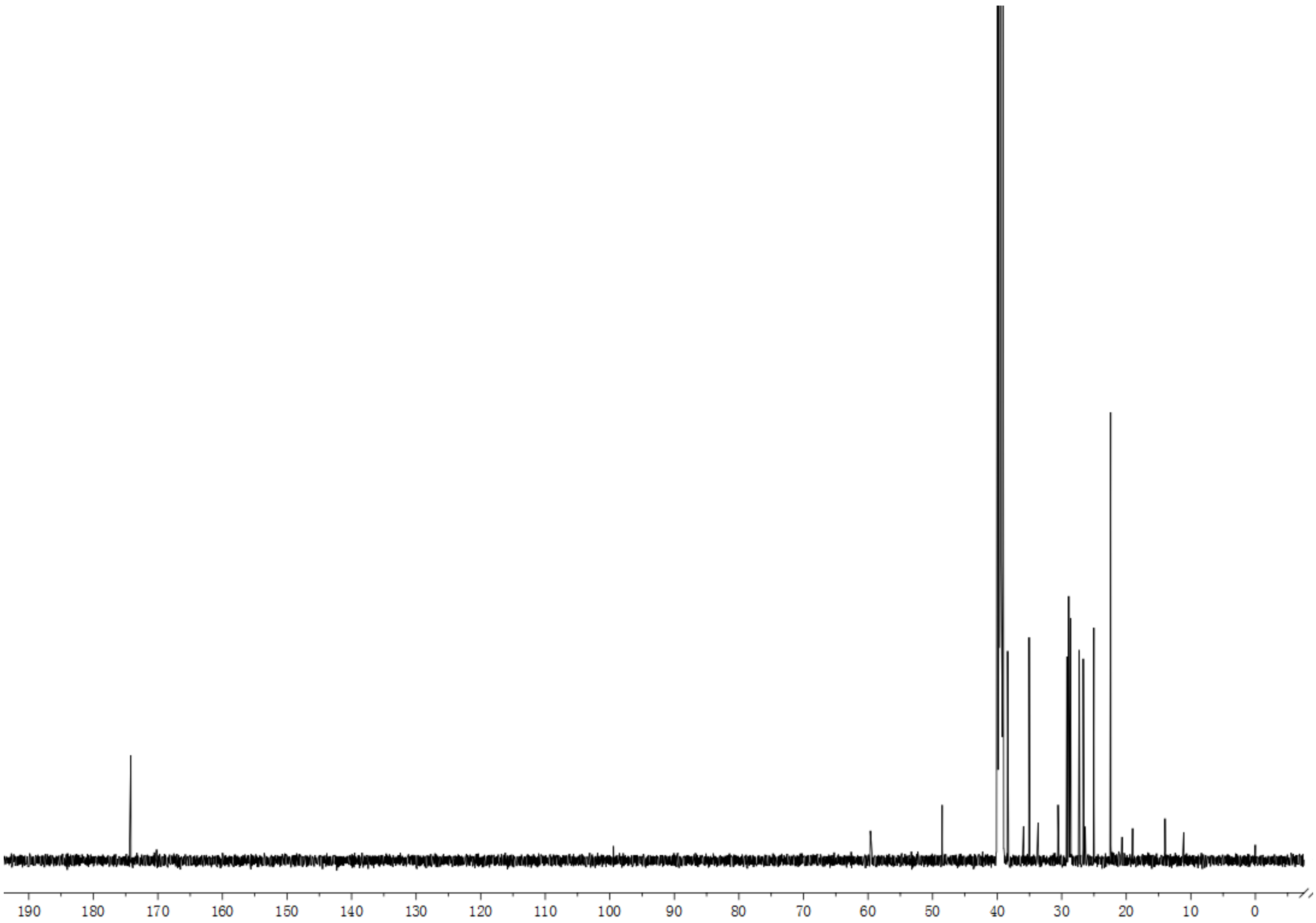

Figure S44. ^{13}C-NMR spectrum of compound **6** (100 MHz, DMSO-d_6)

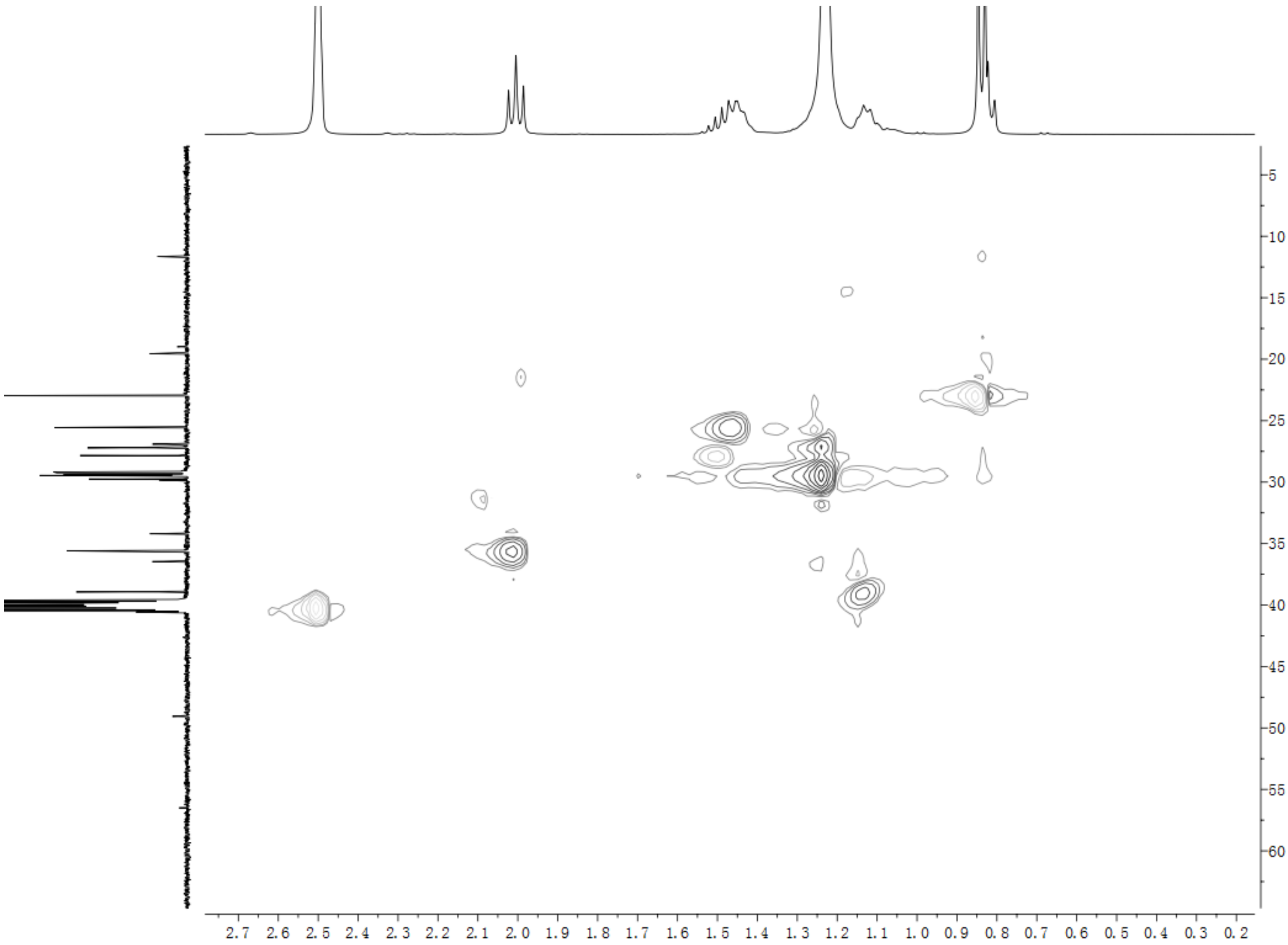

Figure S45. HSQC spectrum of compound **6** (400 MHz, DMSO-d_6)

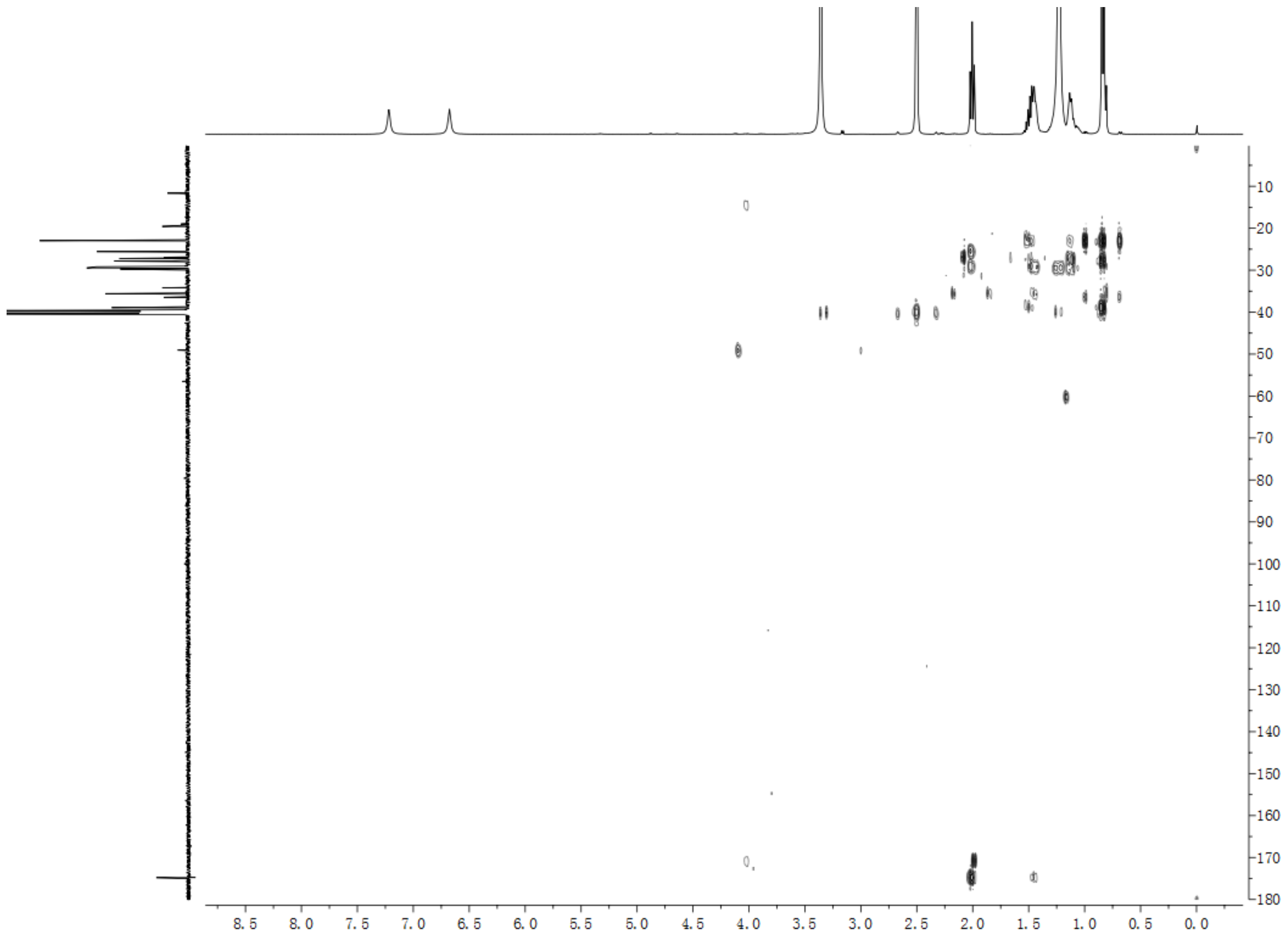

Figure S46. HMBC spectrum of compound **6** (400 MHz, DMSO-d_6)

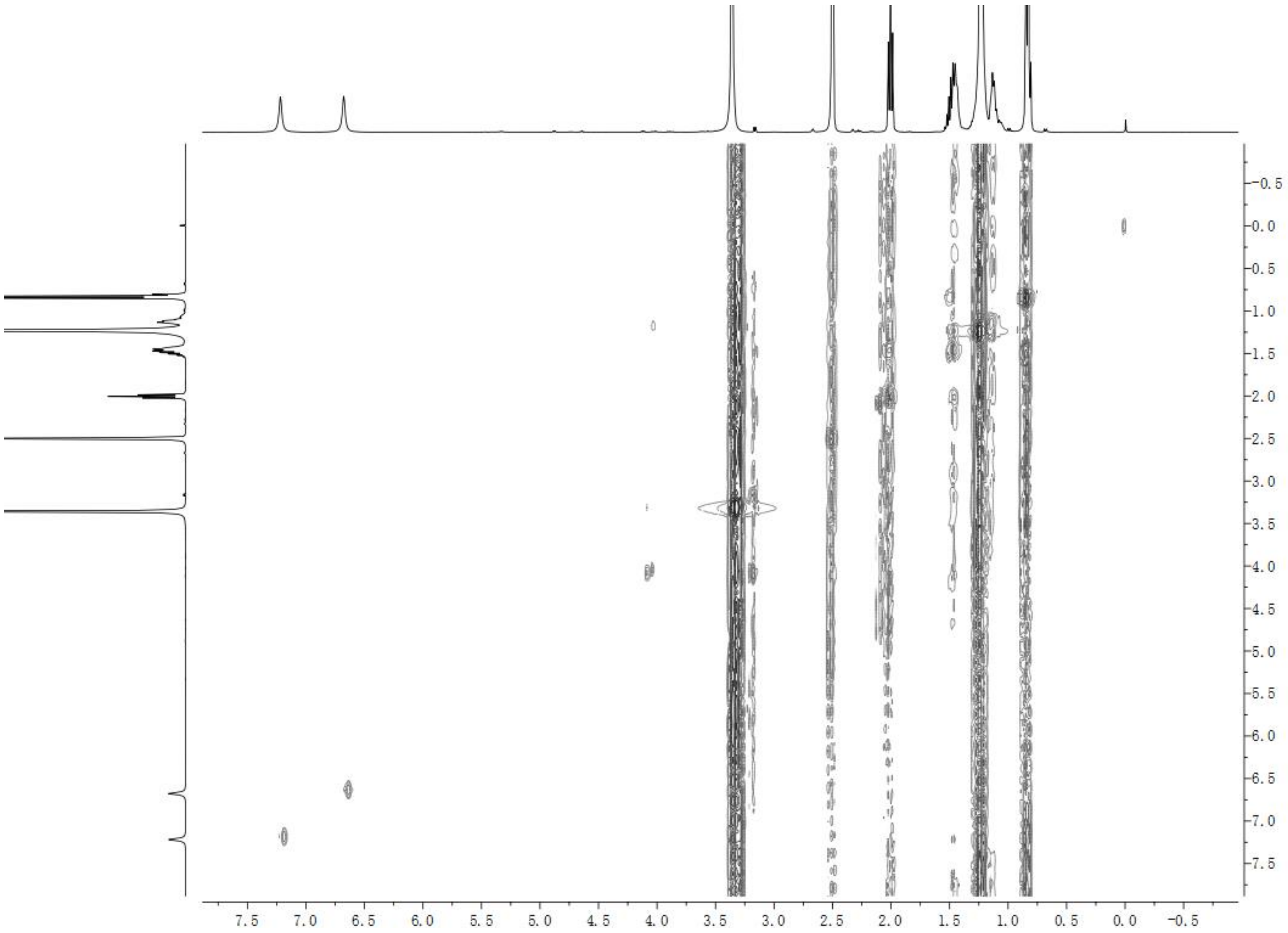

Figure S47. COSY spectrum of compound **6** (400 MHz, DMSO-d_6)

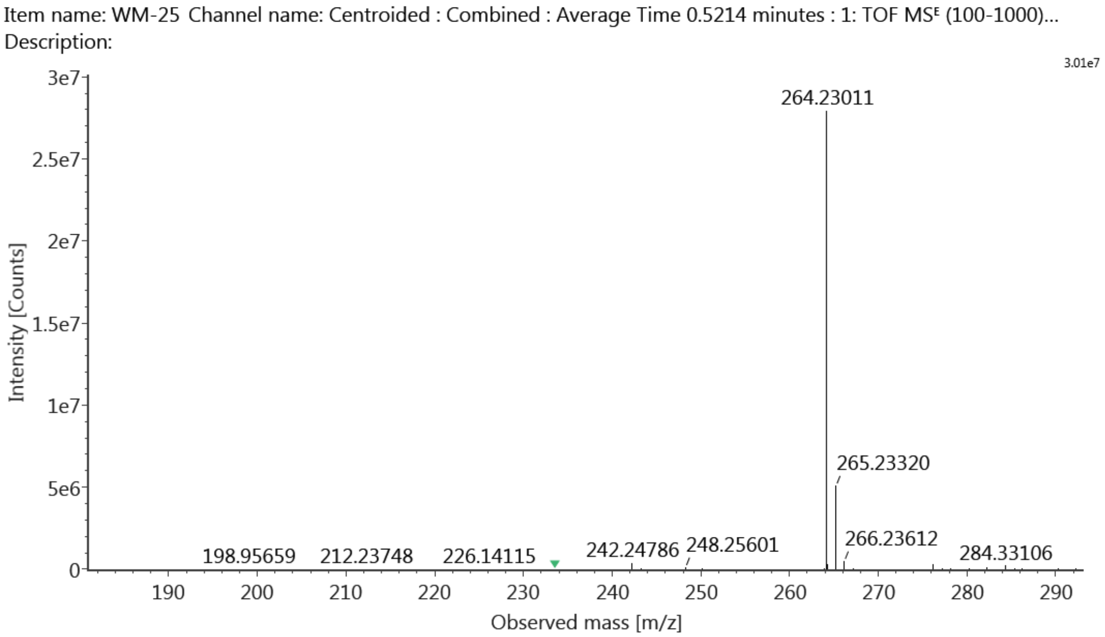

Formula	Calculated Mass	Calculated Mz	Mz	m/z error (mDa)	m/z error (PPM)
$C_{15}H_{31}NO$	241.2406	264.2303	264.2301	-0.01	-0.07

Figure S48. HR-ESI-MS spectrum of compound **6**

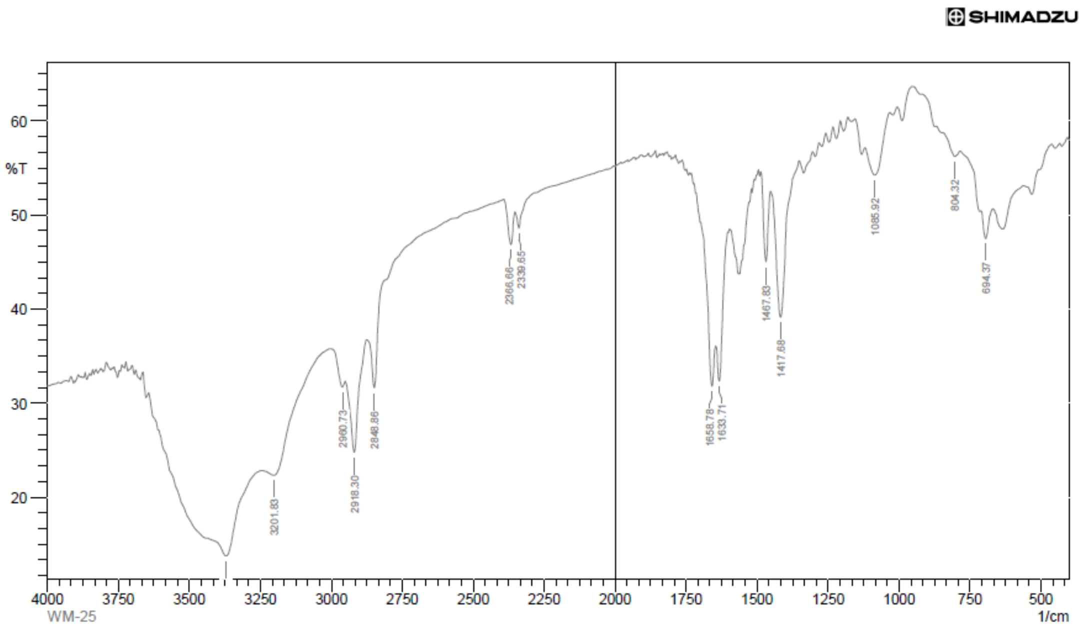

Figure S49. IR spectrum of compound **6**

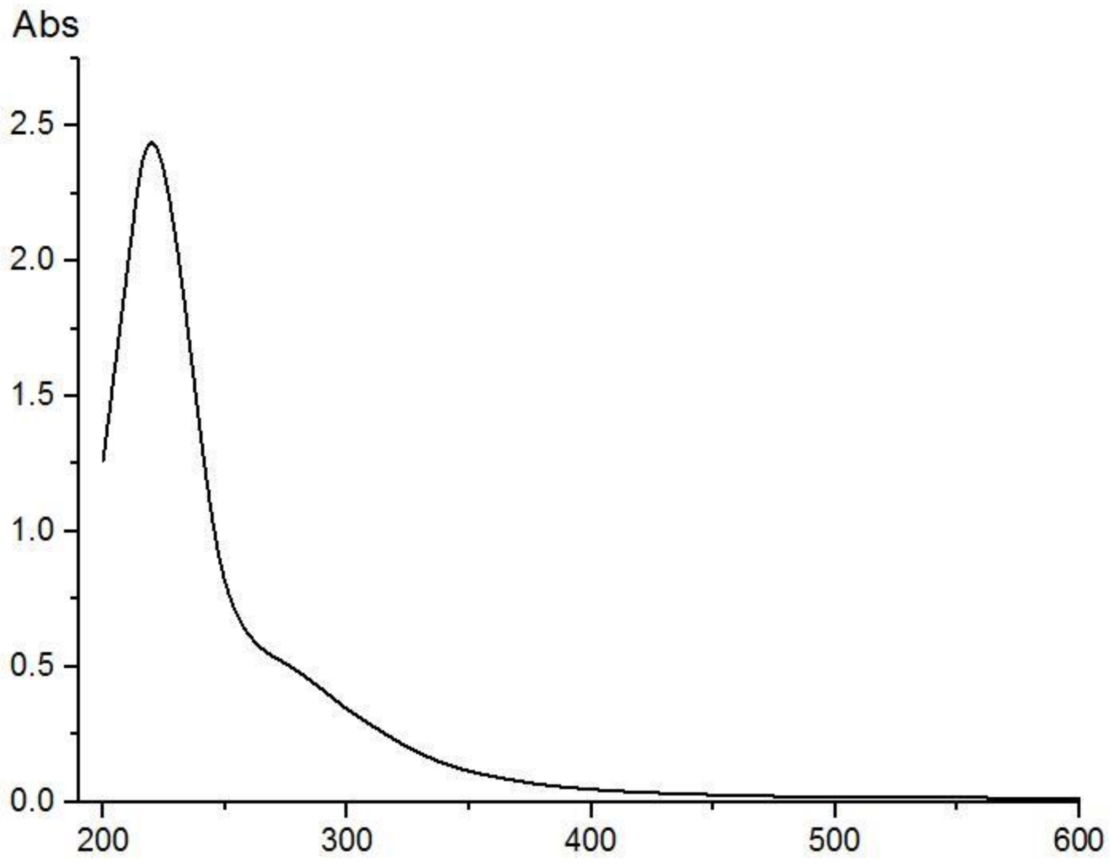

Figure S50. UV spectrum of compound **6**

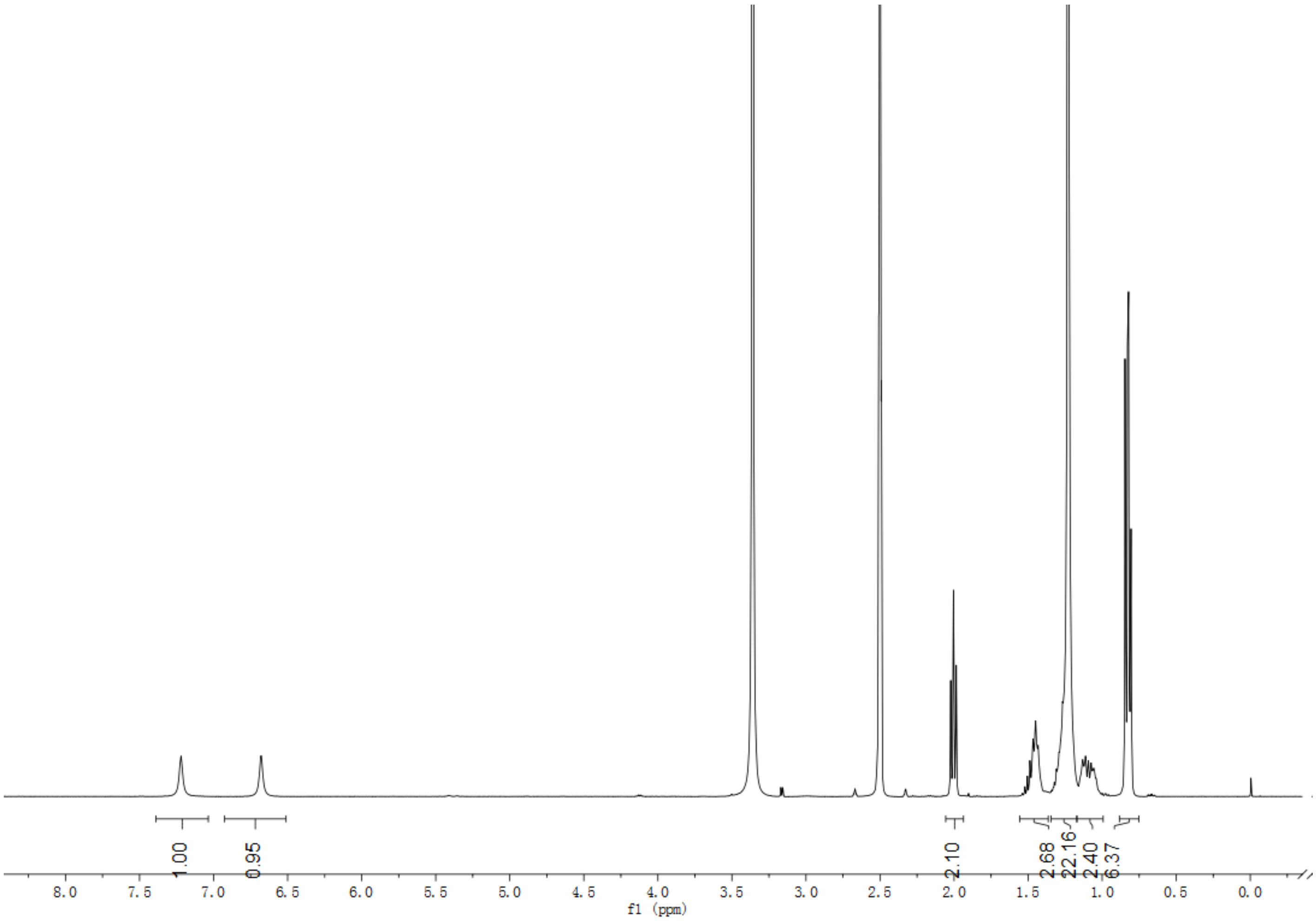

Figure S51. ^{1}H-NMR spectrum of compound **7** (400 MHz, DMSO-d_6)

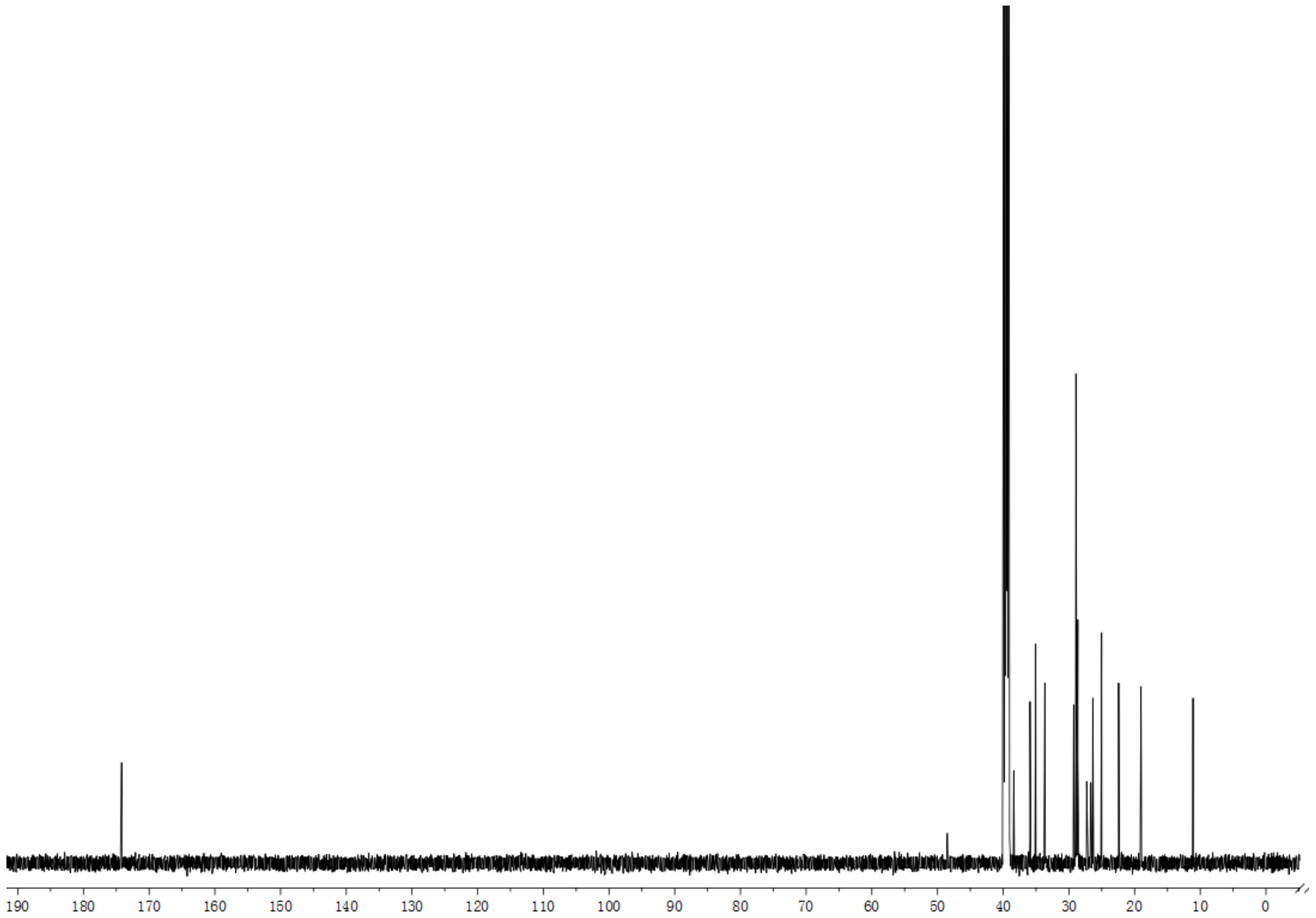

Figure S52. ^{13}C-NMR spectrum of compound **7** (100 MHz, DMSO-d_6)

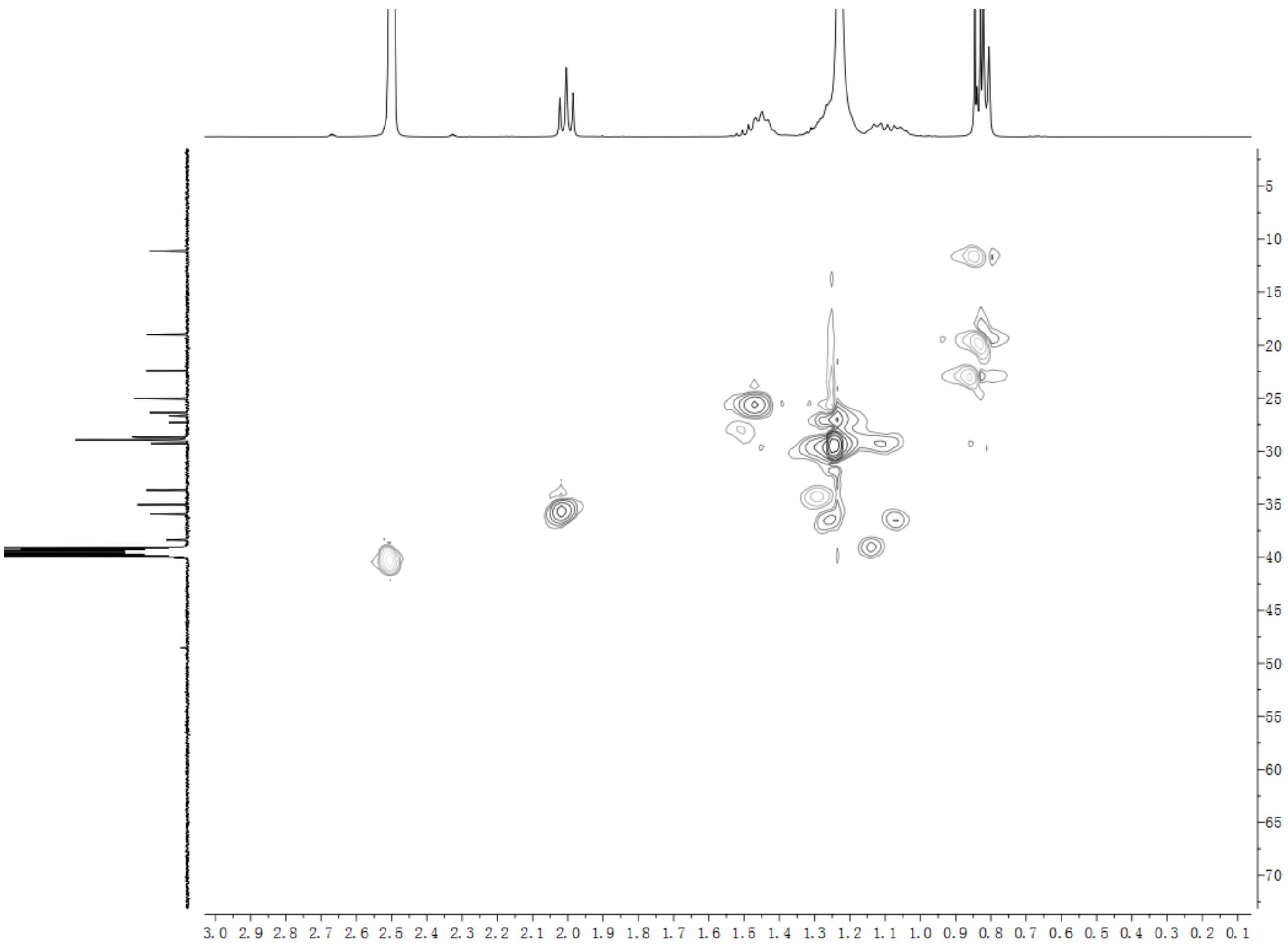

Figure S53. HSQC spectrum of compound **7** (400 MHz, DMSO-d_6)

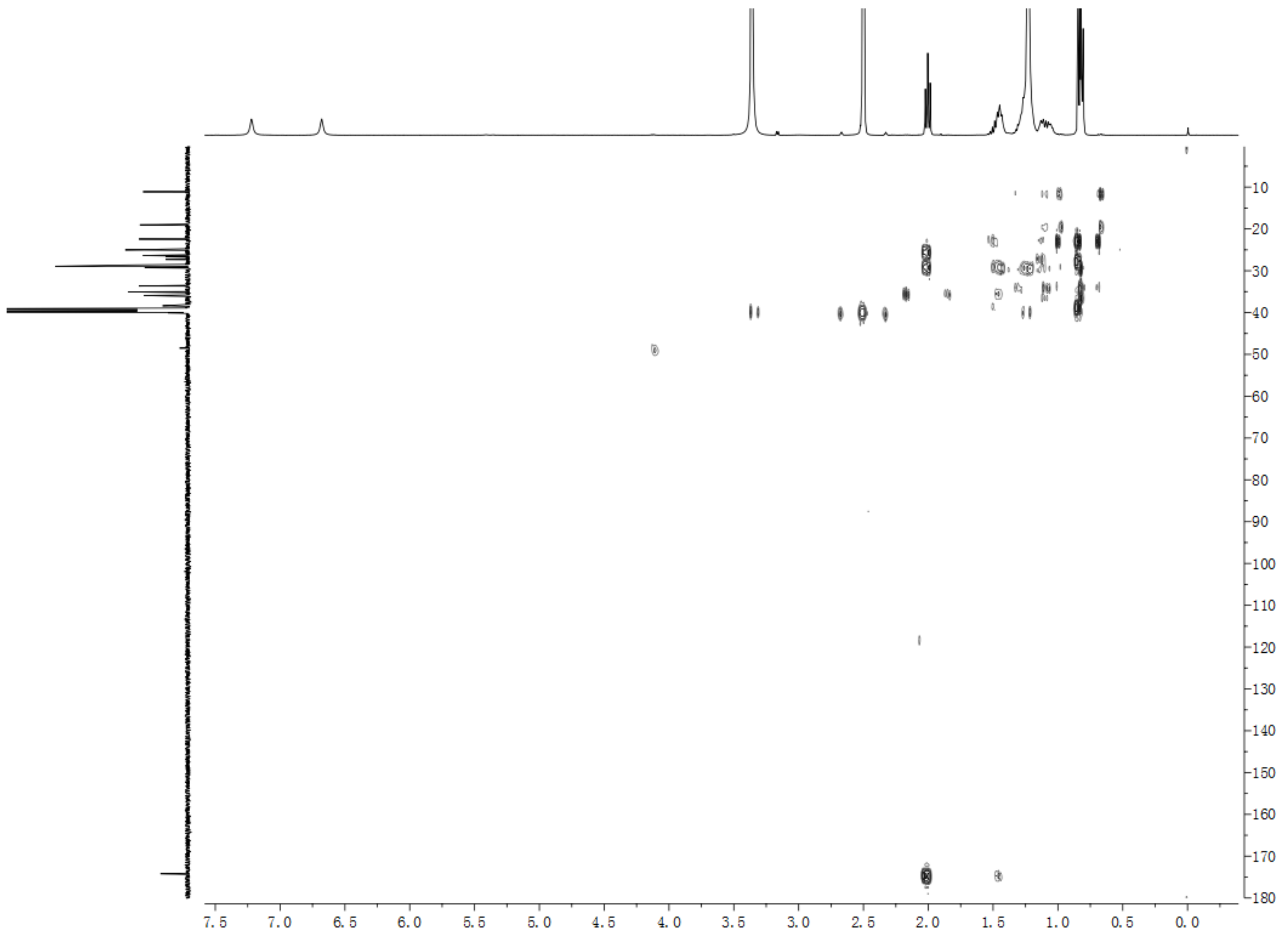

Figure S54. HMBC spectrum of compound **7** (400 MHz, DMSO-d_6)

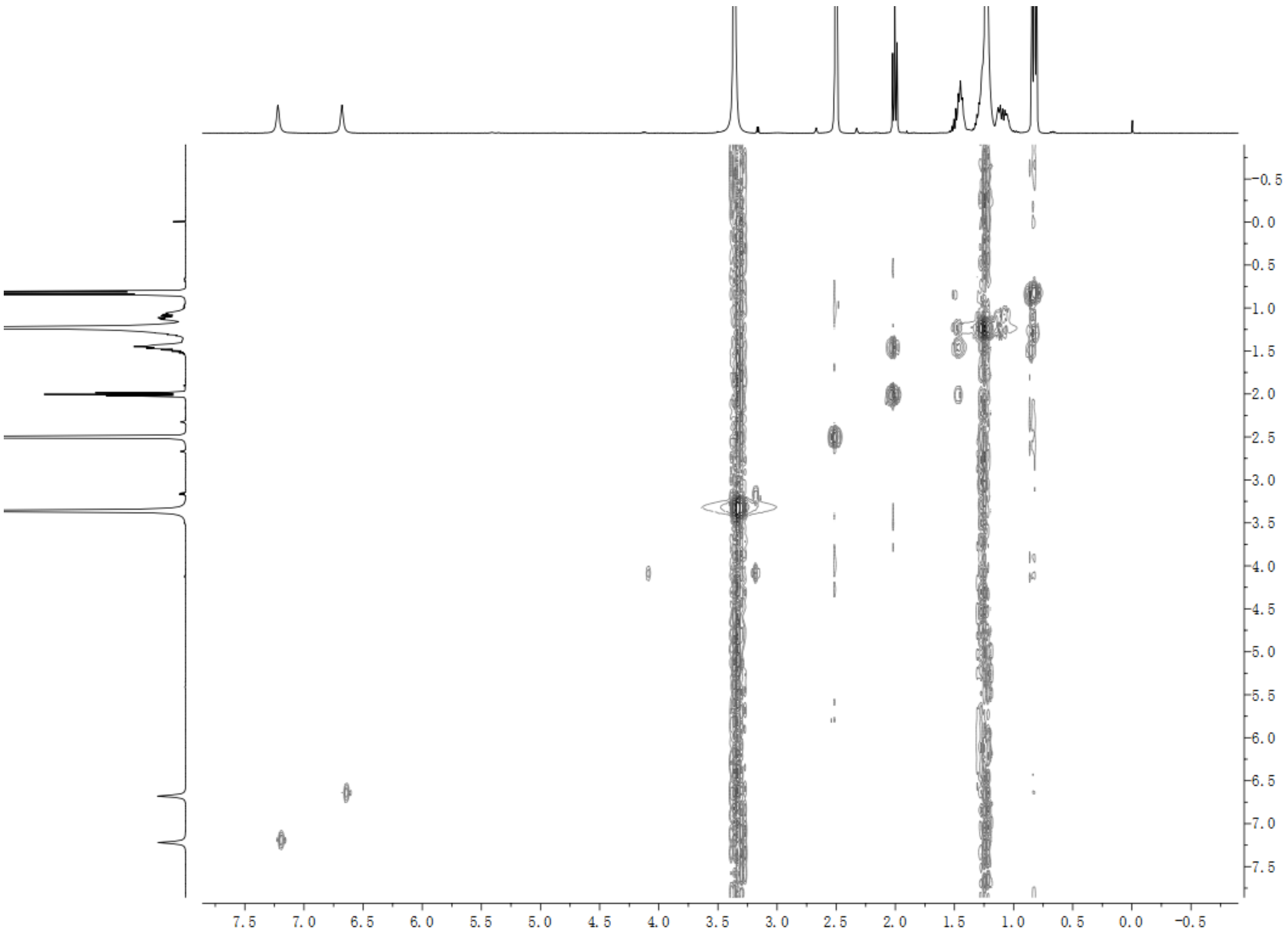

Figure S55. COSY spectrum of compound **7** (400 MHz, DMSO-d_6)

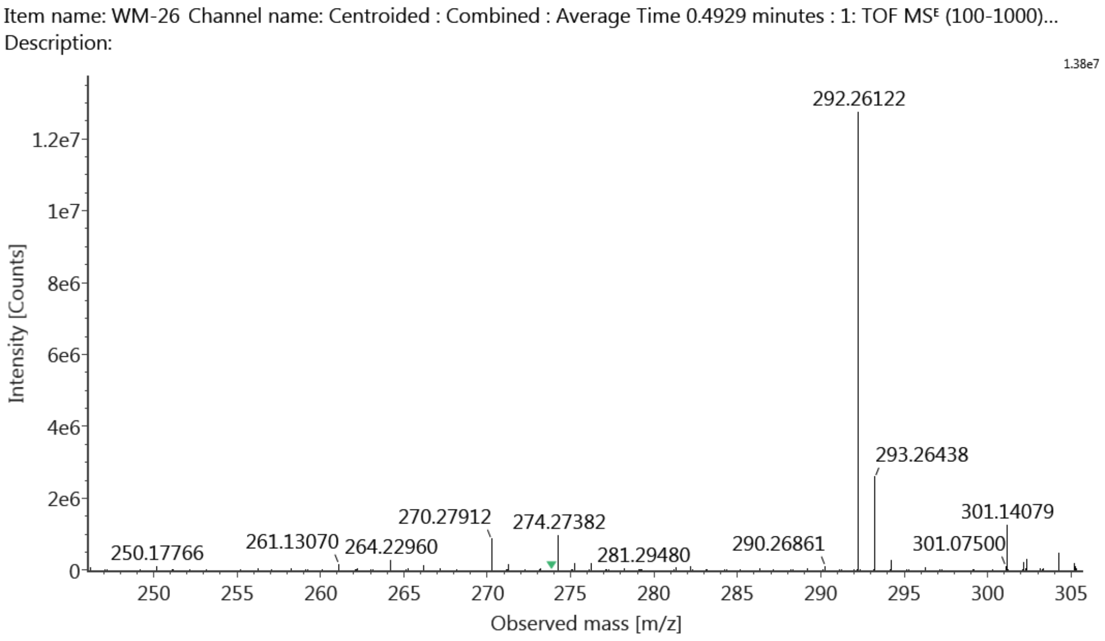

Formula	Calculated Mass	Calculated Mz	Mz	m/z error (mDa)	m/z error (PPM)
$C_{17}H_{35}NO$	269.6719	292.2616	292.2612	-0.02	-0.07

Figure S56. HR-ESI-MS spectrum of compound **7**

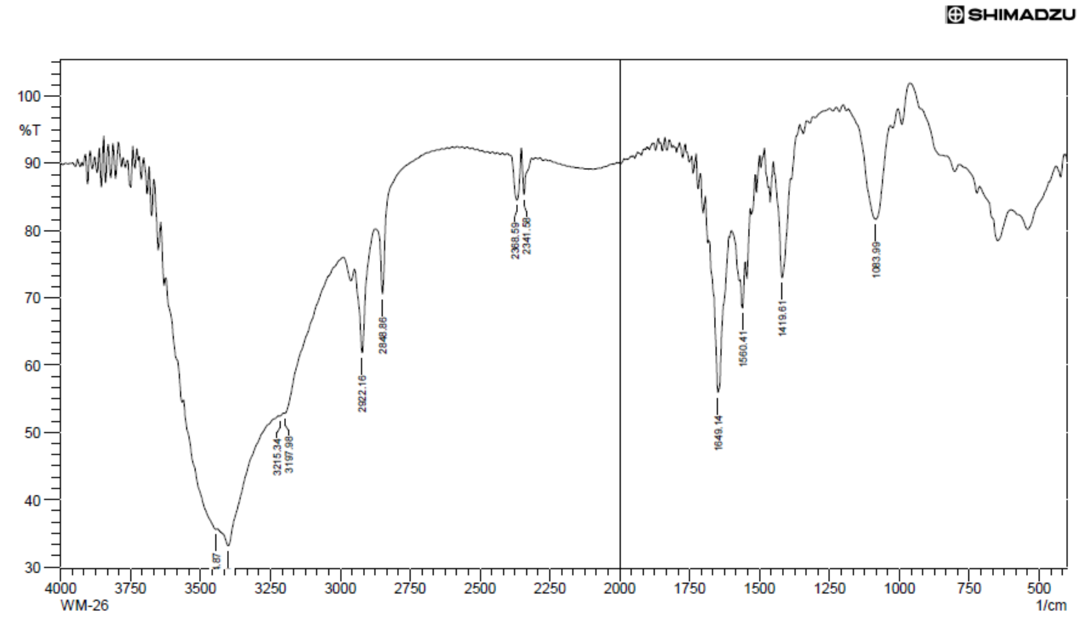

Figure S57. IR spectrum of compound **7**

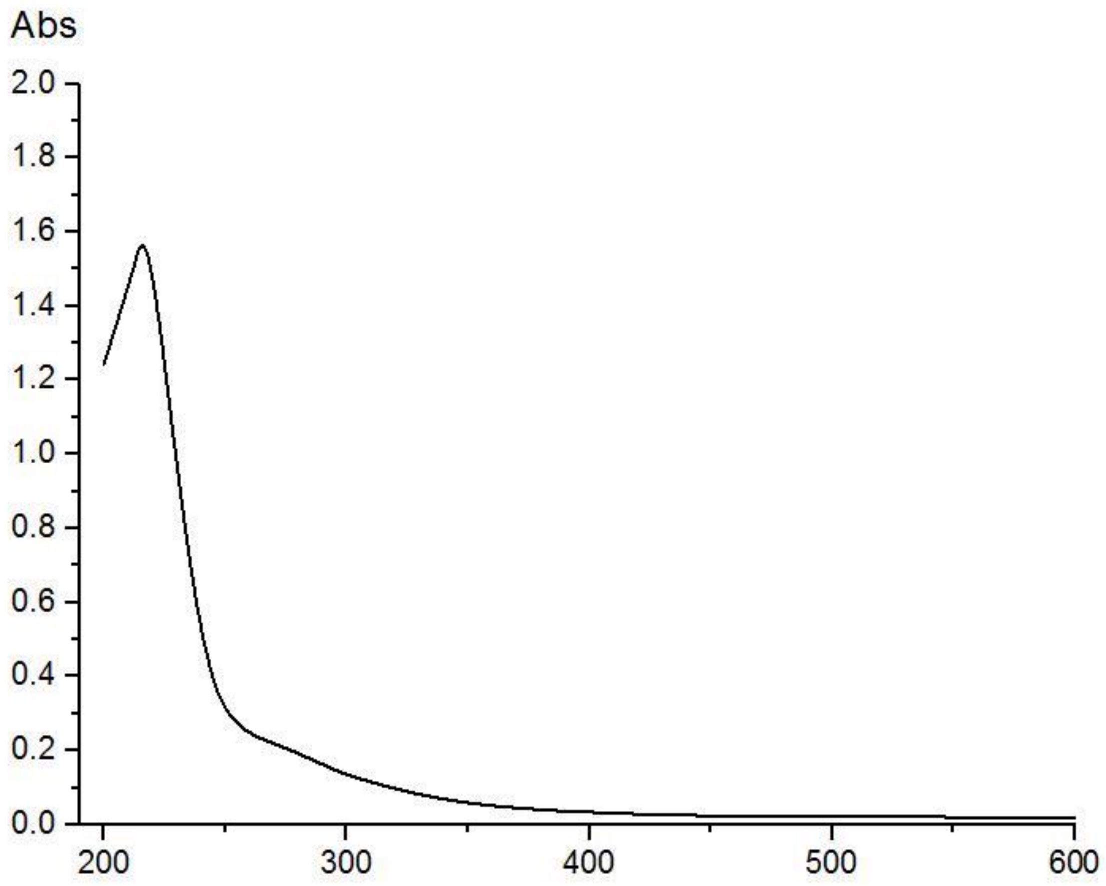

Figure S58. UV spectrum of compound **7**

Table S1 ^{1}H and ^{13}C-NMR data (400 and 100 MHz, in DMSO-d_6) of **6** and **7**.

	**6**		**7**	
Position	δ_H (*J*, Hz)	δ_C	δ_H (*J*, Hz)	δ_C
1-NH	6.68, s		6.68, s	
	7.22, s		7.22, s	
2		174.2		174.2
3	2.00, t (7.2)	35.1	2.00, t (7.2)	35.0
4	1.48, m	25.0	1.48, m	25.0
5	1.23, m	28.6	1.23, m	28.6
6	1.23, m	28.7	1.23, m	28.7
7	1.23, m	28.9	1.23, m	28.8
8	1.23, m	28.9	1.23, m	28.8
9	1.23, m	28.9	1.23, m	28.9
10	1.23, m	29.0	1.23, m	28.9
11	1.23, m	29.2	1.23, m	29.0
12	1.23, m	26.7	1.23, m	29.3
13	1.23, m	38.4	1.23, m	26.4
14	1.48 (1H, m)	27.3	1.09, m; 1.27 m	36.0
15	0.84, d (6.4)	22.5	1.30, m	33.7
16	0.84, d (6.4)	22.5	1.08, m	29.0
17			0.83, t (9.0)	11.2
18			0.78, d (9.0)	19.1

Spectral Data of 6 and 7

Bacillamidin F (**6**): amorphous, white powder; UV (MeOH) λ_{max} (log ε) 223 (2.47) nm; IR (KBr) ν_{max} 3202, 2961, 2849, 1659, 1634, 1468, 1418, 1086 cm^{-1}; ^{1}H and ^{13}C NMR data, Table 1; HRESIMS *m*/*z* 264.2301 [M + Na]$^{+}$ (calcd. for $C_{15}H_{31}NONa$, 264.2303).

Bacillamidin G (**7**): amorphous, white powder; UV (MeOH) λ_{max} (log ε) 223 (2.45) nm; IR (KBr) ν_{max} 3180, 2922, 2849, 1649, 1560, 1420, 1084 cm^{-1}; ^{1}H and ^{13}C NMR data, Table 1; HRESIMS *m*/*z* 292.2612 [M + Na]$^{+}$ (calcd. for $C_{17}H_{35}NONa$, 292.2616).
